# Controlling Selectivity at the First Hydrogenation Level: Synthesis of 3‐Hydroxyisoindolines by Heterogeneously Silver‐Catalyzed Monohydrogenation of Phthalimides

**DOI:** 10.1002/advs.202516161

**Published:** 2025-10-15

**Authors:** Carles Lluna‐Galán, Rosa Adam, Jose R. Cabrero‐Antonino

**Affiliations:** ^1^ Instituto de Tecnología Química Universitat Politécnica de València‐Consejo Superior Investigaciones Científicas (UPV‐CSIC) Avda. de los Naranjos s/n València 46022 Spain; ^2^ Departament de Química Orgànica, Facultat de Farmàcia Universitat de València Av. Vicent Andrés Estellés s/n, Burjassot València 46100 Spain

**Keywords:** 3‐hydroxyisoindolinones, heterogeneous catalysis, phthalimides, selective hydrogenation, silver

## Abstract

The development of robust nanocatalysts able to mediate important organic transformations with high levels of selectivity constitutes a hot topic in fine‐chemistry and catalysis fields. In this context, the rational design of heterogeneous catalysts featuring suitable active sites for obtaining 3‐hydroxyisoindolinones by the direct monohydrogenation of highly accessible phthalimides is an attractive approach. This work presents the design and successful catalytic application of a robust and recyclable [Ag/Al_2_O_3_] nanomaterial capable to promote the synthesis of a wide range of 3‐hydroxylactams through the catalytic monohydrogenation of phthalimides with total atom‐economy. It is important to remark that this transformation proceeds with full chemoselectivity, exhibiting complete tolerance to the presence of (hetero)aromatic rings and being able to stop the hydrogenation at the first intermediate level. A deep effort concerning material catalyst optimization and characterization study are performed, determining that the optimal [Ag/Al_2_O_3_] is composed of silver nanoparticles with an overall size of 2.3 nm homogeneously distributed across the alumina matrix. The characterization together with the performance of kinetic/mechanistic investigations allows to propose an intimate contact between accessible Ag^0^ sites and Lewis acidic centers is crucial for the catalyst system to perform the hydrogenative process with complete efficiency.

## Graphical abstract

3‐Hydroxylactams are obtained from phthalimides through a heterogeneous catalyzed chemoselective hydrogenation, able to stop the process at the first hydrogenation level. By using a rationally designed [Ag/Al_2_O_3_] recyclable solid nanocatalyst, a wide range of cyclic hemiamidals are accesed through a full atom‐economical reaction that preserves (hetero)aromatic rings for the first time.

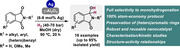

## 1 Introduction

Controlling chemoselectivity is one of the greatest challenges of current organic synthesis.^[^ ^1^ ^]^ Moreover, in the current context in which the development of sustainable processes is mandatory, chemoselectivity control should ideally be driven by a catalytic system. In this sense, the design of heterogeneous catalysts robust enough for being reused, and presenting multifunctional properties is highly desirable.^[^ ^2^, ^3^, ^4^, ^5^, ^6^ ^]^ Obviously, developing strategies that avoid the use of stoichiometric reagents and the generation of by‐products is also a key aspect.

**Scheme 1:**
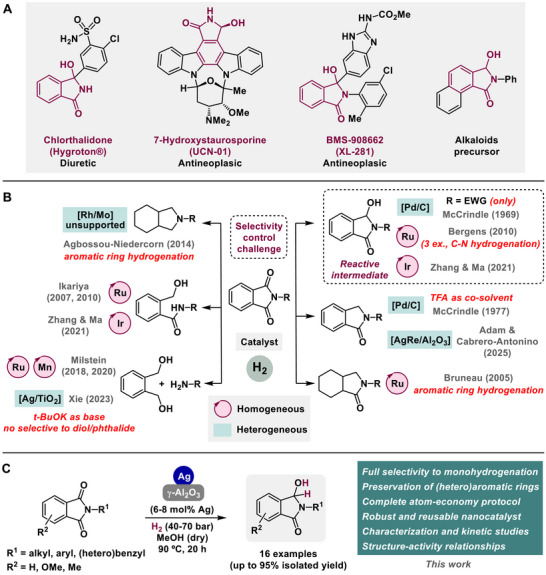
A) Examples of relevant molecules with biological activity or synthetic utility featuring 3‐hydroxyisoindolinone core. B) Catalytic hydrogenation of phthalimides to relevant products (general protocols). C) Selective monohydrogenation of phthalimides to 3‐hydroxyisoindolinones (this work). EWG = Electron‐withdrawing group. TFA = Trifluoroacetic acid.

3‐Hydroxyisoindolinones, also known as ω‐hydroxyisoindolinones, are pivotal scaffolds largely employed in organic synthesis as important building blocks for obtaining mainly heterocycles,^[^ ^7^, ^8^, ^9^, ^10^, ^11^, ^12^, ^13^, ^14^, ^15^, ^16^, ^17^, ^18^, ^19^, ^20^ ^]^ such as alkaloids,^[^ ^14^, ^15^, ^20^ ^]^ (**Scheme** **1**A), as well as functionalized amides or carbamates.^[^ ^8^, ^19^ ^]^ Their use as precursors for these relevant molecules is based on their ability to easily generate, under the appropriate conditions, *N*‐acyliminium ions, which are highly reactive intermediates. Moreover, this type of hydroxy‐containing lactams also presents a great interest for their structure, found in several compounds with important biological activities or, even, in molecules commercialized as drugs. Thus, as a significant example of this, chlorthalidone, commercialized as Hygroton®, is a diuretic drug used for the treatment of hypertension or other cardiovascular disorders (Scheme 1A).^[^ ^21^ ^]^ In addition, 7‐hydroxystauroporine (UCN‐01), a synthetic derivative of staurosporine, is a protein kinase inhibitor with antineoplastic activity,^[^ ^22^, ^23^, ^24^ ^]^ as well as BMS‐908662 (XL‐281) which is a RAF kinase inhibitor with utility for the treatment of melanoma and colorectal cancers (Scheme 1A).^[^ ^25^ ^]^

Consequently, during the last years, great efforts have been developed for designing approaches toward the synthesis of 3‐hydroxyisoindolinones. The most studied approximation for obtaining these compounds has been the direct reduction of phthalimides, as they are easily accessible starting materials, and their reduction to 3‐hydroxyisoindolinones is a straightforward protocol. Traditionally, the employment of (over)stoichiometric amounts of hazardous metal hydrides, such as LiAlH_4_ or NaBH_4_, or the use of metals (i.e., Zn) in strong acidic conditions, has been the most common protocols.^[^ ^26^, ^27^, ^28^, ^29^, ^30^, ^31^ ^]^ However, these procedures require a very strict control of reaction conditions to avoid the formation of undesired (over)reduction products, which, despite the efforts, are concomitantly formed in many cases. In addition, a not negligible amount of waste is generated after these processes.

More recently, metal‐ or base‐catalyzed homogeneous protocols making use of hydroxysiloxanes as chemoselective and mild reducing agents have been developed by Xie and coworkers for the production of 3‐hydroxyisoindolinones from phthalimides.^[^ ^32^, ^33^ ^]^ In these cases, the most important disadvantage is related to the use of an excess of the reducing agent and, hence, the consequent generation of waste products. In addition, elegant non‐catalytic reductive strategies based on electrochemistry have been independently developed in 2021 by the groups of Kawamata & Baran,^[^ ^34^ ^]^ Xiang^[^ ^35^ ^]^ and Wang^[^ ^36^ ^]^ to produce ω‐hydroxylactams from phthalimides. Unfortunately, the protocols require sophisticated experimental setups and (over)stoichiometric amounts of several organic additives. Moreover, in the example of Kawamata & Baran, overhydrogenated lactam products are afforded as the main compounds.^[^ ^34^ ^]^ Due to the large interest that 3‐hydroxyisoindolinones present, many other approaches have been recently developed for their synthesis,^[^ ^37^ ^]^ such as the benzylic C─H activation/oxidation of 2‐alkyl benzamides using photochemical,^[^ ^38^, ^39^ ^]^ radical,^[^ ^40^ ^]^ metal‐catalyzed^[^ ^41^ ^]^ or electrochemical methods.^[^ ^42^ ^]^

Therefore, from a practical and sustainable perspective, the synthesis of 3‐hydroxyisoindolinones through the catalytic chemoselective monohydrogenation of phthalimides is an appealing tool for the fine‐chemical industry. In this context, catalytic protocols efficient for performing the hydrogenation of carboxylic acid derivatives, such as imides, are highly interesting due to the large accessibility of these substrates and the great interest of the possible products formed.^[^ ^43^, ^44^, ^45^, ^46^, ^47^, ^48^ ^]^ However, these strategies are challenging because carboxylic acid derivatives are highly inactivated substrates toward hydrogenation, due to the low electrophilic character of their carbonyl group.^[^ ^49^ ^]^

In the specific case of phthalimides,^[^ ^50^ ^]^ the most common class of cyclic imides, the hydrogenation of these substrates is slightly more favored in comparison with other carboxylic acid derivatives due to the higher electrophilicity of their carbonyl groups.^[^ ^43^, ^46^, ^50^ ^]^ However, the difficulty in the hydrogenation of these compounds is more connected with the selectivity control. Phthalimides are synthetically enriched molecules whose hydrogenation can afford a wide range of different products, hence designing catalytic systems that can selectively drive the hydrogenation to a unique product is key (Scheme 1B).^[^ ^43^, ^46^, ^50^ ^]^ Among the possible hydrogenative pathways that are observed in the hydrogenation of phthalimides, it is important to highlight the C─O or C─N hydrogenation paths, which can proceed in one or both carbonyls, apart from the possible hydrogenation of the aromatic ring. During the last years, several groups ‐including us‐ have made remarkable efforts for the development of either homogeneous or heterogeneous catalytic hydrogenative protocols to selectively obtain a specific hydrogenation product from the corresponding phthalimide. Interestingly, isoindolinones, derived from the C═O hydrodeoxygenation of one carbonyl, have been obtained using homogeneous and heterogeneous systems.^[^ ^51^, ^52^, ^53^, ^54^ ^]^ Bruneau and co‐workers optimized a homogeneous system for synthesizing hexahydroisoindolinones, coming from a single C═O hydrodeoxygenation with concomitant reduction of aromatic fragment.^[^ ^55^ ^]^ On the other hand, the group of Agbossou–Niedercorn reported the double C═O hydrodeoxygenation/aromatic ring reduction to afford aliphatic cyclic amines.^[^ ^56^ ^]^

Moreover, strategies to obtain products coming from a C─N hydrogenolysis either in single or double versions, to obtain ω‐hydroxyamides^[^ ^57^, ^58^, ^59^ ^]^ or diols/phthalide and amines,^[^ ^60^, ^61^, ^62^, ^63^ ^]^ respectively, have also been developed. It is very interesting to note that all these possible reaction pathways, giving products derived from carbonyls hydrogenation, proceed through a common first tetrahedral intermediate that can be stabilized to afford valuable 3‐hydroxyisoindolinones. Up to date, three different catalytic examples have been reported for this last challenging process involving the synthesis of 3‐hydroxyisoindolinones by the chemoselective monohydrogenation of phthalimides.^[^ ^59^, ^64^, ^65^ ^]^ The first of them, reported by McCrindle and coworkers in 1969, makes use of pyrophoric [Pd/C] catalyst, and it was only active for phthalimides bearing electron‐withdrawing groups (EWG) in the nitrogen atom, showing a very narrow substrate scope.^[^ ^64^ ^]^ In 2010, the group of Bergens demonstrated that a Noyori‐type Ru‐homogeneous complex was capable of promoting the formation of three different 3‐hydroxyisoindolinones from the corresponding *N*‐substituted phthalimides at mild conditions.^[^ ^65^ ^]^ In this work, a slight increase in the reaction temperature, applied up to 60 °C, resulted in detrimental for product selectivity, detecting C─N hydrogenolysis products.^[^ ^65^ ^]^ Finally, Zhang, Ma, and coworkers reported in 2021 an expensive iridium homogeneous complex in combination with a ferrocene‐type ligand, able to promote the catalytic hydrogenation of a series of phthalimides to the desired 3‐hydroxylactams. In this work, the change of the employed ligand could totally drive the selectivity toward the ω‐hydroxyamide product coming from a C─N hydrogenolysis.^[^ ^59^ ^]^

Henceforth, the development of a recyclable, general, and efficient nanostructured catalyst for promoting the chemoselective monohydrogenation of phthalimides to 3‐hydroxyisoindolinones would constitute a substantial improvement in the field of carboxylic acid derivatives hydrogenative valorizations. Here, the challenges involve the design of a heterogeneous system able to preserve aromatic rings in hydrogenation conditions, active enough to hydrogenate an imide carbonyl group, but also with the proper selectivity to stop the hydrogenation sequence in the first step. In a previous contribution from our group, we designed a bimetallic silver‐rhenium alumina‐supported nanostructured material, namely [AgRe/Al_2_O_3_], which showcased a notable catalytic performance to promote the efficient formation of a wide range of lactams through a highly selective hydrodeoxygenation of the corresponding cyclic imides.^[^ ^51^, ^66^ ^]^ In the course of these studies, we investigated the important role of Ag in the first hydrogenation to the hemiamidal intermediate, while Ag‐Re cooperation was needed for the subsequent hydrogenative deoxygenation step. In addition, we elucidated Ag ability to preserve aromatic rings in hydrogenating conditions. Moreover, these studies also suggested the main role of acid centers present in alumina to promote the carbonyl activation toward hydride attack. Hence, as the starting hypothesis of this work, we propose the design of a silver‐based nanostructured material presenting the suitable physicochemical properties to mediate the selective monohydrogenation of phthalimides to the desired ω‐hydroxyisoindolinones with efficiency (Scheme 1C). To this end, we have designed, synthesized, characterized, and catalytically evaluated a series of nanomaterials based on Ag species over γ‐Al_2_O_3_ and we have been able to optimize an [Ag/Al_2_O_3_] system with the optimal properties for being an active and selective catalyst for the hydrogenation of a wide range of phthalimides to the corresponding ω‐hydroxyisoindolinones.

## 2 Results and Discussion

As previously commented, we decided to start this investigation focused on the design of an efficient and selective catalyst for the hydrogenation of phthalimides to 3‐hidroxyisoindolinones, by exploring the catalytic activity of an [Ag/Al_2_O_3_] nanomaterial. By analogy with a previous work in which we synthesized, characterized and optimized a [AgRe/Al_2_O_3_] nanocatalyst for obtaining isoindolinones from the corresponding phthalimides,^[^ ^51^, ^66^ ^]^ the [Ag/Al_2_O_3_] material was obtained after wet‐impregnation in acetone of 1 g of γ‐Al_2_O_3_ support with 0.4 mmol of [Ag(acac)] precursor, followed by a calcination step under air flow at 500 °C during 3 h with a heating ramp of 2 °C min^−1^. The wt.% of silver present at the material was determined by ICP‐AES to be 4.2 wt.% (Table S3, Supporting Information). In addition, X‐ray Power Diffraction (XRPD) analysis showed the presence of crystalline Ag^0^ nanoparticles with face‐centered cubic structure and crystallographic diffraction planes at 38°, 44°, 64°, 77° and 81° with Miller indexes of (111), (200), (220), (311) and (222), respectively (JCPDS No. 04–0783)^[^ ^67^, ^68^, ^69^ ^]^ (Figure S4, Supporting Information). By applying the Scherrer equation, a medium crystal size of 33 nm for Ag NPs was calculated in this material.

Once synthesized, the [Ag/Al_2_O_3_] material and with the aim of evaluating its catalytic activity, we chose the hydrogenation of *N*‐methylphthalimide **1** in the presence of 6 mol% of [Ag/Al_2_O_3_] nanomaterial and 50 mg of 4Å molecular sieves (MS), at 40 bar of H_2_ and 90 °C, during 14 h as a benchmark reaction. Initially, we became interested in comparing the effect of a variety of anhydrous solvents in the selective formation of hemiamidal **2** by the monohydrogenation of imide **1** (Table 1). The comparison between polar aprotic solvents such as cyclopentyl methyl ether (CPME), tetrahydrofuran (THF) or 1,4‐dioxane, with apolar, *n*‐heptane and toluene, and a polar protic solvent such as methanol, revealed that the best results were obtained using either *n*‐heptane or methanol (44% vs 77%, **Table** **1**, entries 4 and 6), being the results clearly superior in the case of MeOH. Notably, apart from the desired 3‐hydroxyisoindolinone **2**, no other compounds were detected in the reaction with MeOH, avoiding the formation of the C3‐alkoxylated isoindolinone coming from a reductive alkoxylation of **1** with methanol as a nucleophilic solvent.^[^ ^70^, ^71^ ^]^ Interestingly, in the absence of molecular sieves and employing the same reaction conditions, an enhanced yield of **2** up to 89% was detected with total selectivity (Table 1, entry 7). Therefore, from this point, the use of molecular sieves was discarded for all the catalytic experiments. Then, the reaction was essayed at the same conditions but employing a non‐anhydrous HPLC‐grade methanol as solvent, previously dried with 4Å MS for 3 h. In these conditions, an excellent yield of **2** of 92% was observed (Table 1, entry 8). On the contrary, the yield of **2** decreased to 85% when the same solvent was used without applying the predrying step (Table 1, entry 9). Other alcohol‐type non‐anhydrous HPLC‐grade solvents such as isopropanol or t‐amyl alcohol (dried with 4Å MS during 3 h prior to its use) were evaluated, albeit all of them afforded worse yields of **2** in comparison with methanol (Table 1, entries 10 and 11, 83 and 69%).

**Table 1:** Initial optimization conditions for the [Ag/Al_2_O_3_]‐catalyzed monohydrogenation of *N*‐methylphthalimide **1**.^a)^

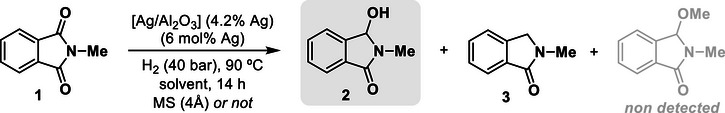					
Entry	Solvent	Conversion of 1 (%)^b)^	2 (%)^b)^	3 (%)^b)^	Selectivity to 2 (%)^b)^
1	CPME (anh.)	29	29	–	>99
2	THF (anh.)	20	18	–	90
3	1,4‐Dioxane (anh.)	14	13	–	93
4	*n*‐Heptane (anh.)	44	43	1	98
5	Toluene (anh.)	16	14	2	88
6	MeOH (anh.)	77	77	–	>99
7	MeOH (anh.)^c)^	89	89	–	>99
**8**	**MeOH** ^c)^, ^d)^	**92**	**92**	–	**>99**
9	MeOH^c)^, ^e)^	85	85	–	>99
10	*i*‐PrOH^c)^, ^d)^	83	83	–	>99
11	*t*‐AmylOH^c)^, ^d)^	69	69	–	>99
12	MeOH^c)^, ^d)^, ^f)^	66	66	–	>99
13	MeOH^c)^, ^d)^, ^g)^	75	75	–	>99
14	MeOH^c)^, ^d)^, ^h)^	65	65	–	>99
15	MeOH^c)^, ^d)^, ^i)^	<5	–	–	–
16	MeOH^c)^, ^d)^, ^j)^	97	91	7	94
17	MeOH^c)^, ^d)^, ^k)^	96	90	6	94
18	MeOH^c)^, ^d)^, ^l)^	97	90	7	93
19	MeOH^c)^, ^d)^, ^m)^	92	92	‐	>99

^a)^ Standard reaction conditions: *N*‐methylphthalimide **1** (0.25 mmol), [Ag/Al_2_O_3_] (4.2 wt.% Ag in the material, 6 mol% of Ag) calcined under air flow at 500 °C during 3 h with a heating ramp of 2 °C min^−1^, *n*‐dodecane (20 µL), 4Å Molecular Sieves (50 mg, predried at 300 °C under vacuum during 3 h), solvent (1 mL), H_2_ (40 bar) and 90 °C during 14 h.

^b)^ Conversion of **1**, yields of products **2** and **3,** and selectivity to product **2** were calculated by GC using *n*‐dodecane as internal standard.

^c)^ Reaction without 4Å MS.

^d)^ HPLC grade non‐anhydrous solvent, but predried with 4Å MS during 3 h.

^e)^ HPLC grade solvent.

^f)^ Run at 70 °C.

^g)^ Run with H_2_ (30 bar).

^h)^ Run with H_2_ (20 bar).

^i)^ Run with H_2_ (5 bar) and 70 °C.

^j)^ Run at 110 °C.

^k)^ Run with H_2_ (60 bar).

^l)^ Run with 8 mol% of Ag.

^m)^ Run for 20 h. MS = Molecular Sieves.

At this point, several reaction parameters, including temperature, hydrogen pressure, mol% Ag, and reaction time, were evaluated in the hydrogenation of cyclic imide **1** by using the optimal version of methanol as solvent (HPLC‐grade, dried with 4Å MS during 3 h prior use). A decrease in reaction temperature and hydrogen pressure, up to 70 °C and 30 or 20 bar, resulted in detrimental for the catalytic activity, although selectivity to **2** was maintained (Table 1, entries 12–14). As it was expected, a simultaneous decrease in temperature and H_2_ pressure (70 °C and 5 bar) afforded a negligible conversion (Table 1, entry 15). On the other hand, an increase of these parameters, up to 110 °C or 60 bar, improved phthalimide **1** conversion but, unfortunately, diminished **2** selectivity, and undesired lactam **3** was detected in 6–7% yield (Table 1, entries 16 and 17). Finally, the effect of the variation in Ag mol% or reaction time was also studied, and the reaction was tested at the optimized reaction conditions but either employing 8 mol% of silver or during 20 h of reaction time (Table 1, entries 18 and 19). In the case of the reaction with 8 mol% % of Ag, more conversion of imide **1** was observed but with a concomitant decrease in selectivity (Table 1, entry 18), whereas for the experiment run for 20 h, no significant differences were found (Table 1, entry 19). Based on all these experiments, it was established that [Ag/Al_2_O_3_] nanomaterial (with a 4.2 wt.% of Ag) constitutes an effective catalyst for promoting synthesis of **2** from **1** affording very good yields of hemiamidal **2** (92% yield) with excellent selectivities at 40 bar of H_2_, 90 °C, during 14 h and in the presence of methanol as a solvent.

**Figure 1:**
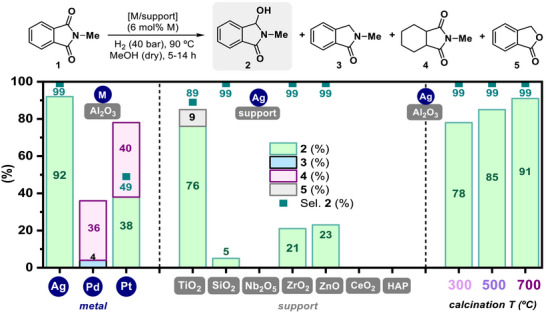
Influence of metal and support nature, and calcination temperature applied in the material synthesis, in the catalytic activity for the hydrogenation of **1**. [M/support] materials were prepared by wet‐impregnation of 1 g of selected support with 0.4 mmol of metal precursor, followed by calcination at 500 °C during 3 h. For [Ag/Al_2_O_3_] system, additional calcination temperatures (from 300 to 700 °C) were employed. Metal wt.% content of nanomaterials was determined by ICP‐AES. Standard reaction conditions: *N*‐methylphthalimide **1** (0.25 mmol), [M/support] (6 mol% of M, considering the wt.% metal measured by ICP‐AES), *n*‐dodecane (20 µL), MeOH (1 mL, predried with 4 Å MS), H_2_ (40 bar) at 90 °C during 14 h (for metal and support study) or 5 h (for calcination temperature study). Yields of products **2**, **3**, **4,** and **5**, and selectivity to product **2** were calculated by GC using *n*‐dodecane as an internal standard.

Next, we became interested in determining if Ag species and γ‐Al_2_O_3_ matrix are both essential components of the catalytic system. With this aim, we synthesized, characterized, and evaluated the catalytic performance of several nanostructured [M/support] systems in the hydrogenation of **1** (**Figure** **1**; Table S1 and Figures S4 and S5, Supporting Information). Several [M/Al_2_O_3_] (M = Pd, Pt, Fe, Co, Ni and Cu) and [Ag/support] materials (support = SiO_2_, TiO_2_, ZrO_2_, Nb_2_O_5_, ZnO, CeO_2_ and HAP) were prepared following the same procedure as the indicated before for [Ag/Al_2_O_3_], but modifying the metal precursor (0.4 mmol) or the matrix support (1 g) (see section 2.2 of the Supporting Information for more specific details). The wt.% metal for each material was determined by ICP‐AES (see Tables S3 and S4, Supporting Information). In addition, X‐ray Power Diffraction (XRPD) analysis of the different [M/Al_2_O_3_] and [Ag/support] materials was registered (Figures S4 and S5, Supporting Information). With regard to [M/Al_2_O_3_] materials, only in the case of [Ag/Al_2_O_3_], the presence of crystalline Ag^0^ NPs was observed. The other [M/Al_2_O_3_] materials did not show other crystallographic diffraction signals, apart from the ones from alumina support (Figure S4, Supporting Information). On the other hand, [Ag/support] materials showed the presence of crystalline Ag^0^ nanoparticles in the case of [Ag/ZrO_2_], [Ag/Nb_2_O_5_], [Ag/ZnO], [Ag/CeO_2_] and [Ag/HAP] systems, with a medium Ag NPs crystal size of 29, 55, 35, 38 and 49 nm, respectively (Figure S5, Supporting Information). Characteristic Ag diffraction peaks were not detected for [Ag/SiO_2_] and [Ag/TiO_2_] materials (Figure S5, Supporting Information).

Then, we tested the catalytic activity of [M/Al_2_O_3_] materials (M = Pd, Pt, Fe, Co, Ni, and Cu) at the optimized reaction conditions (6 mol% of M, 40 bar of H_2_, 90 °C during 14 h). In comparison with the Ag nanomaterial, [Pd/Al_2_O_3_] and [Pt/Al_2_O_3_] exhibited certain catalytical activity, but with a very notable lack of selectivity toward the formation of hemiamidal **2**. Indeed, product **2** formation was only observed in moderate yields in the case of the Pt‐based system (38% yield). In fact, both systems are more prone to form aromatic‐ring hydrogenated imide product **4**, in which no carbonyl group reduction occurred (Figure 1, left). On the other hand, non‐noble based solid [M/Al_2_O_3_] materials (M = Fe, Co, Ni, and Cu) afforded no conversion of **1** under the reaction conditions tested (Table S1, Supporting Information). The catalytic activity of the [Ag/support] materials, using TiO_2_, SiO_2_, Nb_2_O_5_, ZrO_2_, ZnO, CeO_2_ and HAP as solid matrix with different acid‐base properties, was evaluated for the hydrogenation of imide **1** (Figure 1, middle). Among all the materials tested, only [Ag/TiO_2_] gave similar results than [Ag/Al_2_O_3_] nanocatalyst, but showing less activity (76% yield of product **2** vs 92% with [Ag/Al_2_O_3_]) and selectivity to **2** (89% of selectivity to **2** in comparison with the full selectivity yielded by the optimal [Ag/Al_2_O_3_] catalyst). In the presence of [Ag/TiO_2_], phthalide **5**, coming from a C─N hydrogenolysis of the imide followed by cyclization, was also detected as a byproduct in a 9% yield. Other silver‐supported materials tested were completely inactive or gave very low yields of **2** (5, 21, and 23% in the cases of [Ag/SiO_2_], [Ag/ZrO_2_], and [Ag/ZnO] systems, respectively) (Figure 1, middle).

Once demonstrated that [Ag/Al_2_O_3_] system is composed of the most optimal metal‐support combination, we decided to investigate if the calcination temperature applied in the synthesis of the material had an influence in the catalytic behavior (Figure 1, right and Table S2, Supporting Information). Therefore, apart from the material calcined at 500 °C, four additional calcination temperatures (300, 400, 600, and 700 °C) were employed in the synthesis of [Ag/Al_2_O_3_], maintaining constant the rest of the parameters. Catalytic evaluation of the different versions of the material for imide **1** hydrogenation was performed at 6 mol% of Ag, 40 bar of H_2_ and 90 °C, during 5 h. Interestingly, these experiments showed that there is a direct relationship between the calcination temperature and the catalytical activity, being the [Ag/Al_2_O_3_] system calcined at 700 °C the one affording **2** in the highest yields (91%, Figure 1, right and Table S2, Supporting Information, entry 4), while the material calcined at 300 °C showcased a decreased activity (78% of **2**, Figure 1, right and Table S2, Supporting Information, entry 1) and the one calcined at 500 °C afforded an intermediate activity (85% of **2**, Figure 1, right and Table S2, Supporting Information, entry 3). In view of these results, the material calcined at 700 °C was selected as the optimal one to continue our studies.

**Figure 2:**
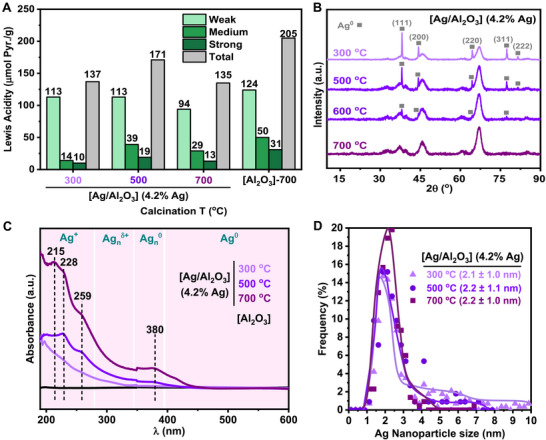
Characterization of [Ag/Al_2_O_3_] nanomaterials calcined at different temperatures: A) Density and strength of Lewis acid sites determined by Pyr‐FTIR, B) XRPD patterns, C) UV–vis DR spectra, and D) Average Ag nanoparticle size (from HAADF‐HRSTEM analysis). γ‐Al_2_O_3_ (calcined or not) has been used in some cases for comparison. It is detected the presence of crystalline Ag^0^ NPs with face‐centered cubic structure and crystallographic diffraction planes with Miller indexes of (111), (200), (220), (311) and (222) (JCPDS No. 04–0783).^[^ ^67^, ^68^, ^69^ ^]^

With the aim of better understanding these results and searching for possible structure‐activity relationships, we decided to perform a characterization of the structural and physicochemical properties of selected [Ag/Al_2_O_3_]‐based materials calcined at different temperatures (**Figure** **2**). In addition, silver‐free γ‐Al_2_O_3_ support, was also included in this study for comparison.

First, we decided to evaluate several properties of the nanocatalysts, such as the Lewis acidity, employing pyridine‐FTIR spectroscopy analysis (Figure 2A), and the specific surface area, using N_2_ adsorption isotherms (Table S6, Supporting Information). Both physicochemical parameters are of interest considering that surface species are the ones with the largest influence in catalytical activity. It is important to note that in this transformation, a key step for activating imide carbonyl toward hydrogenation involves the direct interaction between the Lewis acid centers present at the surface of the material, Al^3+^ atoms in our case, and the poorly electrophilic C═O group of the imide.

Therefore, in principle, we could expect that the [Ag/Al_2_O_3_] material with the highest density of surface Lewis acid centers, would exhibit the best catalyst performance. Moreover, it is known that alumina calcined at higher temperatures typically shows a higher amount of surface Lewis acid centers, since more degree of dehydroxylation at the surface takes place.^[^ ^72^ ^]^ The determination of the density and strength nature of the surface Lewis acid sites of several [Ag/Al_2_O_3_]‐based materials with a 4.2% Ag and calcined at 300, 500, and 700 °C was performed in comparison with the corresponding alumina support also calcined at 700 °C. This information was inferred from the integration of the characteristic pyridine‐Lewis acid adduct IR band (µmol of pyridine/gram of material) after its desorption at different temperatures (150 °C = weak sites; 250 °C = medium sites; 350 °C = strong sites). As can be observed in Figure 2A, in general, the three [Ag/Al_2_O_3_] nanomaterials showed a lower amount of acid sites than the calcined support, which can be interpreted as Ag centers covering γ‐Al_2_O_3_ acid sites. However, no correlation between calcination temperature and the amount of acid sites could be established. In fact, the material calcined at 700 °C and with the highest catalytical activity, was the one presenting the lower density of total acid sites, while the material calcined at an intermediate temperature of 500 °C, and with an intermediate catalytical activity, was the one showing the highest density of total as well as weak, medium and strong acid sites (Figure 2A).

Regarding specific surface area and average pore diameters, these properties were calculated through N_2_ adsorption isotherms by BET and BJH methods for [Ag/Al_2_O_3_]‐based materials with a 4.2% Ag and calcined at 300, 500 and 700 °C, as well as the alumina support also calcined at 500 °C (Table S6, Supporting Information). In general, all the materials showed lower surface areas and pore diameters than the calcined matrix due to the presence of Ag species. A comparison between the materials did not afford significant conclusions as surface areas and pore diameter values did not show significant variations (from 168 to 175 m^2^/g and from 105 to 110 Å).

Interestingly, X‐ray Powder Diffraction Analysis of the [Ag/Al_2_O_3_] (4.2% Ag) materials calcined at 300, 500, 600, and 700 °C showed important differences (Figure 2B). In the case of the calcined materials at 300, 500, and 600 °C, the existence of crystalline Ag^0^ nanoparticles with face‐centered cubic structure and crystallographic diffraction planes at 38°, 44°, 64°, 77° and 81° with the corresponding denoted Miller indexes were detected,^[^ ^67^, ^68^, ^69^ ^]^ apart from Al_2_O_3_ phases. However, crystallographic diffraction planes attributable to Ag^0^ NPs were not detected for the material calcined at 700 °C, showing just signals corresponding to alumina support. This observation suggests the existence of silver nanoparticles with an overall size below 5 nm and, hence, with a higher degree of dispersion across the support in this material. In fact, a medium crystal size of 44 and 33 nm could be calculated for Ag^0^ nanoparticles in materials calcined at 300 and 500 °C, respectively, while in the case of material calcined at 600 °C, signals were too broad for calculating crystal size. It is interesting to note that an increase in calcination temperature commonly drives to a higher silver nanoparticle size,^[^ ^73^ ^]^ but here the opposite effect was observed. All these results made us to consider that the remarkable catalytic performance displayed by [Ag/Al_2_O_3_] (4.2% Ag) calcined at 700 °C could be related with a higher density of silver metallic centers optimally dispersed along the alumina matrix. In addition, this effect would also explain the existence of a minor amount of accessible Lewis acid centers in this material.

UV–vis Diffuse Reflectance Spectroscopy (UV–vis DRS) serves as a practical tool to elucidate the oxidation state and degree of dispersion of silver surface species present in a solid material. Indeed, in the literature, between three and four types of Ag surface species have been identified, depending on the wavelength number (λ) corresponding to the absorbance at the UV–vis spectra.^[^ ^74^, ^75^, ^76^ ^]^ The absorbance in the region below 250 nm has been assigned to cationic Ag^+^ species, whereas maximums of absorption between 250 and 390 nm, have been ascribed to Ag_n_^δ+^ clusters (n ≤ 8) with different sizes and oxidation states. Finally, the absorption above 390 nm has been explained to be caused by superficial plasmonic resonance absorption characteristic of Ag^0^ NPs. It is interesting to remark that some authors have differentiated in the Ag^0^ region two kinds of species: Ag_n_^0^ clusters with a higher degree of dispersion at λ between 350 and 400 nm and silver nanoparticles of bigger size at λ >400 nm.^[^ ^77^ ^]^

Figure 2C shows the UV–vis DRS spectra of [Ag/Al_2_O_3_] (4.2% Ag) materials freshly calcined at 300, 500, and 700 °C, together with the non‐calcined alumina support, which does not show any signal. Remarkably, the three materials presented absorbance at wavelengths between 200 and 420 nm, which can be assigned to the presence of Ag^+^, Ag_n_^δ+^ and Ag_n_^0^ surface species, while characteristic signals of Ag^0^ nanoparticles were not observed. A direct relationship between the absorbance and the calcination temperature was observed, being the material calcined at 700 °C the one displaying higher absorbances, especially at the Ag^+^ region. In fact, in the case of the materials calcined at 700 and 500 °C it was possible to identify maximum absorption peaks at 215, 228, 259, and 380 nm, while the material calcined at 300 °C showed an almost negligible band between 200 and 350 nm (Figure 2C). It is important to note that this technique offers complementary information to the one obtained in DRX, as both techniques show a different degree of penetration in the samples (10–20 nm for UV‐DRS and 1–5 µm for XRPD analysis).^[^ ^78^ ^]^ Hence, considering this, we can interpret from the information of both techniques that these materials are composed of Ag^0^ nanoparticles covered by silver cluster species with different oxidation states. In addition, both DRX and UV–vis DRS techniques indicate that Ag^0^ NPs decrease in size linearly with the increment in calcination temperature employed in the material synthesis.

At this point, we were interested in obtaining more information about the structural and morphological nature of the alumina‐supported silver species present in the materials calcined at 300, 500, and 700 °C. Hence, we studied these materials by high‐angle annular dark‐field high‐resolution scanning transmission electron microscopy (HAADF‐HRSTEM) technique (Figure 2D; Figure S7, Supporting Information). In the three samples measured, silver nanoaggregates between 1 and 10 nm were detected, and the average silver nanoparticle size was of ≈2.1 nm (Figure S7, Supporting Information). However, a deeper analysis of the detected silver nanoparticles with a size >4 nm allowed us to correlate that an increment in calcination temperature drives to a narrow Ag nanoparticle size distribution, being the material calcined at 700 °C the one which shows the smallest number of nanoparticles with sizes larger than >4 nm (Figure 2D; Figure S7, Supporting Information).

Therefore, [Ag/Al_2_O_3_] (4.2 wt.% Ag) system calcined at 700 °C, constitutes a nanostructured material composed by highly dispersed silver nanoaggregates of <4 nm (average size ≈2.2 nm) that are covered by Ag^+^ species and presenting a limited number of Lewis acid sites. The fact that this nanocatalyst presents a superior catalytic activity than other similar materials with larger Ag^0^ nanoparticles, although with a higher amount of acid sites, suggests that the presence of a large amount of Ag active sites is key for the monohydrogenation of **1** to **2**. However, if a material with a large amount of Ag active centers and simultaneously presenting a higher density of Lewis acid sites was synthesized, imide carbonyl activation should be improved and, hence, also the corresponding catalytic performance.

In this direction, we decided to catalytically evaluate in the monohydrogenation of **1** to **2** a series of newly [Ag/Al_2_O_3_] synthesized materials obtained by calcination at 700 °C and presenting a variety of silver loadings (wt.%) between 1 and 20 wt.% (for more information on the synthesis procedure, see section 2.2 of Supporting Information). Thus, the corresponding [Ag/Al_2_O_3_] materials were obtained and their wt.% Ag was measured by ICP‐AES, resulting of 1.1, 2.4, 8.9, 12.5, 16, and 20.3 (see Table S5, Supporting Information). Next, its catalytic evaluation in the hydrogenation of **1** at 20 bar of H_2_, 90 °C, employing methanol (HPLC‐grade, dried with 4Å MS during 3 h prior use) as solvent and maintaining a constant amount of 6 mol% Ag versus imide **1** was conducted (Figure S1, Supporting Information). After 16 h of reaction time, the 12.5 wt.% alumina‐supported Ag material was the one affording the best yields (91% of **2**). However, the differences exhibited in the catalytic activity from the diverse solid silver‐supported systems were not too significant, and, with the aim of confirming this result, the nanocatalysts were compared at shorter reaction times (4 h). In these reaction conditions, such differences in the yield obtained for **2** were slightly larger, establishing [Ag/Al_2_O_3_] (12.5 wt.% Ag) material as the best catalyst for the monohydrogenation of **1** to **2** (54% of **2**). For materials with silver metal loadings below or above 12.5 wt.%, hemiamidal **2** yield decreased with respect to the maximum reached with 12.5 wt.% Ag nanomaterial (Figure S1, Supporting Information). In all cases, hemiamidal **2** was the only product detected, showing a total selectivity toward **1** monohydrogenation in the presence of the evaluated silver nanostructured systems.

**Figure 3:**
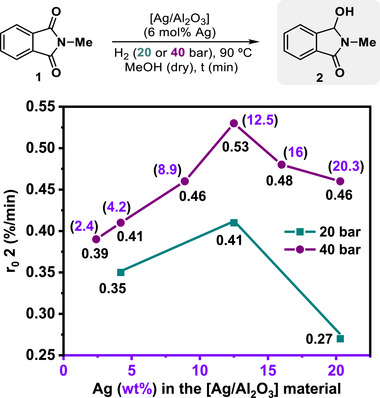
Influence of Ag wt.% at the [Ag/Al_2_O_3_] nanomaterial in the initial rate of product **2** formation (r_0_ **2**) from the catalytic monohydrogenation of **1**. The materials were prepared by wet impregnation of γ‐Al_2_O_3_ (1 g) with the desired [Ag(acac)] amount in acetone, dried, and then calcined at 700 °C during 3 h. The real Ag (wt.%) amount was determined by ICP‐AES. Standard reaction conditions: *N*‐methylphthalimide **1** (0.75 mmol), [Ag/Al_2_O_3_] (2.4–20.3% of Ag in the material, 6 mol% of Ag), *n*‐dodecane (60 µL), 3 mL of MeOH (previously dried with 4 Å MS), 40 (purple circles) or 20 (green squares) bar of H_2_ at 90 °C. Initial rate values were calculated from the slope of the linear equation: [yield **2** (%) = r_0_ × t (min)] defined at initial reaction times and expressed as [r_0_ **2** (%/min)] and determined by GC using *n*‐dodecane as internal standard.

To further confirm the higher efficiency of the [Ag/Al_2_O_3_] (12.5 wt.% Ag) material in comparison with the other systems, we performed kinetic studies for the hydrogenation of **1** at 90 °C using 6 mol% Ag of catalyst, either under 40 or 20 bar of H_2_ (**Figure** **3**). Initial rate values calculated for product **2** formation (r_0_ **2**, expressed as %/min) at 40 bar of H_2_ served us to corroborate that an increase of wt.% Ag at the material used as catalyst from 2.4% up to 12.5% showed a direct relationship with the r_0_ **2** value (from 0.39 to 0.53%/min, Figure 3, purple circles). In the case of materials composed by silver loadings >12.5% (as 16 or 20.3 wt.%), a decrease in the initial rate for compound **2** formation was observed. Similarly, kinetic experiments performed at milder reaction conditions (20 bar of H_2_ instead of 40) comparing three selected [Ag/Al_2_O_3_] materials (with 4.2, 12.5, or 20.3 wt.% Ag) showed even more notable differences between the catalytic efficiencies of the optimal 12.5 wt.% Ag system and the others (Figure 3, green squares, 0.35, 0.41, and 0.27%/min, respectively).

**Figure 4:**
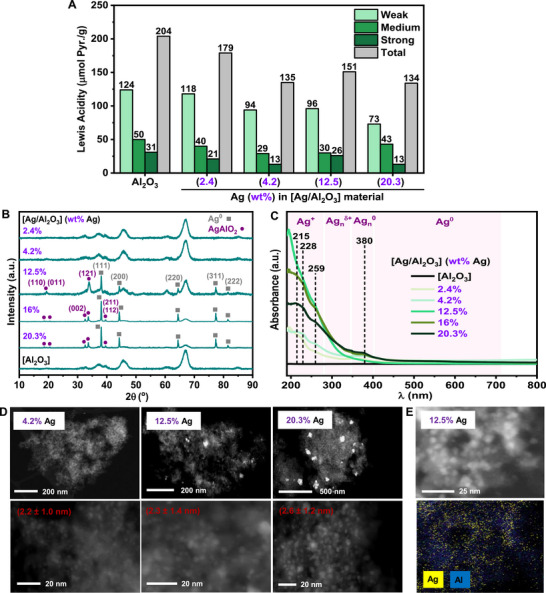
Characterization of [Ag/Al_2_O_3_] materials with different Ag wt.%: A) Density and strength of the Lewis acid sites determined by Pyr‐FTIR, B) XRPD patterns, C) UV–vis DR spectra, and D) Analysis of the surface aggregation and the average of silver nanoparticle size (from HAADF‐HRSTEM) and E) STEM‐XEDS analysis. It is detected the presence of crystalline Ag^0^ NPs with face‐centered cubic structure and crystallographic diffraction planes with Miller indexes of (111), (200), (220), (311) and (222) (gray squares, JCPDS No. 04–0783) ^[^ ^67^, ^68^, ^69^ ^]^ and the presence of AgAlO_2_ species with dela‐fossite structure and crystallographic diffraction planes with Miller indexes of (011), (110), (121), (002), (211) and (112) (purple circles, JCPDS No. 21–1070).^[^ ^79^ ^]^

Once demonstrated that silver metal loading present at the [Ag/Al_2_O_3_] nanomaterial calcined at 700 °C constituted a relevant factor in the catalytic efficiency, we became very interested in conducting a deep characterization study of selected [Ag/Al_2_O_3_] solids with a variety of Ag loadings. Considering the previously observed relationship between Ag dispersion and catalytic activity, it was highly interesting to investigate this aspect as well as other features such as acidity and surface. Thus, Pyr‐FTIR, H_2_‐Temperature Programmed Reduction (TPR), XRPD, UV–vis DRS, and HAAADF‐HRSTEM measurements and X‐ray Energy Dispersive Spectroscopy analysis were the employed techniques to investigate the materials' structure and chemical nature (**Figure** **4**; Figures S8–S10 and S16, Supporting Information).

First, Pyr‐FTIR spectroscopy analysis of [Ag/Al_2_O_3_] materials containing 2.4, 4.2, 12.5, and 20.3 wt.% Ag, as well as the alumina support calcined under air flow at 700 °C was performed (Figure 4A). As it was expected, a clear difference between the Lewis acid sites quantified in the support and in the different materials was observed, confirming the capacity of silver to cover Lewis acid sites of the support, probably because of an Al‐O‐Ag interaction. In fact, for materials with wt.% Ag of 2.4, 4.2, and 20.3, the amount of Lewis acid sites diminished at higher amounts of silver. Interestingly, an exception in this behavior was observed in the case of the optimal [Ag/Al_2_O_3_] (12.5 wt.% Ag) material in which the amount of either total, weak, medium, and strong acid Lewis acid sites was superior to that of materials either charged at 4.2 and 20.3 wt.% Ag (Figure 4A). This improved amount of Lewis acid sites in [Ag/Al_2_O_3_] (12.5 wt.% Ag) could explain, at least in part, the superior catalytic activity of this material, considering that acid centers are key for activating the imide carbonyl group toward its hydrogenation.

Specific surface area and average pore diameters calculated through N_2_ adsorption isotherms by BET and BJH methods were measured and compared for [Ag/Al_2_O_3_]‐based materials calcined at 700 °C with a 4.2% and 12.5 wt.% Ag. As it could be expected, the surface area of the most Ag charged material resulted smaller in comparison with 4.2% wt.% Ag (152.5 m^2^/g vs 168.01 m^2^/g).

To further understand the H_2_ activation capacity of these materials and know more about the dispersion of Ag particles present in them, TPR‐H_2_ analysis of [Ag/Al_2_O_3_] (4.2% Ag) and [Ag/Al_2_O_3_] (12.5% Ag) materials was performed (Figure S16, Supporting Information). Both nanocatalysts showed several peaks ascribable to H_2_ consumption by oxidized silver species (Figure S16, Supporting Information). More specifically, hydrogen consumption signals at 76 and 117 °C were detected in the case of the 4.2% Ag material, usually related to the surface oxygen reduction onto silver centers or Ag_2_O clusters of a relatively big size.^[^ ^77^, ^80^ ^]^ On the other hand, for [Ag/Al_2_O_3_] (12.5% Ag) system, a narrowed signal at 80 °C was detected together with a broader signal at 300 °C. This peak at higher temperatures is commonly attributed to Ag_2_O small, well‐defined clusters with a strong interaction with the support.^[^ ^77^, ^80^ ^]^ Therefore, from this technique, we can infer that both materials present different degrees of Ag species dispersion.

X‐Ray Powder Diffraction Analysis of [Ag/Al_2_O_3_] (2.4, 4.2, 12.5, 16, and 20.3 wt.% Ag) materials showed that, for materials with silver loadings below 5%, only diffraction peaks belonging to alumina support were observed, indicating a good dispersion of the supported silver entities (Figure 4B). However, in the case of silver‐alumina materials with 12.5, 16, and 20.3 wt.% metal loadings, diffraction peaks corresponding to crystallographic planes (111), (200), (220), (311) y (222) of Ag^0^ NPs were detected (Figure 4B, gray squares).^[^ ^67^, ^68^, ^69^ ^]^ By application of the Scherrer equation, an overall size of 34, 47, and 48 nm for visible Ag^0^ nanoparticles by DRX could be calculated for 12.5, 16, and 20.3 wt.% Ag systems, respectively. Noteworthy, crystallographic diffraction peaks corresponding to AgAlO_2_ species with Miller indexes of (011), (110), (002), (121), (211) y (112) and *dela*‐fossite structure (JCPDS 21–1070) were also observed in these cases (Figure 4B, purple circles).^[^ ^79^ ^]^ Formation of AgAlO_2_ species has been described for materials containing silver loadings above 5% onto an alumina matrix. Some authors ascribe the existence of these crystalline AgAlO_2_ species to the presence of highly dispersed silver entities across the alumina surface, which allow the anchoring of the silver metallic species through Ag‐O‐Al interactions.^[^ ^81^, ^82^ ^]^

Considering the confirmed presence of Ag^0^ nanoparticles at the [Ag/Al_2_O_3_] (12.5 wt.% Ag) optimal nanocatalyst, it was interesting to analyze the different materials calcined at 700 °C (2.4, 4.2, 12.5, 16 and 20.3 wt.% Ag) by UV–vis Diffuse Reflectance Spectroscopy, in order to elucidate the oxidation state of surface Ag species (Figure 4C). All the materials absorbed at wavelengths between 200 and 400 nm, with identical maximums at λ values of 215, 228, 259, and 380 nm (Figure 4B, see also Figure 2C). This suggests the coexistence of surface atomic entities of Ag^+^ and Ag_n_^δ+^ and Ag_n_° clusters with diverse size and oxidation state as surface species and discards the presence of light‐accessible Ag^0^ NPs in the surface of all the materials. It is very interesting to quantitatively analyze the absorbance magnitudes, especially in the Ag^+^ region, where they follow for the different materials the same trend as catalytic activity, being the material charged at 12.5 wt.% Ag the one showing the highest absorbance magnitudes. [Ag/Al_2_O_3_] nanocatalysts charged at less than 12.5 wt.% Ag (2.4 and 4.2 wt.% Ag) showed an increasing absorbance tendency proportional to Ag charge, while the ones with larger Ag loadings (16 and 20.3 wt.% Ag) showed a decreasing absorbance tendency proportional to Ag charge. This observation indicates that the optimal material has the ideal amount of silver to maximize the presence of Ag^+^ surface species, probably covering Ag^0^ nanoparticles. It is plausible to propose that materials with higher Ag loadings present major aggregation, while materials with a lower Ag loading, despite avoiding the presence of DRX visible Ag^0^ nanoparticles, present a minor amount of accessible Ag^+^ centers.

In order to confirm these observations through a morphological study, HAADF‐HRSTEM images of [Ag/Al_2_O_3_] (1.1, 2.4, 4.2, 12.5, 16, and 20.3 wt.% Ag) materials were registered and are depicted in Figure 4D and Figures S8 and S9 (Supporting Information). Images at low magnification showed a great dispersion of the metallic entities for systems containing lower silver loadings, such as [Ag/Al_2_O_3_] at 1.1, 2.4, and 4.2 wt.% Ag, while for samples with a higher silver content like [Ag/Al_2_O_3_] at 12.5, 16, and 20.3 wt.% Ag, silver metallic nanoparticles of bigger size were observed (see Figure 4D; Figure S8, Supporting Information). Indeed, the presence of these large particles was proportionally increased with the increment of the silver loading. For [Ag/Al_2_O_3_] charged at 16 and 20.3 wt.% Ag, the existence of these large metallic aggregates was more notable in comparison with the optimal material composed of 12.5% Ag (see Figure 4D and Figure S8, Supporting Information). These observations were in clear agreement with the results obtained by XRPD analysis of the samples. However, HAADF‐HRSTEM images taken at high magnification revealed the existence of a huge amount of small silver nanoparticles, with a very similar size, in all the materials (Figure 4C; Figure S9, Supporting Information). Histogram particle size analysis of more than 200 nanoparticles indicated an overall silver nanoparticle size ≈2.1–2.3 nm for the samples studied, being 2.6 nm in the case of the 20.3 wt.% Ag system (Figure S10, Supporting Information). X‐Ray Energy Dispersive Spectroscopy (XEDS) analysis of the optimal silver material [Ag/Al_2_O_3_] (12.5 wt.% Ag) confirmed an excellent distribution of the silver metallic entities across the alumina matrix support (Figure 4E).

With all these experimental data, we could establish that monohydrogenation of **1** to **2** is mainly influenced by the following factors: 1) H_2_ dissociation promoted by exposed Ag metallic centers at the surface material, and 2) the increment of the electrophilic character of C═O group through interaction between **1** and the Lewis acid centers from the support (Al^3+^). In addition, it should be remarked that a suitable spatial proximity between the two kinds of active centers can play a key role, since the monohydrogenation could be occurring in a synergistic manner. Among all the materials tested, [Ag/Al_2_O_3_] (12.5 wt.% Ag) was the one that showed the best catalytic performance in the formation of **2** from **1** monohydrogenation. Regarding Lewis acidity, for materials composed of lower wt.% Ag it was found an inverse tendency between its surface Lewis acidity and the catalytic properties displayed, which would suggest that Lewis acidity of the material does not represent the most important factor in the design of a suitable catalyst for this reaction. Therefore, the activation/dissociation of molecular hydrogen process onto the catalyst surface would represent a more determining step for the overall process. Indeed, the optimal nanocatalyst, [Ag/Al_2_O_3_] (12.5 wt.% Ag) has clearly shown by UV–vis DRS to be the one presenting the largest amount of Ag centers on the surface of the material. Moreover, this material has also been shown to present a relatively high number of Lewis acid sites, which would reinforce the synergistic concept between Ag active sites for H_2_ activation and Lewis acid sites increasing carbonyl electrophilic character. Materials with a lower Ag wt.%, such as 2.4%, despite having more acid centers, present less accessible Ag species and, therefore, the synergy between both centers should be less efficient. On the other hand, nanomaterials with higher Ag wt.%, such as 20.3%, present more aggregation and, also, less Lewis acid centers.

At this stage, we were interested in demonstrating the heterogeneous nature of the optimal [Ag/Al_2_O_3_] (12.5% Ag) nanocatalyst and evaluating its potential reusability toward successive reaction cycles. With this aim, we performed a yield/time kinetic profile for the hydrogenation of **1** under standard reaction conditions (40 bar of H_2_, 90 °C and 6 mol% Ag, see Figure S2, Supporting Information). In parallel, we performed the same kinetic experiment but performing the filtration of the solid catalyst after 30 min of the reaction start, being the autoclave previously cooled and depressurized. Then, the reaction mixture was again introduced in another autoclave and left up to complete a total time of 5 h. As Figure S2 (Supporting Information) displays, in the absence of the solid nanocatalyst, conversion of **1** and yield of **2** were maintained at the same values as after 30 min of usual reaction conditions, both of them remaining constant ≈20%. In contrast, the kinetic curve detected for the reaction mixture containing the catalyst reached >90% of **1** conversion and yield of **2**. In addition to filtration tests, the detection of a possible metal lixiviation from the solid catalyst employed was performed. With this purpose, the material recovered after a first cycle of reaction was measured by ICP‐AES, detecting a 12.2% of silver in the solid. A negligible 0.12% of the initial silver amount present in the catalyst was found in the reaction mixture using the same technique. Elemental analysis of the freshly recovered material showed a 2.1% carbon content, coming from organic compound residue. All these results confirm the heterogeneous nature of [Ag/Al_2_O_3_] catalyst system and discard the lixiviation of metal species under the reaction conditions employed.

To our delight, reusability studies of the optimal catalytic system for the monohydrogenation of **1** to **2** showed good results (Figure S3, Supporting Information). [Ag/Al_2_O_3_] catalyst system could be employed until five successive reaction cycles, just showing a slight decrease in the catalytic efficiency at the 4^th^ and 5^th^ cycle (from 91% yield of **2** at 1^st^ cycle up to 84 and 78% of **2** at 4^th^ and 5^th^ cycle, respectively) but maintaining intact the selectivity toward hemiamidal product **2**. It should be mentioned that in order to activate the solid nanocatalyst between each successive reaction cycle, after each use, the material was washed with EtOAc and acetone and, then, calcined at 300 °C under air flow during 3 h (heating ramp of 2 °C min^−1^). This treatment of the material before each successive use serves to abolish the organic residues present at the recovered solid and is essential for maintaining the catalytic activity of the system across the successive reaction cycles.^[^ ^51^ ^]^

Thereafter, we wanted to evaluate the effect that the application of the reaction conditions had on the structural and physicochemical properties of the silver solid nanocatalyst. XRPD analysis of the recovered [Ag/Al_2_O_3_] (12.5 wt.% Ag) material after 1^st^ and 5^th^ reaction cycles, as well as for the calcined material after 1^st^ use of reaction, was performed (Figure S6, Supporting Information). Compared to the fresh version of the system, DRX analysis of the material recovered after 1^st^ use showed the disappearance of the diffraction signals corresponding to AgAlO_2_ species and an overall nanoparticle size of 41 nm, in comparison with the 34 nm determined for the fresh material. After applying a calcination step to this recovered material at 300 °C, DRX analysis showed a more similar pattern to the fresh version of the system, detecting AgAlO_2_ species. However, an increased overall nanocrystalline particle size of 50 nm was determined employing the Scherrer equation (Figure S6, Supporting Information). XRPD analysis of the material after 5^th^ use of the reaction displayed diffraction peaks assignable to silver(0) nanocrystalline and AgAlO_2_ species, but with a lower degree of crystallinity. Complementarily, HAADF‐HRSTEM and STEM‐XEDS analysis also provided valuable information for the morphological study of the recovered nanocatalysts (Figures S11–S13, Supporting Information). After 1^st^ use, the recovered material showed HAADF‐HRSTEM and XEDS images analysis very similar to the fresh version of the catalyst, with an overall silver nanoparticle size of 3.4 nm, only slightly increased from 2.2 nm. In the case of HAADF‐HRSTEM analysis of the material recovered after the 5^th^ use, a higher grade of nanoparticle aggregation and an overall nanoparticle size of 7.4 nm could be determined (Figures S11–S13, Supporting Information).

**Scheme 2:**
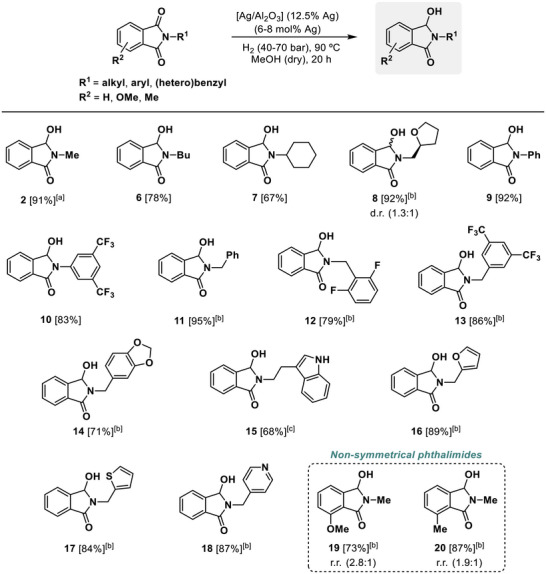
Synthesis of 3‐hydroxyisoindolinones via [Ag/Al_2_O_3_]‐catalyzed selective monohydrogenation of phthalimides. Standard reaction conditions: phthalimide (0.25 mmol), [Ag/Al_2_O_3_] material (12.5 wt.% Ag in the material, 6 mol% Ag), 1 mL of MeOH (previously dried with 4 Å MS), H_2_ (60 bar) at 90 °C during 20 h. [a] Run with H_2_ (40 bar). [b] Run with 8 mol% of Ag. [c] Run with H_2_ (70 bar). Isolated yields are given between brackets. Diastereomeric ratio (d.r.) of product **8** and regioisomeric ratio (r.r.) of products **19** and **20** determined by ^1^H NMR are shown between parentheses. For compounds **19** and **20** only the major regioisomer is represented.

Considering the optimal efficiency and demonstrated reusability of the [Ag/Al_2_O_3_] (12.5 wt.% Ag) material for promoting the selective monohydrogenation of **1** to **2**, we became interested in showing the potential synthetic applicability of the developed silver nanocatalyst for performing the monohydrogenation of several *N*‐substituted phthalimides to the corresponding 3‐hydroxyisoindolinones (**Scheme** **2**). Generally, standard reaction conditions consisting of 90 °C, 60 bar of H_2_ and 6 mol% Ag catalyst during 20 h were established for the substrate scope, although for specific cases, some of the reaction parameters were modified accordingly in order to improve 3‐hydroxylactam yield. Under these conditions, but at a lower H_2_ pressure of 40 bar, hemiamidal **2** could be isolated with a 91% yield. In the case of *N*‐butyl and *N*‐cyclohexyl hydroxylactams **6** and **7**, very good isolated yields of 78 and 67%, respectively, were also obtained, but requiring 60 bar of H_2_. By employing the same pressure but an increased amount of catalyst loading (8 mol% Ag), a tetrahydrofuran ring‐containing hydroxylactam **8** was accessed in a 92% isolated yield as a diastereomeric mixture with a 1.3:1 ratio, calculated by ^1^H NMR (Scheme 2). Interestingly, two *N*‐aryl substituted phthalimides, one of them containing two CF_3_ groups in the aromatic ring, were also studied at standard conditions. This kind of imides represent a great challenge regarding selectivity control to the formation of hydroxylactam, as they are more prone to undergo C─N hydrogenolysis, giving the phthalide/diol product and the corresponding aniline.^[^ ^61^, ^62^, ^63^ ^]^ This is due to the easier tendency of anilines to act as leaving groups, in comparison with aliphatic/benzylic amines. Gratifyingly, our developed nanocatalyst was able to afford *N*‐aryl hydroxylactams **9** and **10** with excellent isolated yields (92 and 83%, respectively, Scheme 2).

Furthermore, several *N*‐benzyl substituted phthalimides, containing a functional group sensitive to hydrogenation conditions,^[^ ^83^ ^]^ were also studied under our catalytic hydrogenation protocol. Applying 60 bar of H_2_ in the presence of 8 mol% Ag, *N*‐benzyl 3‐hydroxylactams **11**, **12,** and **13** were efficiently produced in 79–95% yields and obtained after isolation. In fact, such *N*‐benzyl phthalimides featuring ‐F atoms or ‐CF_3_ groups, in *orto*, *orto‐* or *meta*, *meta‐* positions, displayed a very good group tolerance under the conditions employed. Additionally, another *N*‐benzyl substituted phthalimide bearing an electron‐donating group (EDG) such as the dioxolane fragment, could also be successfully monohydrogenated to the desired hydroxylactam **14** with 71% yield. The synthesis of this type of 3‐hydroxylactam containing an EDG is especially challenging due to the possible formation of a *N*‐acyliminium intermediate that could react by Friedel–Crafts intramolecular cyclization, favored in this case.^[^ ^17^, ^18^ ^]^ However, with our catalytic protocol, we observed a full selectivity to the obtaining of the desired hemiamidal derivative **14**. To our delight, 3‐hydroxylactams **15**, **16,** and **17** featuring electron‐rich heterocycles such as a non‐protected indole, furan, and thiofuran were also accessed with good to excellent isolated yields (68, 89, and 84%, respectively), and showing total tolerance to the preservation of the heteroaromatic rings. Moreover, 3‐hydroxylactam derivative **18** bearing an electron‐poor ring as pyridine was also synthesized in 87% isolated yield with full selectivity employing our hydrogenative catalytic strategy (Scheme 2). It is important to note that in none of these substrates, debenzylation of the *N*‐benzyl moiety, a very favored reaction, was observed.

It is relevant to note that some limitations in the substrate scope were detected and they are showcased in Scheme S1 (Supporting Information). The catalytic system is not active enough toward a *N*‐benzyl substituted phthalimide with a boronic ester substitution, several symmetrically halogen substituted *N*‐methylphthalimides, and two azaphthalimides, in part because of solubility problems. In addition, poor selectivity was detected in the case of two *N*‐(homo)benzyl phthalimides.

Finally, our catalytic methodology was evaluated toward the monohydrogenation of 4‐substituted *N*‐methylphthalimides bearing a methyl or methoxy group in their aromatic ring and, hence, being non‐symmetrical phthalimides. In those cases, both carbonyl groups at the imide are chemically different, and two different regioisomers can be obtained. In the presence of the silver nanocatalyst, ring‐substituted 3‐hydroxyisoindolinones **19** and **20** were obtained in 73 and 87% yield as a mixture of regioisomers, respectively, with 2.8:1 and 1.9:1 regioisomeric ratios (calculated by ^1^H NMR). In both cases, the monohydrogenation was slightly driven to the carbonyl group with a lesser steric hindrance. A better regioselectivity was achieved in the case of the 4‐methoxysubsituted phthalimide, probably due to its more electron‐donating character (Scheme 2). This fact could suggest that better regioselectivity levels could be reached in the case of substrates substituted with more electron‐donating groups or steric hindrance, such as *N*,*N*‐dimethylamino or trimethylsilyl functionalities, respectively.

Overall, more than 15 different 3‐hydroxyisoindolinones have been synthesized by the monohydrogenation of the parent phthalimides with good to excellent isolated yields by applying our selective catalytic protocol based on the use of an alumina‐supported silver nanocatalyst [Ag/Al_2_O_3_] (12.5 wt.% Ag). Remarkably, any other potential byproducts coming from C─N hydrogenolysis (mono or dual) of the imide bond, C3‐methoxylated product from the reductive alkoxylation via a generated in situ *N*‐acyliminium ion using methanol as solvent,^[^ ^70^, ^71^ ^]^ possible undesired intramolecular cyclization products,^[^ ^8^, ^9^, ^10^, ^17^, ^18^, ^19^ ^]^ aryl ring hydrogenation or debenzylation of the *N*‐benzyl moieties have not been observed (Scheme 2).

Until this point, we have demonstrated that [Ag/Al_2_O_3_] (12.5% Ag) nanomaterial constitutes an efficient solid catalyst to perform the selective monohydrogenation of phthalimides to the corresponding 3‐hydroxylactams. Moreover, we have been able to elucidate important aspects regarding the most active catalyst structure. Then, with the goal to get further insight into the nature, mainly regarding oxidation state, of the silver active catalytic species that are involved in the reaction mechanism, we performed complementary UV–vis DRS (Figure S14, Supporting Information) and X‐ray Photoelectron Spectroscopy (XPS) studies (Figure S15 and Table S7, Supporting Information) of the fresh materials as well as the materials after being subjected to reaction conditions. As it was previously commented, UV–vis DRS analysis of fresh [Ag/Al_2_O_3_] (12.5% Ag) suggested the major presence of Ag^+^ cations covering Ag^0^ nanoparticles surface, although Ag_n_^δ+^ y Ag_n_° clusters were also detected to a minor extent (see Figure 4C). However, to complement this and better understand the exact real nature of Ag centers constituting the active site in the monohydrogenation of **1**, the effect of reaction conditions onto Ag supported nanoaggregates across the alumina matrix was studied by UV–vis DR spectroscopy. Figure S14 (Supporting Information) shows the UV–vis DRS analysis of the recovered [Ag/Al_2_O_3_] solid after being used in a reaction cycle of formation of **2** from **1**. Interestingly, a band with a maximum at 402 nm was detected, which is assignable to Ag^0^ NPs as the main exposed species onto the material surface after reaction, indicating the reduction of originally detected Ag^+^ species and suggesting that Ag^0^ NPs can have a relevant role in the catalytic phenomena.^[^ ^74^, ^75^, ^76^, ^77^ ^]^ UV–vis DRS analysis of the recovered solid, but calcined at 300 °C under air flow during 3 h before measurement, showed the disappearance of the characteristic band at 402 nm. In addition, this material only presents absorption at wavelengths to 300 nm, which indicates the main presence of Ag^+^ cations, and, in less extent, Ag_n_^δ+^ species at the surface of the material, with the total disappearance of the surface Ag^0^ NPs.^[^ ^74^ ^]^

To further confirm the Ag oxidation states nature at the most exposed atomic layers, we performed XPS studies of fresh and recovered [Ag/Al_2_O_3_] nanomaterials. However, for the specific case of silver, there is no significant difference between binding energy (eV) values assignable to Ag^0^ or Ag^+^ species (Figure S15, Supporting Information, up).^[^ ^84^, ^85^, ^86^, ^87^, ^88^, ^89^, ^90^, ^91^ ^]^ Therefore, a more exhaustive analysis was required, which involved the employment of the modified Auger parameter (α’, eV). By employing this approximation, the binding energy (BE) of Ag 3d_5/2_ and the kinetic energy (KE) of Ag M_5_N_45_N_45_ (α’ = BE + KE) Auger contributions were considered (Figure S15 and Table S7, Supporting Information). XPS analysis of fresh [Ag/Al_2_O_3_] system showed a BE value of Ag 3d_5/2_ and KE value of Ag M_5_N_45_N_45_ of 368.4 and 349.5, respectively, generating an Auger parameter of 717.9 eV, which could be assigned to Ag^+^ species (see Table S7, Supporting Information).^[^ ^91^ ^]^ On the other hand, XPS analysis of the recovered silver material after 1st cycle showed a slight decrease in the BE value of Ag 3d_5/2_ up to 368.2 eV, and KE value of Ag M_5_N_45_N_45_ increased until 351.3 eV (Figure S15, Supporting Information). As a consequence, the Auger parameter was increased up to 719.5 eV, being in clear consonance with surface silver species reduction in the material recovered after the reaction cycle (Table S7, Supporting Information).^[^ ^91^ ^]^

**Scheme 3:**
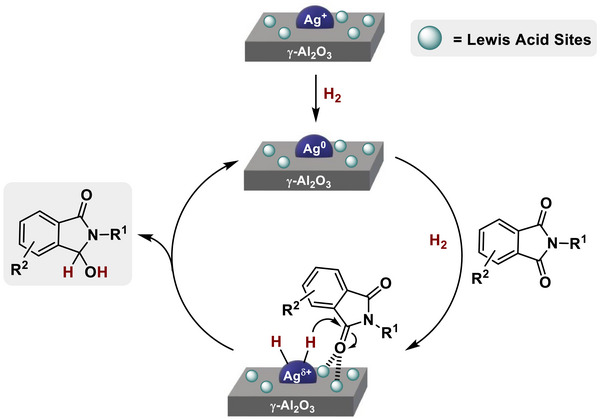
Proposed reaction mechanism for the [Ag/Al_2_O_3_]‐catalyzed monohydrogenation of phthalimides to 3‐hydroxyisoindolinones.

With all this information in hand, it was possible to propose a plausible mechanism for the monohydrogenation of phthalimides to the corresponding 3‐hydroxyisoindolinones (**Scheme** **3**). The first step of the overall mechanism involves the catalyst activation through the reduction of the surface Ag^+^ species to Ag^0^, as it has been suggested by the UV–vis DRS and XPS studies. Once the Ag^0^ species are present on the surface of the catalyst, an activation/dissociation of molecular hydrogen and transference for the hydrogenation of the carbonyl is proposed, together with the simultaneous carbonyl group imide activation performed by Al^3+^ Lewis acid centers. The fact that our most active catalyst, [Ag/Al_2_O_3_] (12.5% Ag) nanomaterial, presented an increased amount of Lewis acid sites and a large number of Ag accessible species, supports that this step potentially requires a synergistic environment between silver centers and Lewis acid sites present at the alumina matrix.^[^ ^63^ ^]^ Then, this step affords the desired hemiamidal with the total preservation of the nature of (hetero)aromatic fragments present at the imide and avoiding the complete reduction of the carbonyl group to produce the undesired lactam.

## 3 Conclusion

To sum up, in this work, we have been able to design a general heterogeneous silver‐catalyzed protocol to fully control the hydrogenation selectivity of phthalimides to the first step. Thus, with our system, we have performed the monohydrogenation of phthalimides to the corresponding 3‐hydroxyisoindolinones with full chemoselectivity. Moreover, a complete tolerance to the presence of (hetero)aromatic rings has been shown. By tuning different synthetic parameters, performing an exhaustive characterization and catalytically evaluating the different obtained materials, we determined that [Ag/Al_2_O_3_] (12.5 wt.% Ag) nanostructured system, composed by silver nanoaggregates with an overall particle size of 2.3 nm homogeneously distributed across the alumina matrix, results to be an effective, robust and recyclable catalyst to promote the selective carbonyl monohydrogenation of a wide range of phthalimides to the desired 3‐hydroxyisoindolinones. In addition, we have performed structure‐reactivity investigations which allowed us to deduce that an intimate contact between accessible Ag^0^ sites and Lewis acidic centers (Al^3+^ atoms from support) is crucial to the catalytic system to proceed efficiently. By applying this new nanostructured silver‐based material, a wide spectrum of structurally significant 3‐hydroxylactams has been selectively accessed, opening the door to new interesting avenues in the area of carboxylic acid derivatives hydrogenative valorizations employing heterogeneous catalysis. In our opinion, the work presented here goes in the right direction toward the rational design of nanostructured catalysts with general synthetic applicability toward a more sustainable organic chemistry.

## 4 Experimental Section

### General Information

All the chemicals were obtained from commercial sources and were used without further purification otherwise indicated. Metallic precursors and the solid supports used during nanomaterials preparation were purchased from Sigma–Aldrich ([Ag(acac)], [Pd(acac)_2_], [Pt(acac)_2_], [Ni(acac)_2_], [Fe(acac)_3_], [Co(acac)_3_], [Cu(acac)_2_], SiO_2_, ZnO, CeO_2_, TiO_2_, ZrO_2_, Nb_2_O_5,_ and HAP) and ABCR (γ‐Al_2_O_3_, ref. AB255279). Molecular sieves (4Å MS), and all the screened solvents (anhydrous *n*‐heptane, anhydrous THF, 1,4‐dioxane, anhydrous CPME, α,α,α‐trifluorotoluene, anhydrous toluene, anhydrous and HPLC grade MeOH, HPLC grade *i*‐PrOH, and HPLC grade *t*‐AmylOH) were also purchased from Sigma–Aldrich. 4Å MS were activated at 300 °C under vacuum for 3 h before use. 4Å MS were used in the initial optimization of the reaction conditions and for the process of drying HPLC‐grade MeOH, *i*‐PrOH, and *t*‐AmylOH solvents. The phthalimides used as starting materials in the substrate scope study were also acquired from commercial sources (ABCR, BLD, and Sigma–Aldrich) when available or prepared as detailed in a previous work from the group.^[^ ^51^ ^]^

### General Procedure for the Preparation of Solid Materials

The desired metal precursor and 50 mL of acetone were sequentially introduced into a 100 mL round‐bottom wide‐mouth flask. After 10 min of stirring, the support (1 g) was added, and the flask was stirred for 4 h. Once finished the impregnation, the solvent was distilled under vacuum using a rotavapor. Finally, the solid was homogenized and dispersed with a mortar, and a calcination process under air flow at the indicated temperature was applied to obtain the desired material. After carrying out the synthesis, the real metal content of each nanomaterial was determined using the ICP‐AES technique. This result was used to name the systems obtained as [M/support] (x%), where x represents the real weight percentage of the metal determined (see Tables S4–S7, Supporting Information). As a representative example, [Ag/γ‐Al_2_O_3_] (4.2% Ag) material was prepared by adding 0.4 mmol of [Ag(acac)] and 1 g of Al_2_O_3_ as the solid matrix. Once finished the impregnation process, the material was calcined under air flow at 500 °C for 3 h (with a heating ramp of 2 °C min^−1^). After carrying out the synthesis, it was determined a 4.2 wt.% of Ag in the prepared solid [Ag/Al_2_O_3_] by ICP‐AES. For more detailed information about the specific preparation of the materials, see section 2.2 of the Supporting Information.

### General Catalytic Procedures–Hydrogenation of *N*‐methylphthalimide (1)

A 8 mL glass vial containing a stirring bar was sequentially charged with *N*‐methylphthalimide **1** (0.25 mmol), the corresponding amount of catalytic material [M/support] (x% M) (considering the metal content determined by ICP‐AES), 4Å MS (previously activated at 300 °C under vacuum for 3 h) or not, *n*‐dodecane (20 µL) as an internal standard and 1 mL of the desired solvent. Afterward, the reaction vial was capped with a septum equipped with a syringe and set in the alloy plate, which was then placed into a 300 mL autoclave. Once sealed, the autoclave was purged three times with 20 bar of H_2,_ and the desired H_2_ pressure was charged, then, the autoclave was placed into an aluminium block preheated at the indicated reaction temperature on a stirring plate at 750 rpm. Once the reaction was completed, the autoclave was cooled down with the help of an ice bath, carefully depressurized, and opened. Finally, the reaction mixture was diluted with EtOAc, centrifuged, and the liquid fraction was analyzed by GC.

### General Catalytic Procedures–Hydrogenation of Phthalimides to 3‐hydroxylactams in the Presence of [Ag/Al_2_O_3_] Nanomaterial

A 8 mL glass vial containing a magnetic stirrer was sequentially charged with the corresponding phthalimide (0.25 mmol), [Ag/Al_2_O_3_] (12.5 wt.% Ag) material (6 or 10 mol% Ag), and 1 mL of dry MeOH (previously dried with 4 Å MS). Afterward, the reaction vial was capped with a septum equipped with a syringe and set in the alloy plate, which was then placed into a 300 mL autoclave. Once sealed, the autoclave was purged three times with 20 bar of H_2,_ and the desired H_2_ pressure was charged, then, the autoclave was placed into an aluminium block preheated at the indicated reaction temperature on a stirring plate at 750 rpm. Once the reaction was completed, the autoclave was cooled down with the help of an ice bath, carefully depressurized, and opened. At this point, the reaction mixture was centrifuged and washed with EtOAc, MeOH, and CH_2_Cl_2_. Finally, each compound of interest was isolated by column chromatography, making use of *n*‐hexane:EtOAc or CH_2_Cl_2_:MeOH mixtures.

## Conflict of Interest

The authors declare no conflict of interest.

## Author Contributions

C.L.‐G. performed all the experimental work related to materials preparation and characterization, conducted the catalytic experiments, the isolation of 3‐hydroxylactam compounds, and the interpretation of data results, and also collaborated in the writing of the manuscript. R.A. and J.R.C.‐A. designed and supervised the project, interpreted all the data obtained, and wrote the manuscript. All the authors revised the manuscript.

## Supporting information



Supporting Information

## Data Availability

The data that support the findings of this study are available in the supplementary material of this article.

## References

[1] R. A. Shenvi , D. P. O'Malley , P. S. Baran , Acc. Chem. Ress. 2009, 42, 530. 10.1021/ar800182r PMC2765532 19182997

[2] M. J. Climent , A. Corma , S. Iborra , ChemSusChem 2009, 2, 500. 19396883 10.1002/cssc.200800259

[3] M. J. Climent , A. Corma , S. Iborra , Chem. Rev. 2011, 111, 1072. 21105733 10.1021/cr1002084

[4] R. A. Sheldon , Chem. Soc. Rev. 2012, 41, 1437. 22033698 10.1039/c1cs15219j

[5] M. J. Climent , A. Corma , S. Iborra , M. J. Sabater , ACS Catal. 2014, 4, 870.

[6] F. Zaera , Chem. Rev. 2022, 122, 8594. 35240777 10.1021/acs.chemrev.1c00905

[7] M. J. Moolenaar , W. N. Speckamp , H. Hiemstra , E. Poetsch , M. Casutt , Chem. Int. Ed. 1995, 34, 2391.

[8] W. N. Speckamp , M. J. Moolenaar , Tetrahedron 2000, 56, 3817.

[9] B. E. Maryanoff , H.‐C. Zhang , J. H. Cohen , I. J. Turchi , C. A. Maryanoff , Chem. Rev. 2004, 104, 1431. 15008627 10.1021/cr0306182

[10] J. Royer , M. Bonin , L. Micouin , Chem. Rev. 2004, 104, 2311. 15137793 10.1021/cr020083x

[11] I. T. Raheem , P. S. Thiara , E. A. Peterson , E. N. Jacobsen , J. Am. Chem. Soc. 2007, 129, 13404. 17941641 10.1021/ja076179w

[12] T. Yang , L. Campbell , D. J. Dixon , J. Am. Chem. Soc. 2007, 129, 12070. 17877351 10.1021/ja074550+

[13] M. E. Muratore , C. A. Holloway , A. W. Pilling , R. I. Storer , G. Trevitt , D. J. Dixon , J. Am. Chem. Soc. 2009, 131, 10796. 19606900 10.1021/ja9024885

[14] S. Peixoto , T. M. Nguyen , D. Crich , B. Delpech , C. Marazano , Org. Lett. 2010, 12, 4760. 20882970 10.1021/ol101783c

[15] U. Martínez‐Estibalez , A. Gómez‐SanJuan , O. García‐Calvo , E. Aranzamendi , E. Lete , N. Sotomayor , Eur. J. Org. Chem. 2011, 2011, 3610.

[16] Y.‐Y. Huang , C. Cai , X. Yang , Z.‐C. Lv , U. Schneider , ACS Catal. 2016, 6, 5747.

[17] P. Wu , T. E. Nielsen , Chem. Rev. 2017, 117, 7811. 28493667 10.1021/acs.chemrev.6b00806

[18] Y. Quevedo‐Acosta , I. D. Jurberg , D. Gamba‐Sánchez , Eur. J. Org. Chem. 2022, 2022, 202200432.

[19] A. J. Basson , M. G. McLaughlin , Tetrahedron 2022, 114, 132764.

[20] Z. Malinowski , E. Fornal , A. Stachniuk , M. Nowak , Molecules 2022, 27, 8319. 36500412 10.3390/molecules27238319 PMC9737834

[21] M. E. Ernst , M. A. Fravel , Am. J. Hypertens. 2022, 35, 573. 35404993 10.1093/ajh/hpac048

[22] K. Kawakami , H. Futami , J. Takahara , K. Yamaguchi , Biochem. Biophys. Res. Commun. 1996, 219, 778. 8645257 10.1006/bbrc.1996.0310

[23] W. C. Lien , T. Y. Chen , S. Y. Sheu , T. C. Lin , F. C. Kang , C. H. Yu , T. S. Kuan , B. M. Huang , C. Y. Wang , J. Cell. Biochem. 2018, 119, 4729. 29280173 10.1002/jcb.26652

[24] S. Kitada , J. M. Zapata , M. Andreeff , J. C. Reed , Blood 2000, 96, 393. 10887097

[25] M. A. Dickson , M. S. Gordon , G. Edelman , J. C. Bendell , R. R. Kudchadkar , P. M. LoRusso , S. H. Johnston , D. O. Clary , G. K. Schwartz , Invest. New. Drugs 2015, 33, 349. 25476894 10.1007/s10637-014-0191-5

[26] Z.‐I. Horii , C. Iwata , Y. Tamura , J. Org. Chem. 1961, 26, 2273.

[27] Y. Kondo , B. Witkop , J. Org. Chem. 1968, 33, 206. 5634894 10.1021/jo01265a039

[28] S. Luo , C. A. Zificsak , R. P. Hsung , Org. Lett. 2003, 5, 4709. 14627421 10.1021/ol030114q

[29] X.‐h. Yuan , M.‐j. Zhang , C.‐q. Kang , H.‐q. Guo , X.‐p. Qiu , L.‐x. Gao , Synth. Commun. 2006, 36, 435.

[30] E. Conchon , F. Anizon , B. Aboab , M. Prudhomme , Synthesis 2008, 2569. 10.1021/jm070664k 17722905

[31] L. Andna , L. Miesch , Org. Lett. 2018, 20, 3430. 29790768 10.1021/acs.orglett.8b01407

[32] G. Ding , B. Lu , Y. Li , J. Wan , Z. Zhang , X. Xie , Adv. Synth. Catal. 2015, 357, 1013.

[33] G. Ding , C. Li , Y. Shen , B. Lu , Z. Zhang , X. Xie , Adv. Synth. Catal. 2016, 358, 1241.

[34] Y. Kawamata , K. Hayashi , E. Carlson , S. Shaji , D. Waldmann , B. J. Simmons , J. T. Edwards , C. W. Zapf , M. Saito , P. S. Baran , J. Am. Chem. Soc. 2021, 143, 16580. 34596395 10.1021/jacs.1c06572 PMC8711284

[35] Y. Bai , L. Shi , L. Zheng , S. Ning , X. Che , Z. Zhang , J. Xiang , Org. Lett. 2021, 23, 2298. 33683904 10.1021/acs.orglett.1c00430

[36] Y. Wang , J. Zhao , T. Qiao , J. Zhang , G. Chen , Chin. J. Chem. 2021, 39, 3297.

[37] N. Topolovčan , M. Gredičak , Org. Biomol. Chem. 2021, 19, 4637. 33978006 10.1039/d1ob00164g

[38] D.‐M. Yan , Q.‐Q. Zhao , L. Rao , J.‐R. Chen , W.‐J. Xiao , Chem. Eur. J. 2018, 24, 16895. 30126062 10.1002/chem.201804229

[39] Y. Yu , Z. Liang , L. Zhang , Y. Lin , M. Liu , S. Wang , M. Huang , L. Cai , S. Cai , Org. Chem. Front. 2023, 10, 4400.

[40] Q. Elliott , G. dos Passos Gomes , C. J. Evoniuk , I. V. Alabugin , Chem. Sci. 2020, 11, 6539. 34094120 10.1039/c9sc06511c PMC8159354

[41] K. Nozawa‐Kumada , Y. Matsuzawa , K. Ono , M. Shigeno , Y. Kondo , Chem. Commun. 2021, 57, 8604. 10.1039/d1cc02870g 34368822

[42] J. E. Hong , H. Jeon , J.‐H. Kwak , S. Lee , Y. Park , J. Org. Chem. 2025, 90, 8152. 40493805 10.1021/acs.joc.5c00498

[43] P. A. Dub , T. Ikariya , ACS Catal. 2012, 2, 1718.

[44] A. M. Smith , R. Whyman , Chem. Rev. 2014, 114, 5477. 24580716 10.1021/cr400609m

[45] J. Pritchard , G. A. Filonenko , R. van Putten , E. J. M. Hensen , E. A. Pidko , Chem. Soc. Rev. 2015, 44, 3808. 25941799 10.1039/c5cs00038f

[46] J. R. Cabrero‐Antonino , R. Adam , V. Papa , M. Beller , Nat. Commun. 2020, 11, 3893. 32753681 10.1038/s41467-020-17588-5 PMC7403344

[47] R. Qu , K. Junge , M. Beller , Chem. Rev. 2023, 123, 1103. 36602203 10.1021/acs.chemrev.2c00550

[48] H. Yang , H. Garcia , C. Hu , Green Chem. 2024, 26, 2341.

[49] C. R. Kemnitz , M. J. Loewen , J. Am. Chem. Soc. 2007, 129, 2521. 17295481 10.1021/ja0663024

[50] Y.‐C. Yuan , C. Bruneau , R. Gramage‐Doria , Synthesis 2018, 50, 4216.

[51] C. Lluna‐Galán , J. C. Arango‐Daza , D. Gómez , P. Concepción , R. Sun , J. J. Calvino , L. Simonelli , R. Adam , J. R. Cabrero‐Antonino , Nat. Commun. 2025, 16, 4119. 40316551 10.1038/s41467-025-59514-7 PMC12048504

[52] H. Adkins , H. I. Cramer , J. Am. Chem. Soc. 1930, 52, 4349.

[53] A. J. McAlees , R. McCrindle , D. W. Sneddon , J. Chem. Soc., Perkin Trans. 1 1977, 2038.

[54] A. Arévalo , S. Ovando‐Segovia , M. Flores‐Alamo , J. J. García , Organometallics 2013, 32, 2939.

[55] R. Aoun , J.‐L. Renaud , P. H. Dixneuf , C. Bruneau , Angew. Chem., Int. Ed. 2005, 44, 2021. 10.1002/anie.200462996 15724255

[56] A. M. Maj , I. Suisse , N. Pinault , N. Robert , F. Agbossou‐Niedercorn , ChemCatChem 2014, 6, 2621.

[57] M. Ito , A. Sakaguchi , C. Kobayashi , T. Ikariya , J. Am. Chem. Soc. 2007, 129, 290. 17212405 10.1021/ja067777y

[58] M. Ito , C. Kobayashi , A. Himizu , T. Ikariya , J. Am. Chem. Soc. 2010, 132, 11414. 20672811 10.1021/ja105048c

[59] C. Wu , J. Wang , X. Zhang , R. Zhang , B. Ma , Org. Chem. Front. 2021, 8, 6530.

[60] L. Shi , X. Tan , J. Long , X. Xiong , S. Yang , P. Xue , H. Lv , X. Zhang , Chem. ‐ Eur. J. 2017, 23, 546. 27807893 10.1002/chem.201604904

[61] A. Kumar , T. Janes , N. A. Espinosa‐Jalapa , D. Milstein , J. Am. Chem. Soc. 2018, 140, 7453. 29812921 10.1021/jacs.8b04581 PMC6502447

[62] U. K. Das , T. Janes , A. Kumar , D. Milstein , Green Chem. 2020, 22, 3079.

[63] X. Liu , C. Wu , P. Bai , Y. Miao , Y. Hu , Y. Xie , Org. Lett. 2023, 25, 3066. 37088958 10.1021/acs.orglett.3c00887

[64] A. J. McAlees , R. McCrindle , J. Chem. Soc. C 1969, 2425.

[65] S. Takebayashi , J. M. John , S. H. Bergens , J. Am. Chem. Soc. 2010, 132, 12832. 20734992 10.1021/ja105783u

[66] C. Lluna‐Galán , L. Izquierdo‐Aranda , P. de la Iglesia‐Gómez , C. Béchet , P. Vidal‐Puyuelo , J. C. Arango‐Daza , R. Adam , J. R. Cabrero‐Antonino , ACS Sustainable Chem. Eng. 2025, 13, 12328. 40814396 10.1021/acssuschemeng.5c05996 PMC12345408

[67] N. Bogdanchikova , F. C. Meunier , M. Avalos‐Borja , J. P. Breen , A. Pestryakov , Appl. Catal., B 2002, 36, 287.

[68] A. S. Lanje , S. J. Sharma , Ramch, R. Pode , J. Chem. Pharm. Res. 2010, 2, 478.

[69] M. H. Ali , M. A. K. Azad , K. A. Khan , M. O. Rahman , U. Chakma , A. Kumer , ACS Omega 2023, 8, 28133. 37576647 10.1021/acsomega.3c01261 PMC10413482

[70] J. R. Cabrero‐Antonino , I. Sorribes , K. Junge , M. Beller , Angew. Chem., Int. Ed. 2016, 55, 387. 10.1002/anie.201508575 26555216

[71] J. R. Cabrero‐Antonino , R. Adam , V. Papa , M. Holsten , K. Junge , M. Beller , Chem. Sci. 2017, 8, 5536. 28970933 10.1039/c7sc01175j PMC5618770

[72] R. D. Shannon , K. H. Gardner , R. H. Staley , G. Bergeret , P. Gallezot , A. Auroux , J. Phys. Chem. C 1985, 89, 4778.

[73] D. Y. Yoon , J.‐H. Park , H.‐C. Kang , P. S. Kim , I.‐S. Nam , G. K. Yeo , J. K. Kil , M.‐S. Cha , Appl. Catal., B 2011, 101, 275.

[74] B. Inceesungvorn , J. López‐Castro , J. J. Calvino , S. Bernal , F. C. Meunier , C. Hardacre , K. Griffin , J. J. Delgado , Appl. Catal., A 2011, 391, 187.

[75] I. López‐Hernández , C. García , V. Truttmann , S. Pollitt , N. Barrabés , G. Rupprechter , F. Rey , A. E. Palomares , Catal. Today 2020, 345, 22.

[76] L. Izquierdo‐Aranda , R. Adam , J. R. Cabrero‐Antonino , ChemSusChem 2023, 16, 202300818. 10.1002/cssc.202300818 37486295

[77] F. Wang , J. Ma , G. He , M. Chen , C. Zhang , H. He , ACS Catal. 2018, 8, 2670.

[78] R. E. Hummel , T. Dubroca , Encyclopedia of Analytical Chemistry, John Wiley & Sons, Ltd., Hoboken, New Jersey 2014, p. 1.

[79] S. Ouyang , Z. Li , Z. Ouyang , T. Yu , J. Ye , Z. Zou , J. Phys. Chem. C 2008, 112, 3134.

[80] R. Chanerika , M. L. Shozi , H. B. Friedrich , ACS Omega 2022, 7, 4026. 35155897 10.1021/acsomega.1c05231 PMC8829924

[81] S. Hinokuma , Y. Kawabata , S. Matsuki , H. Shimanoe , S. Kiritoshi , M. Machida , J. Phys. Chem. C 2017, 121, 4188.

[82] S. Hinokuma , H. Shimanoe , Y. Kawabata , S. Matsuki , S. Kiritoshi , M. Machida , Catal. Today 2018, 303, 2.

[83] Y. Yamamoto , E. Shimizu , K. Ban , Y. Wada , T. Mizusaki , M. Yoshimura , Y. Takagi , Y. Sawama , H. Sajiki , ACS Omega 2020, 5, 2699. 32095693 10.1021/acsomega.9b03226 PMC7033673

[84] J. S. Hammond , S. W. Gaarenstroom , N. Winograd , Anal. Chem. 1975, 47, 2193.

[85] P. Weightman , P. T. Andrews , J. Phys. C Solid State Phys. 1980, 13, 3529.

[86] V. K. Kaushik , J. Electron Spectros. Relat. Phenomena 1991, 56, 273.

[87] G. B. Hoflund , D. M. Minahan , J. Catal. 1996, 162, 48.

[88] M. P. Seah , I. S. Gilmore , G. Beamson , Interface Anal. 1998, 26, 642.

[89] S. Wodiunig , J. M. Keel , T. S. E. Wilson , F. W. Zemichael , R. M. Lambert , Catal. Lett. 2003, 87, 1.

[90] W. Diao , C. D. DiGiulio , M. T. Schaal , S. Ma , J. R. Monnier , J. Catal. 2015, 322, 14.

[91] NIST , X‐ray Photoelectron Spectroscopy (XPS) Database, Version 3.5, (accessed: February 2024).

