# Supplementary material for: Controlling Selectivity at the First Hydrogenation Level: Synthesis of 3‐Hydroxyisoindolines by Heterogeneously Silver‐Catalyzed Monohydrogenation of Phthalimides
Source: Adv Sci (Weinh). 2025 Oct 15;13(2):e16161. doi: 10.1002/advs.202516161 (PMC12786360; doi:10.1002/advs.202516161)
Supplement: Supplementary file 1 — Supporting Information [file ADVS-13-e16161-s001.pdf]

# SUPPORTING INFORMATION

# Controlling Selectivity at the First Hydrogenation Level: Synthesis of 3-Hydroxyisoindolines by Heterogeneously Silver-Catalyzed Monohydrogenation of Phthalimides

Carles Lluna-Galán,[a] Rosa Adam*[b] and Jose R. Cabrero-Antonino*[a]

[a]Instituto de Tecnología Química (UPV-CSIC),
Universitat Politècnica de València-Consejo Superior de Investigaciones Científicas,
Avd. de los Naranjos s/n, València, 46022 (Spain)
[b]Departament de Química Orgànica,
Facultat de Farmàcia, Universitat de València,
Avd. Vicent Andrés Estellés s/n, Burjassot, València, 46100 (Spain)

*E-mail: rosa.adam@uv.es; jcabrero@itq.upv.es

1. GENERAL INFORMATION

1.1. Materials characterization techniques

1.1.1. Elemental Analysis (EA)

1.1.2. Fourier-Transform Infrared Spectroscopy of Adsorbed Pyridine (Pyr-FTIR)

1.1.3. High-Angle Annular Dark-Field High-Resolution Scanning-Transmission Electron Microscopy (HAADF-HRSTEM) and Scanning-Transmission Energy-Dispersive X-Ray Spectroscopy (STEM-XEDS)

1.1.4. $H_2$-Temperature Programmed Reduction ($H_2$-TPR)

1.1.5. Inductively Coupled Plasma-Atomic Emission Spectroscopy (ICP-AES)

1.1.6. $N_2$ Physisorption

1.1.7. X-Ray Powder Diffraction (XRPD)

1.1.8. UV-Vis Diffuse Reflectance Spectroscopy (UV-Vis DRS)

1.1.9. X-Ray Photoelectron Spectroscopy (XPS)

1.2. Reaction monitoring and characterization methods for organic molecules

1.2.1. Gas Chromatography (GC)

1.2.2. Gas Chromatography-Mass Spectrometry (GC-MS)

1.2.3. Nuclear Magnetic Resonance (NMR)

1.2.4. Preparative Thin Layer Chromatography (PTLC) and column chromatography

1.2.5. Ultra-Performance Liquid Chromatography High-Resolution Mass Spectroscopy (UPLC-HRMS)

2. GENERAL EXPERIMENTAL PROCEDURES

2.1. General procedure for the preparation of solid materials

2.2. Specific procedures for the preparation of solid materials

2.3. Procedure for the kinetic studies by using [Ag/$Al_2O_3$] (12.5% Ag) nanomaterial

2.4. Procedure for the filtration and leaching tests of [Ag/$Al_2O_3$] (12.5% Ag) nanomaterial

2.5. Procedure for the recycling experiments of [Ag/$Al_2O_3$] (12.5% Ag) nanomaterial

3. COMPLEMENTARY CATALYTIC STUDIES

Table S1

Table S2

Figure S1

Scheme S1

4. [Ag/$Al_2O_3$] SYSTEM HETEROGENEITY EXPERIMENTS

4.1. Filtration tests

Figure S2

4.2. Recycling studies

Figure S3

5. CHARACTERIZATION OF THE MATERIALS

5.1. Inductively Coupled Plasma-Atomic Emission Spectroscopy (ICP-AES)

Table S3

Table S4

**Table S5**

**5.2. X-Ray Powder Diffraction (XRPD)**

**Figure S4**

**Figure S5**

**Figure S6**

**5.3. High-Angle Annular Dark-Field Scanning-Transmission Electron Microscopy (HAADF-STEM) and Scanning-Transmission Energy-Dispersive X-Ray Spectroscopy (STEM-XEDS)**

**Figure S7**

**Figure S8**

**Figure S9**

**Figure S10**

**Figure S11**

**Figure S12**

**Figure S13**

**5.4. Specific surface area and pore diameter**

**Table S6**

**5.5. UV-Vis Diffuse Reflectance Spectroscopy (UV-Vis DRS)**

**Figure S14**

**5.6. X-Ray Photoelectron Spectroscopy (XPS)**

**Figure S15**

**Table S7**

**5.7. $H_2$-Temperature Programmed Reduction ($H_2$-TPR)**

**Figure S16**

**6. CHARACTERIZATION DATA OF THE ISOLATED ORGANIC COMPOUNDS**

**7. NMR SPECTRA OF THE ISOLATED ORGANIC COMPOUNDS**

**8. SUPPLEMENTARY REFERENCES**

## 1. GENERAL INFORMATION

### 1.1. Materials characterization techniques

#### 1.1.1. Elemental analysis (EA)

With Elemental Analysis (EA) it is possible to determine the carbon, hydrogen, nitrogen, and sulfur of the catalyst through a combustion process. In this work, it was used to quantify the carbon present in the nanomaterials after the catalytic hydrogenation reaction. EA was carried out in an EURO EA Elemental Analysis from Eurovector. Experimentally, the samples were introduced in an Sn capsule into an oven at 1020 ºC. He was used as carrier gas (140 mL/min).

#### 1.1.2. Fourier-Transform Infrared Spectroscopy of Adsorbed Pyridine (Pyr-FTIR)

Monitoring the adsorption-desorption process of pyridine through infrared spectroscopy, it is possible to quantify the acidity of the solid materials. Both, Brønsted acid sites (BAS) (pyridinium ion band at 1555 $cm^{-1}$) and Lewis acid sites (LAS) (band at 1450 $cm^{-1}$) can be distinguished. The concentration of [BAS] and [LAS] (mmol/g) was calculated according to the methodology proposed by Emeis (Eq. 1 and 2, respectively).[1] Where A is the integrated absorbance of FTIR signals, r is the radius of the pellet, and w is its weight.

$$[\mathrm{BAS}] = 1.88 \cdot \mathrm{A} \cdot \mathrm{r}^2 \cdot \mathrm{w}^{-1} \ (\mathrm{mmol/g}) \qquad \text{(Eq. 1)}$$

$$[\mathrm{LAS}] = 1.42 \cdot \mathrm{A} \cdot \mathrm{r}^2 \cdot \mathrm{w}^{-1} \ (\mathrm{mmol/g}) \qquad \text{(Eq. 2)}$$

Moreover, the strength distribution can also be studied by performing the desorption at different temperatures, being stronger these remaining adsorbed at higher temperatures. In our experiments, Pyr-FTIR measurements were performed in a Nicolet Is-10 Thermo FT-infrared spectrophotometer. Self-supported pellets (10 mg) were degassed under vacuum ($10^{-2}$ Pa) at 300 ºC for 12 h. Pyridine was then fluxed into the cell (650 Pa) and when equilibrium was achieved, the cell was degassed at a desired experiment temperature and cooled down to room temperature. At this point, FTIR was acquired. This sequence was carried out at 150 ºC (weak strength), 250 ºC (medium strength), and 350 ºC (high strength). In all cases, a spectrum was collected under a vacuum before pyridine adsorption and was used as background. This background was subtracted from each spectrum, and the absorbance was normalized to weight before calculations.

**NOTE:** When $Al_2O_3$ was the solid matrix, it was only possible to study the nature and quantify the Lewis acid sites, due to the necessity to incorporate chromatography $SiO_2$ (50 wt%) to facilitate the pellet preparation.

#### 1.1.3. High-Angle Annular Dark Field High-Resolution Scanning Transmission Electron microscopy (HAADF-HRSTEM) and Scanning-Transmission Energy-Dispersive X-Ray Spectroscopy (STEM-XEDS)

These techniques were used to study the effect of variations in the nanomaterial synthesis or the influence of the reaction conditions in the homogeneity and the dispersion grade of the Ag species on the $Al_2O_3$ surface. The samples were prepared by physical contact between the copper grids and the solid. They were analyzed in a JEOL TEM 2100F at 200 kV in the facilities of the Universitat Politècnica de València. To determine the average nanoparticle size in each sample, more than 200 particles were analyzed with the ImageJ software and fitted to a Gaussian distribution.

#### 1.1.4. $H_2$-Temperature Programmed Reduction ($H_2$-TPR)

The study of the effect of $H_2$ over the reducible species of the catalysts at a known temperature is used to characterize several physicochemical properties. In this work, hydrogen Temperature Programmed Reduction ($H_2$-TPR) was performed to study the Ag reducibility. $H_2$-TPR experiments were carried out in Micrometrics Autochem 2910 equipped with a Thermal Conductivity Detector (TCD) detector, using 50 mg of freshly calcined catalyst, and increasing the

temperature from room temperature to 800 ºC with a heating ramp of 10 ºC/min under 10% $H_2$/Ar (vol%) and a constant flow rate of 50 mL/ min.

### 1.1.5. Inductively Coupled Plasma-Atomic Emission Spectroscopy (ICP-AES)

The amount of metal contained in the fresh and used catalysts was determined by Inductively Coupled Plasma-Atomic Emission Spectroscopy (ICP-AES) using Varian 715-ES after dissolving the solid samples in HCl/$HNO_3$ solution (3:1 vol).

### 1.1.6. $N_2$ Physisorption

Nitrogen adsorption technique by the application of the Brunauer-Emmet-Teller (BET) method, was used to determine the surface area of the catalysts. Nitrogen adsorption isotherms were registered using an ASAP 2420 apparatus (Micromeritics) at -196 ºC. Before the analysis, 200 mg of the sample (sieve fraction 200-400 μm) were degassed at 400 ºC and ~$5\times10^{-6}$ bar overnight. The specific surface area was determined by applying the Brunauer-Emmett-Teller (BET) equation in the relative pressure range ($P/P_0$) of 0.05-0.25 of the isotherms.[2]

### 1.1.7. X-Ray Powder Diffraction (XRPD)

All the catalysts were analyzed with X-ray diffraction (XRD) to identify the crystalline phases. Powder XRD measurements were performed in Bragg-Brentano geometry using a PANalytical CUBIX diffractometer equipped with an X-Celerator detector using Cu Kα ($\lambda_1$ = 1.5406 Å, $\lambda_2$ = 1.5444 Å, $I_2/I_1$ = 0.5) radiation. The applied tube voltage and intensity were 45 kV and 40 mA, respectively. The length of the goniometer arm is 200 mm, and a fixed divergence slit with a 1/8 º aperture was applied. The scanning range was from 3.5 º to 90.0 º (2 θ), with a step of 0.020 º (2 θ) and an acquisition time of 35 s per step. The measurement was performed at 25 ºC while the sample was rotated at 0.5 revolutions per s.

### 1.1.8. UV-Vis Diffuse Reflectance Spectroscopy (UV-Vis DRS)

This technique was used to study the surface oxidation state of Ag-containing nanomaterials. Diffuse reflectance (UV-vis) spectra were obtained on a Cary 5000 (Agilent Technologies) instrument equipped with a Harrick "Praying Mantis" cell, using $BaSO_4$ as a reference for reflectance.

### 1.1.9. X-Ray Photoelectron Spectroscopy (XPS)

The surface composition and chemical state of Ag species were determined by X-ray Photoelectron Spectroscopy (XPS). The spectra were recorded with a SPECS spectrometer equipped with a Phoibos 150 MCD-9 multichannel analyzer, using a non-monochromatic Al Kα (1486.6 eV) X-ray source. The spectra were recorded with an X-ray power of 100 W, a pass energy of 30 eV, and an operating pressure of $10^{-9}$ mbar. The XPS spectra were referenced to the Al 2p component (74.2 eV), and spectral processing was performed using CASA XPS software and the NIST library. Furthermore, due to the slight differences in the binding energy values between the different Ag oxidation states, it was necessary to determine the modified Auger parameter (α', eV, Eq. 3). This parameter considers the contribution of the most intense signal of the kinetic energy (KE) of the Auger MNN transition ($M_5N_{45}N_{45}$) and the binding energy (BE) of the Ag 3d component ($3d_{5/2}$).

$$[\alpha'] = \mathrm{KE}\ (\mathrm{M_5N_{45}N_{45}}) + \mathrm{BE}\ \left(3\mathrm{d}_{5/2}\right)\ (\mathrm{eV}) \quad \text{(Eq. 3)}$$

### 1.2. Reaction monitoring and characterization methods for organic molecules

#### 1.2.1. Gas Chromatography (GC)

Gas Chromatography (GC) was used to monitor the reaction outcome. The analyses were performed in a Bruker 430-GC equipped with a 25 m capillary column of 5% phenylmethyl silicone, using *n*-dodecane as the internal standard and $N_2$ as carrier gas.

#### 1.2.2. Gas Chromatography-Mass Spectrometry (GC-MS)

All the isolated organic products were characterized by gas chromatography coupled to mass spectrometry (GC-MS), to ensure the structure of the obtained molecules and determine their structural fragmentations. The analyses were acquired on an Agilent 6890 Network gas chromatograph equipped with an HP-5 column (30 m, 0.32 mm, 0.25 $\mu$m) coupled to an Agilent 5973 Network mass selective detector.

#### 1.2.3. Nuclear Magnetic Resonance (NMR)

Nuclear Magnetic Resonance (NMR) is the reference technique to characterize organic molecules. In this work, all the obtained 3-hydroxyisoindolinones ($\omega$-hydroxyisoindolinones) and 3-substituted isoindolinones were properly characterized with monodimensional $^{1}H$, $^{13}C$, (and $^{19}F$) and DEPT. When required, also bidimensional spectra were acquired to ensure the molecule structure (HSQC, HMBC). Spectra were recorded on a Bruker 300 or Bruker 400 spectrometer. All chemical shifts ($\delta$) are reported in parts per million (ppm) and coupling constants ($J$) in hertz (Hz). Abbreviations used in the reported NMR experiments: d, doublet; dd, double doublet; s, singlet; t, triplet; q, quartet; m, multiplet. All chemical shifts are reported relative to residual proton solvents *i.e.*, to $CDCl_3$ peaks ($\delta$ 7.26 ppm for $^{1}H$ NMR and $\delta$ 77 ppm for $^{13}C$ NMR), $CD_3CN$ ($\delta$ 2.13 ppm for $^{1}H$ NMR and $\delta$ 118.26 ppm for $^{13}C$ NMR) or MeOD ($\delta$ 4.87 ppm for $^{1}H$ NMR and $\delta$ 49 ppm for $^{13}C$ NMR).

#### 1.2.4. Preparative Thin Layer Chromatography (PTLC) and column chromatography

All the imide hydrogenation products were isolated by silica column chromatography or preparative thin layer chromatography (PTLC). For PTLC, thin layer chromatography plates Uniplate$_{TM}$ (20x20 cm) from Miles Scientific were used and the organic compounds were extracted with EtOAc or $CH_2Cl_2$ after stirring for 2 h. When column chromatography was chosen, it was used Silica Gel Merck 60 with 0.04 – 0.06 mm of particle size (Merck 109385 reference) using different eluent mixtures (*n*-hexane/EtOAc or $CH_2Cl_2$/MeOH).

#### 1.2.5. Ultra-Performance Liquid Chromatography High-Resolution Mass Spectroscopy (UPLC-HRMS)

When available, the characterization in the literature was used for comparison with the NMR spectra; otherwise, UPLC-HRMS experiments were additionally done to confirm the novel structure. HRMS measurements of all isolated products were performed using the electrospray ionization technique in UPLC equipment.

## 2. GENERAL EXPERIMENTAL PROCEDURES

### 2.1. General procedure for the preparation of solid materials

The desired metal precursor and 50 mL of acetone were sequentially introduced in a 100 mL round-bottom wide-mouth flask. After 10 min of stirring, the support (1 g) was added, and the flask was stirred for 4 h. Once finished the impregnation, the solvent was distilled under vacuum using a rotavapor. Finally, the solid was homogenized and dispersed with a mortar, and a calcination process under air flow at the indicated temperature was applied to obtain the desired material. After carrying out the synthesis, the real metal content of each nanomaterial was determined using the ICP-AES technique. This result was used to name the systems obtained as [M/support] (x% M), where x represents the real weight percentage of the metal determined (see Tables S3-S5).

### 2.2. Specific procedures for the preparation of solid materials

**For the initial study of the influence of the solvent, pressure and temperature.** The material was prepared by adding 0.4 mmol of [Ag(acac)] and 1 g of $Al_2O_3$ as the solid matrix. Once finished the impregnation process, the material was calcined under air flow at 500 ºC for 3 h (with a heating ramp of 2 ºC/min). After carrying out the synthesis, by ICP-AES it was determined a 4.2 wt% of Ag in the prepared solid [Ag/$Al_2O_3$].

**For the metal study employing different [M/$Al_2O_3$] (x% M) systems.** All the materials were prepared by adding 0.4 mmol of metal precursor and 1 g of $Al_2O_3$ as the solid matrix. Once finished the impregnation process, the materials were calcined under air flow at 500 ºC for 3 h (with a heating ramp of 2 ºC/min). The used precursors were: [Ag(acac)], [Pd(acac)$_2$], [Pt(acac)$_2$], [Ni(acac)$_2$], [Fe(acac)$_3$], [Ni(acac)$_2$], [Co(acac)$_3$], and [Cu(acac)$_2$]. After carrying out the synthesis, the wt% of metal in each of the material was determined by ICP-AES (see Table S3).

**For the support study employing different [Ag/support] (x% Ag) systems.** All the materials were prepared by adding 0.4 mmol of [Ag(acac)] and 1 g of the solid matrix. Once finished the impregnation process, the materials were calcined under air flow at 500 ºC for 3 h (with a heating ramp of 2 ºC/min). The used supports were: nanopowder $Al_2O_3$, nanopowder $SiO_2$, $ZrO_2$ (activated at 240 ºC under airflow during 2 h, with a heating ramp of 3 ºC/min), nanopowder $CeO_2$ (activated at 500 ºC under air flow during 3 h, with a heating ramp of 5 ºC/min), nanopowder HAP (hydroxyapatite), nanopowder $TiO_2$, nanopowder ZnO, and $Nb_2O_5$. After carrying out the synthesis, the wt% of silver in each of the material was determined by ICP-AES (see Table S4).

**For the thermal treatment study employing [Ag/$Al_2O_3$] (4.2% Ag) system.** In this optimization step, to ensure the veracity of the experiments, all the thermal treatments were done with the same starting batch (5 x 1 g batch of theoretical [Ag/$Al_2O_3$] (4.2% Ag), *i.e.* 0.4 mmol Ag /g $Al_2O_3$ by synthesis). After the impregnation process, the obtained solid was calcined under air flow at different temperatures (300, 400, 500, 600, and 700 ºC) during 3 h with a heating ramp of 2 ºC/min.

**For the metal charge study employing [Ag/$Al_2O_3$] (x% Ag) systems.** In this optimization step, different Ag loadings in the formulations were evaluated. With this purpose, the corresponding solids with 0.1, 0.2, 0.4, 0.8, 1.2, 1.6 and 2 mmol of [Ag(acac)] in 1 g of $Al_2O_3$ were prepared. In all the cases, the solids were calcined under air flow at 700 ºC for 3 h, with a heating ramp of 2 ºC/min. After carrying out the synthesis, the wt% of Ag in each of the material was determined by ICP-AES (see Table S5).

**Synthesis of [Ag/$Al_2O_3$] (12.5% Ag).** [Ag(acac)] (1.2 mmol) was suspended in 50 mL of acetone. After 10 min of agitation, $Al_2O_3$ (1 g) was added, and the wet impregnation occurred for 4 h. The solvent was distilled under vacuum

with the help of a rotavapor, the solid was ground and calcined in flow at 700 ºC for 3 h, using a heating ramp of 2 ºC/min. After carrying out the synthesis, by ICP-AES it was determined a 12.5 wt% of Ag in the prepared solid [Ag/$Al_2O_3$].

### 2.3. Procedure for the kinetic studies by using [Ag/$Al_2O_3$] (x% Ag) nanomaterial

To a 25 mL Teflon-covered stainless-steel autoclave with a X-shape magnet, *N*-methylphthalimide **1** (0.75 mmol), the corresponding of [Ag/$Al_2O_3$] (x% Ag) nanomaterial (6 mol% of Ag), *n*-dodecane (60 µL) as an internal standard, and 3 mL of dry MeOH (previously dried with 4 Å MS) were added sequentially. The autoclave was closed and pressurized with $H_2$. After three purges, the desired pressure was charged, and the autoclave was placed into an aluminium block preheated at 90 ºC on a stirring plate at 1000 rpm. Periodically, aliquots of 50 µL were taken at different reaction times, diluted with EtOAc, centrifuged, and the liquid fraction was analyzed by GC.

### 2.4. Procedure for the filtration and leaching tests of [Ag/$Al_2O_3$] (12.5% Ag) nanomaterial

The filtration test was conducted as commented above for the realization of the kinetic study (see Section 2.3). Once the reaction started, three initial aliquots were taken at 5, 10, and 20 min to control the reaction. After this time, the autoclave was cooled with the help of an ice bath, carefully depressurized, and opened. The reaction mixture was transferred into a vial, centrifuged, and finally transferred into a new autoclave equipped with a stirring bar. The autoclave was closed and pressurized with $H_2$. After three purges with 20 bar of $H_2$, the desired pressure was charged (40 bar), and the autoclave was placed into an aluminium block preheated at 90 ºC. Periodically, aliquots of 50 µL were taken at different reaction times, diluted with EtOAc, centrifuged, and the liquid fraction was analyzed by GC (this result is shown in Figure S2). The same autoclave-opening procedure was done without catalyst removal, and the reaction continued with a similar yield.

Moreover, the possible leaching of metal species to the reaction mixture was also studied analyzing the metal content of both, the final catalyst and the reaction mixture. With this purpose, after reaction analysis, the solvent was removed with the help of a rotavapor, and the residue was dissolved with *aqua regia* (HCl:$HNO_3$, 3:1 in volume), and analyzed by ICP-AES. Interestingly, in the [Ag/$Al_2O_3$] (12.5% Ag) nanomaterial recovered after reaction, a very similar Ag content of 11.9% was determined, and only a minimal 0.15% of the initial Ag was detected in the reaction mixture. By carrying out elemental analysis, it was possible to identify a 2.1% C in the nanocatalyst after the reaction, and this slight decrease in the Ag content was attributed to the presence of organic residues.

### 2.5. Procedure for the recycling experiments of [Ag/$Al_2O_3$] (12.5% Ag) system

These experiments were carried out by scaling up (x10) the corresponding general reaction procedure described at Experimental Section of the manuscript. A 25 mL vial containing a stirring bar was sequentially charged with *N*-methylphthalimide **1** (2.5 mmol), [Ag/$Al_2O_3$] (12.5% Ag) (6 mol% Ag), *n*-dodecane (200 µL) as an internal standard and 15 mL of previously dried MeOH (dried with 4Å MS during 3 h). Afterwards, the reaction vial was closed with a septum-containing screw cap, perforated with a needle, and introduced into the 300 mL autoclave. Then, the autoclave was closed and pressurized with $H_2$. After three purges, 40 bar of $H_2$ were charged and the autoclave was placed into an aluminium block preheated at 90 ºC on a stirring plate at 750 rpm. After 5 h, the autoclave was cooled down with the help of an ice bath, carefully depressurized, and opened. To quantify the reaction outcome, an aliquot of 50 µL of the reaction mixture was diluted with EtOAc, centrifuged and the liquid fraction was analysed by GC. The reaction mixture was then filtered under vacuum, washed with EtOAc (200 mL) and acetone (50 mL), and dried overnight in an oven at 100 ºC. Finally, a calcination process under air flow was performed at 300 ºC during 3 h, using a heating ramp of 2 ºC/min. At this point, the metal content of the spent catalyst was determined by ICP-AES, dissolving a known quantity of it with *aqua regia* (HCl:$HNO_3$, 3:1 in volume). The procedure was the same during the five catalytic cycles, recalculating the quantity of all the reagents as a function of the recovered solid catalyst (Figure S3).

## 3. COMPLEMENTARY CATALYTIC STUDIES

**Table S1.** Study of the catalytic activity of [M/$Al_2O_3$] (x% M) nanomaterials based on non-noble metals (with M = Fe, Co, Ni and Cu) for the hydrogenation of *N*-methylphthalimide **1**.

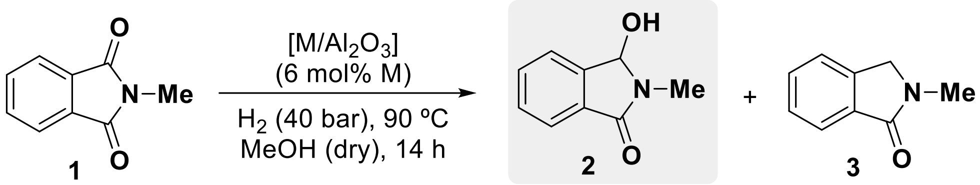


| Entry[a] | [M/$Al_2O_3$][b] | Conv. **1** (%)[c] | **2** (%)[c] | **3** (%)[c] |
|---|---|---|---|---|
| 1 | [Fe/$Al_2O_3$] (2.2% Fe) | <5 | - | - |
| 2 | [Co/$Al_2O_3$] (2.6% Co) | <5 | - | - |
| 3 | [Ni/$Al_2O_3$] (2.2% Ni) | <5 | - | - |
| 4 | [Cu/$Al_2O_3$] (1.8% Cu) | <5 | - | - |

[a]Reaction conditions: *N*-methylphthalimide **1** (0.25 mmol), [M/$Al_2O_3$] (2.2-2.5% of M in the material, 6 mol% of M) previously calcined under air flow at 500 ºC during 3 h with a heating ramp of 2 ºC/min, *n*-dodecane (20 μL) as internal standard and 1 mL of MeOH (previously dried with 4Å MS) at 40 bar of $H_2$ and 90 ºC during 14 h. For more specific information about nanomaterial preparation see section 2.2. [b]The real metal content (wt%) indicated between parenthesis for each nanomaterial was determined by ICP-AES (Table S3). [c]The conversion of **1** and was calculated by GC using *n*-dodecane as an internal standard. Products **2** and **3** were not detected in any case.

**Table S2.** Study of the influence of [Ag/$Al_2O_3$] (4.2% Ag) nanomaterials calcination temperature in the catalytic activity for the hydrogenation of *N*-methylphthalimide **1**.

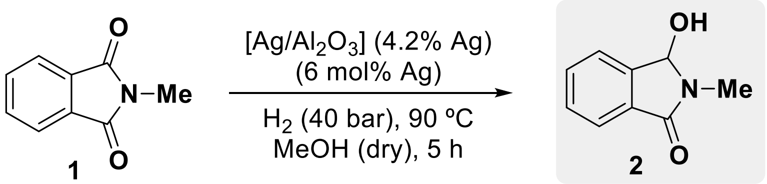


| Entry[a] | Calcination T (ºC) | Conv. **1** (%)[b] | **2** (%)[b] | Sel. **2** (%)[b] |
|---|---|---|---|---|
| 1 | 300 | 78 | 78 | >99 |
| 2 | 400 | 81 | 81 | >99 |
| 3 | 500 | 85 | 85 | >99 |
| 4 | 600 | 89 | 89 | >99 |
| 4 | 700 | 91 | 91 | >99 |

[a]Reaction conditions: *N*-methylphthalimide **1** (0.25 mmol), [Ag/$Al_2O_3$] (4.2 wt% of Ag in the material, 6 mol% of Ag) previously calcined under air flow at different temperatures during 3 h with a heating ramp of 2 ºC/min, *n*-dodecane (20 μL) as internal standard and 1 mL of MeOH (previously dried with 4Å MS) at 40 bar of $H_2$ and 90 ºC during 5 h. For more specific information about nanomaterial preparation see section 2.2. [b]The conversion of **1**, the yield of 2 and its selectivity were calculated by GC using *n*-dodecane as an internal standard.

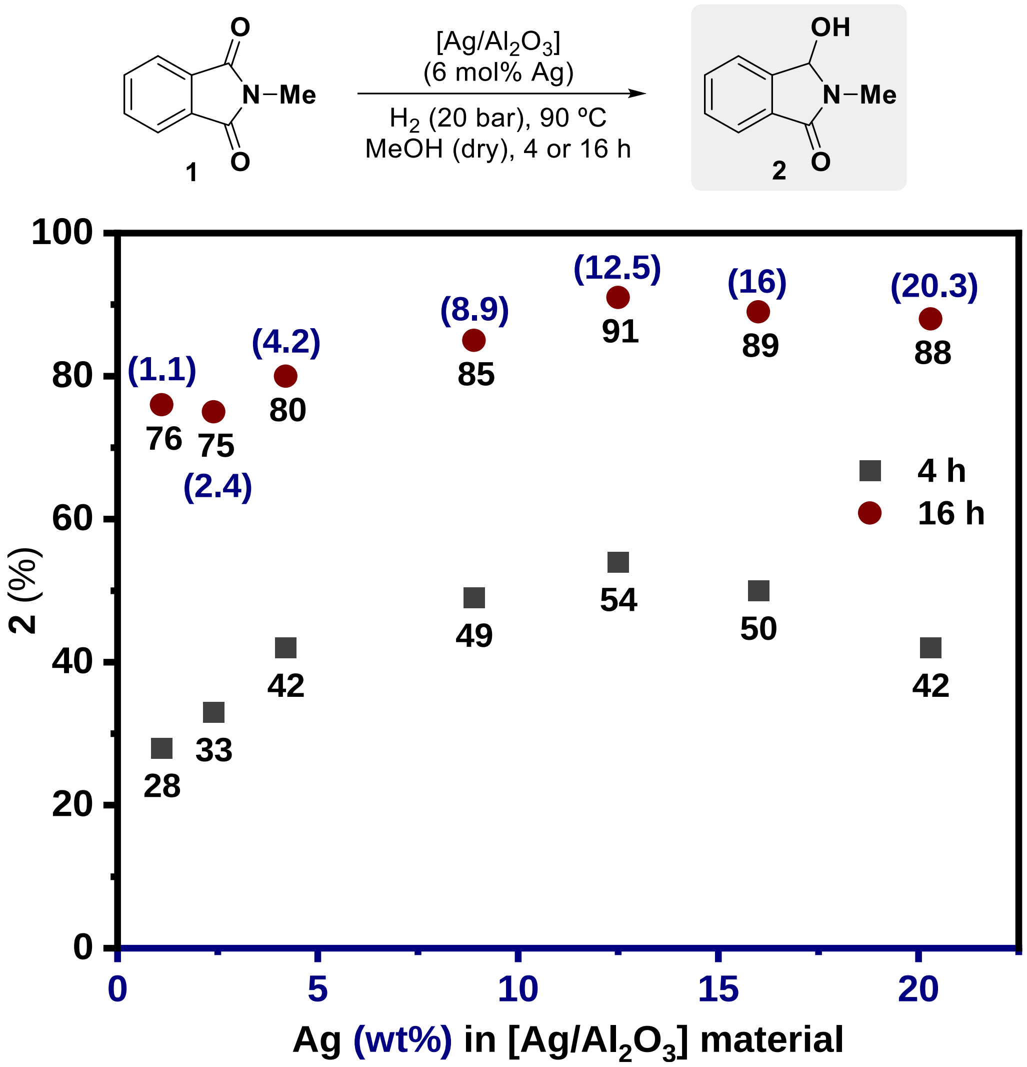


**Figure S1.** Study of the influence of the Ag (wt%) content in [Ag/$Al_2O_3$] (x% Ag) materials (where x = 1.1, 2.4, 4.2, 8.9, 12.4, 16 and 20.3) in the catalytic activity for the hydrogenation of *N*-methylphthalimide **1**. The real Ag content (wt%) for each nanomaterial was determined by ICP-AES (Table S5). For more specific information about nanomaterial preparation see section 2.2.1. Reaction conditions: *N*-Methylphthalimide **1** (0.25 mmol), [Ag/$Al_2O_3$] material (1.1-20.3% of Ag in the material, 6 mol% of Ag) previously calcinated under air flow at 700 ºC during 3 h, with a heating ramp of 2 ºC/min, *n*-dodecane as internal standard (20 μL) and 1 mL of MeOH (previously dried with 4Å MS) at 20 bar of $H_2$ and 90 ºC during 4 (gray squares) and 16 h (red circles). Conversion of **1** and yield of product **2** were determined by GC using *n*-dodecane as an internal standard.

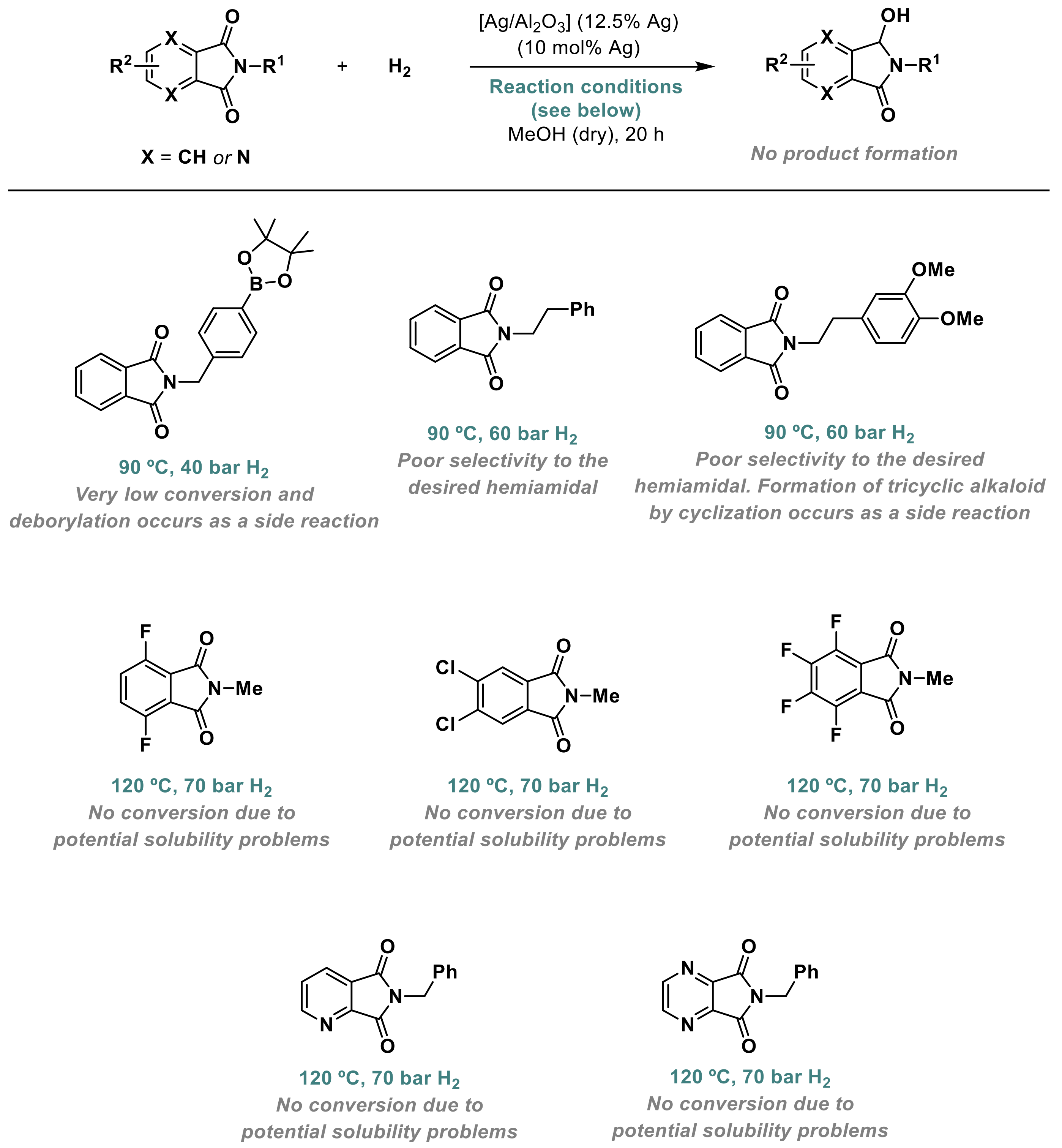


**Scheme S1.** Limitations of the protocol for the [$Ag/Al_2O_3$]-catalyzed selective monohydrogenation of phthalimides. Standard reaction conditions: phthalimide (0.25 mmol), [$Ag/Al_2O_3$] material (12.5 wt% Ag in the material, 10 mol% Ag), 1 mL of MeOH (previously dried with 4 Å MS), $H_2$ (40-70 bar) at 90-120 ºC during 20 h.

## 4. [$Ag/Al_2O_3$] SYSTEM HETEROGENEITY EXPERIMENTS

### 4.1. Filtration tests

These experiments were performed following the general procedure described in section 2.4 of the Supporting Information.

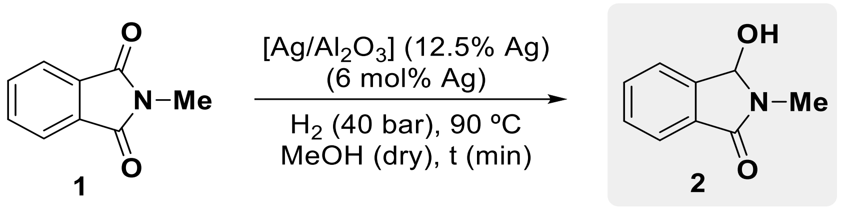


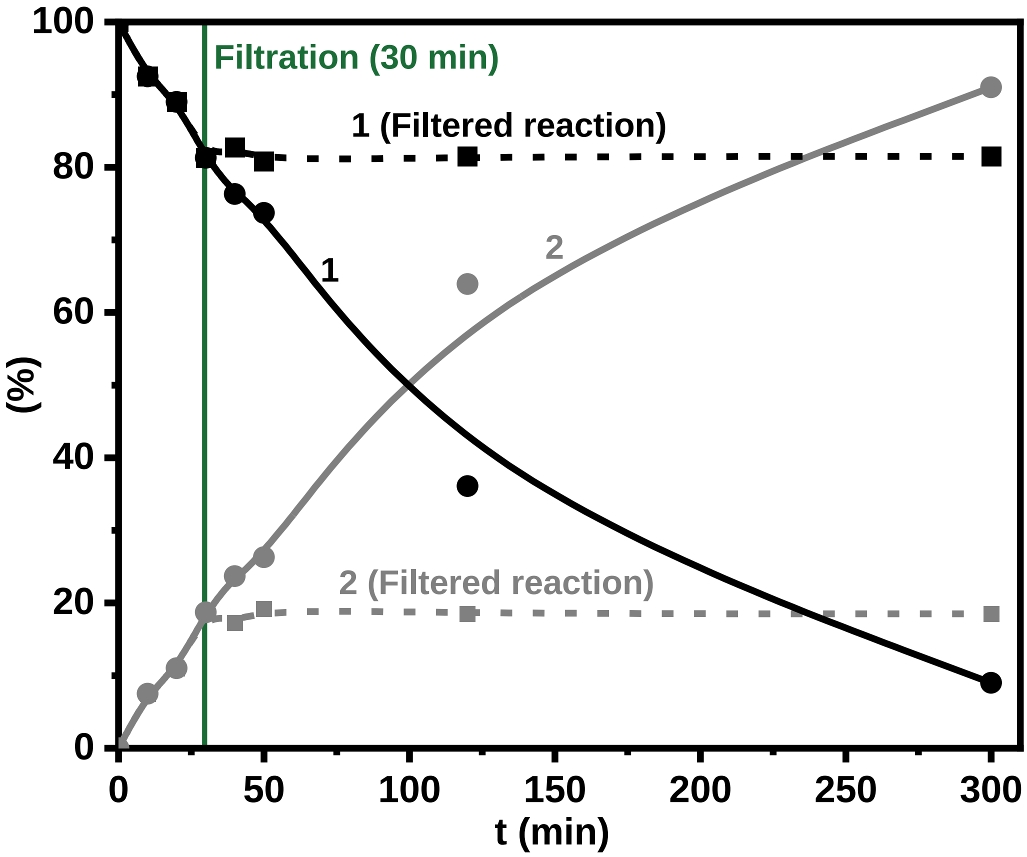


**Figure S2.** Filtration test for the catalytic hydrogenation of *N*-methylphthalimide **1** in the presence of [$Ag/Al_2O_3$] (12.5% Ag) nanomaterial. Circles refer to not filtered reaction. Squares refer to filtered reaction. The vertical green line indicates the filtration time at 30 min. Reaction conditions: *N*-methylphthalimide **1** (0.75 mmol), [$Ag/Al_2O_3$] material (12.5% of Ag in the material, 6 mol% Ag), *n*-dodecane (60 µL) and dry MeOH (previously dried with 4 Å MS during 3 h, 3 mL) at 40 bar of $H_2$ and 90 ºC. Conversion of **1** and yield of product **2** were determined by GC using *n*-dodecane as an internal standard.

### 4.2. Recycling studies

These experiments were performed following the general procedure described in section 2.5 of the Supporting Information.

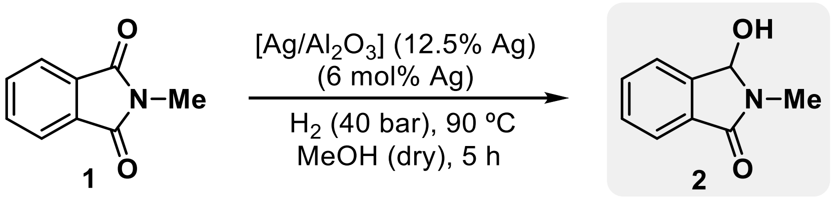


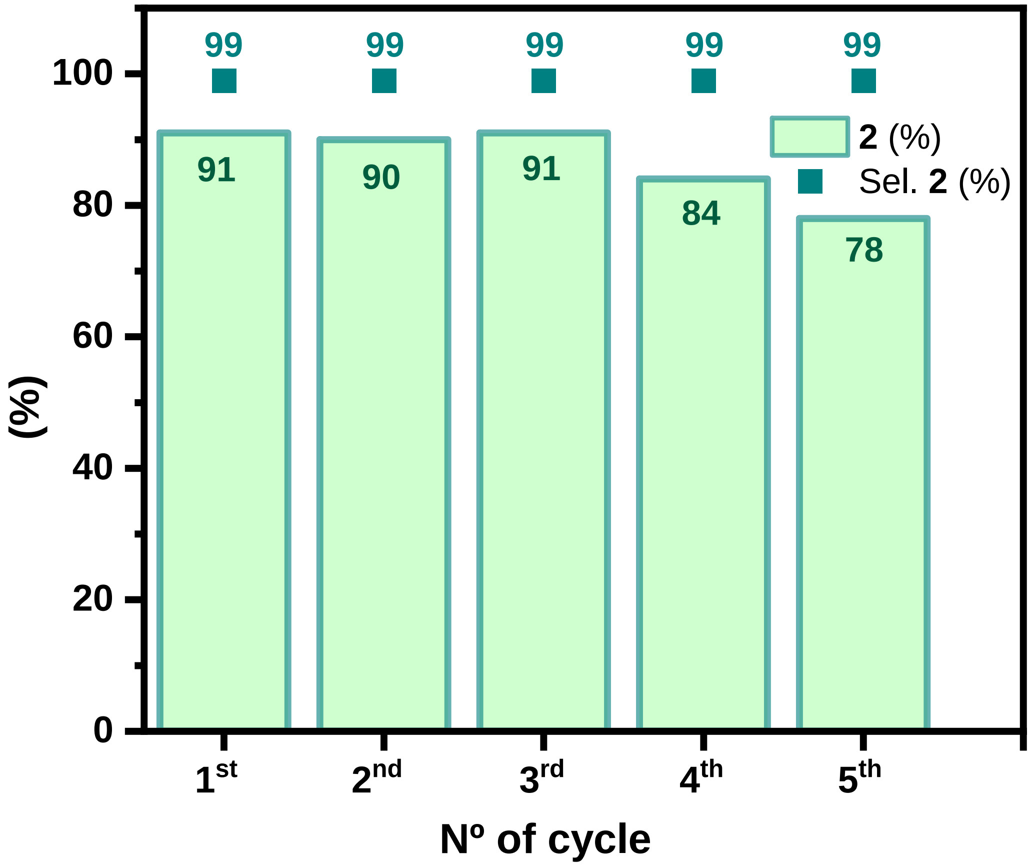


**Figure S3.** Reusability study for the monohydrogenation of **1** to **2** in the presence of [Ag/$Al_2O_3$] (12.5% Ag) nanocatalyst. Standard reaction conditions: *N*-methylphthalimide **1** (2.5 mmol), [Ag/$Al_2O_3$] material (12.5% of Ag in the material, 6 mol% of Ag), *n*-dodecane (500 μL), 10 mL of MeOH (previously dried with 4 Å MS), 40 bar of $H_2$ at 90 ºC during 5 h. After being used in a successive reaction cycle, the material was filtered and calcined under air flow at 300 ºC for 3 h. Yield and selectivity of **2** were determined by GC using *n*-dodecane as an internal standard.

## 5. CHARACTERIZATION OF THE MATERIALS

### 5.1. Inductively Coupled Plasma-Atomic Emission Spectroscopy (ICP-AES)

**Table S3.** Metal content in [M/$Al_2O_3$] nanomaterials

| Entry[a] | [M/$Al_2O_3$] | [Theoretical metal content] | [Real metal content][b] |
|---|---|---|---|
| 1 | [Ag/$Al_2O_3$] | 4.1 | 4.2 |
| 2 | [Pd/$Al_2O_3$] | 4.1 | 3.6 |
| 3 | [Pt/$Al_2O_3$] | 7.2 | 6.7 |
| 4 | [Fe/$Al_2O_3$] | 2.2 | 2.2 |
| 5 | [Co/$Al_2O_3$] | 2.3 | 2.6 |
| 6 | [Ni/$Al_2O_3$] | 2.3 | 2.2 |
| 7 | [Cu/$Al_2O_3$] | 2.5 | 1.8 |

[a][M/$Al_2O_3$] materials were prepared with 0.4 mmol of the corresponding metal precursor and 1 g of $Al_2O_3$ support, calcination at 500 $^oC$ under air flow during 3 h, with a heating ramp of 2 $^oC$/min during 3 h. For more specific information about nanomaterials preparation see section 2.2. [b]Real metal content (wt%) was determined by ICP-AES.

**Table S4.** Metal content in [Ag/support] nanomaterials

| Entry[a] | [Ag/Support] | [Theoretical metal content] | [Real metal content][b] |
|---|---|---|---|
| 1 | [Ag/$Al_2O_3$] | 4.1 | 4.2 |
| 2 | [Ag/$TiO_2$] | 4.1 | 4.8 |
| 3 | [Ag/$SiO_2$] | 4.1 | 4 |
| 4 | [Ag/$Nb_2O_5$] | 4.1 | 4 |
| 5 | [Ag/$ZrO_2$] | 4.1 | 4.4 |
| 6 | [Ag/ZnO] | 4.1 | 5 |
| 7 | [Ag/$CeO_2$] | 4.1 | 4.9 |
| 8 | [Ag/HAP] | 4.1 | 5.1 |

[a][Ag/support] materials were prepared with 0.4 mmol of [Ag(acac)] and 1 g of the corresponding support, calcination at 500 $^oC$ under air flow during 3 h, with a heating ramp of 2 $^oC$/min during 3 h. For more specific information about nanomaterials preparation see section 2.2. [b]Real metal content (wt%) was determined by ICP-AES. HAP = hydroxyapatite.

**Table S5.** Metal content in [Ag/$Al_2O_3$] nanomaterials

| Entry[a] | Mmol of [Ag(acac)] | [Theoretical metal content] | [Real metal content][b] |
|---|---|---|---|
| 1 | 0.1 | 1.1 | 1.1 |
| 2 | 0.2 | 2.1 | 2.4 |
| 3 | 0.4 | 4.1 | 4.2 |
| 4 | 0.8 | 7.9 | 8.9 |
| 5 | 1.2 | 11.4 | 12.5 |
| 6 | 1.6 | 14.7 | 16 |
| 7 | 2 | 17.7 | 20.3 |

[a][Ag/$Al_2O_3$] materials were prepared by using the indicated amount of [Ag(acac)] precursor and 1 g of $Al_2O_3$ support, calcination at 700 $^oC$ under air flow during 3 h, with a heating ramp of 2 $^oC$/min during 3 h. For more specific information about nanomaterials preparation see section 2.2. [b]Real metal content (wt%) was determined by ICP-AES.

## 5.2. X-Ray Powder Diffraction (XRPD)

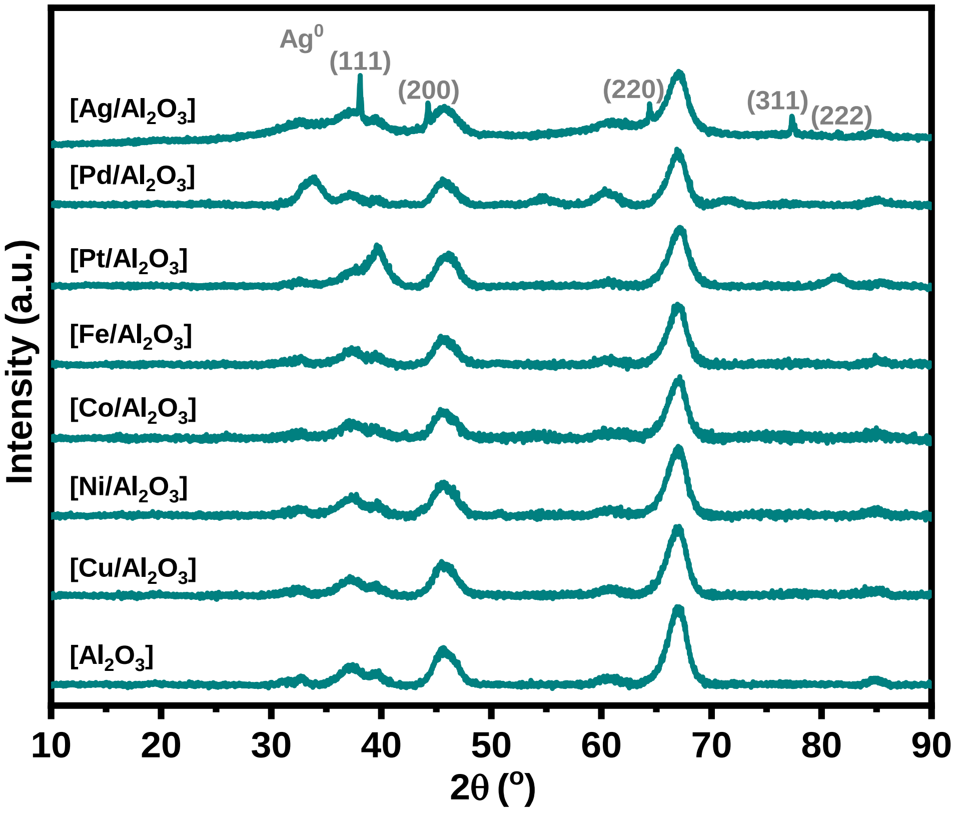


**Figure S4.** XRPD pattern of freshly prepared [M/$Al_2O_3$] (1.8-6.7 wt% M) materials (M = Ag, Pd, Pt, Fe, Co, Ni and Cu). $Al_2O_3$ support has been used for comparison. It is detected the presence of crystalline $Ag^0$ nanoparticles with face-centered cubic structure and crystallographic diffraction planes with Miller indexes of (111), (200), (220), (311) and (222) (shown between parenthesis, gray squares, JCPDS No. 04-0783).[3-5]

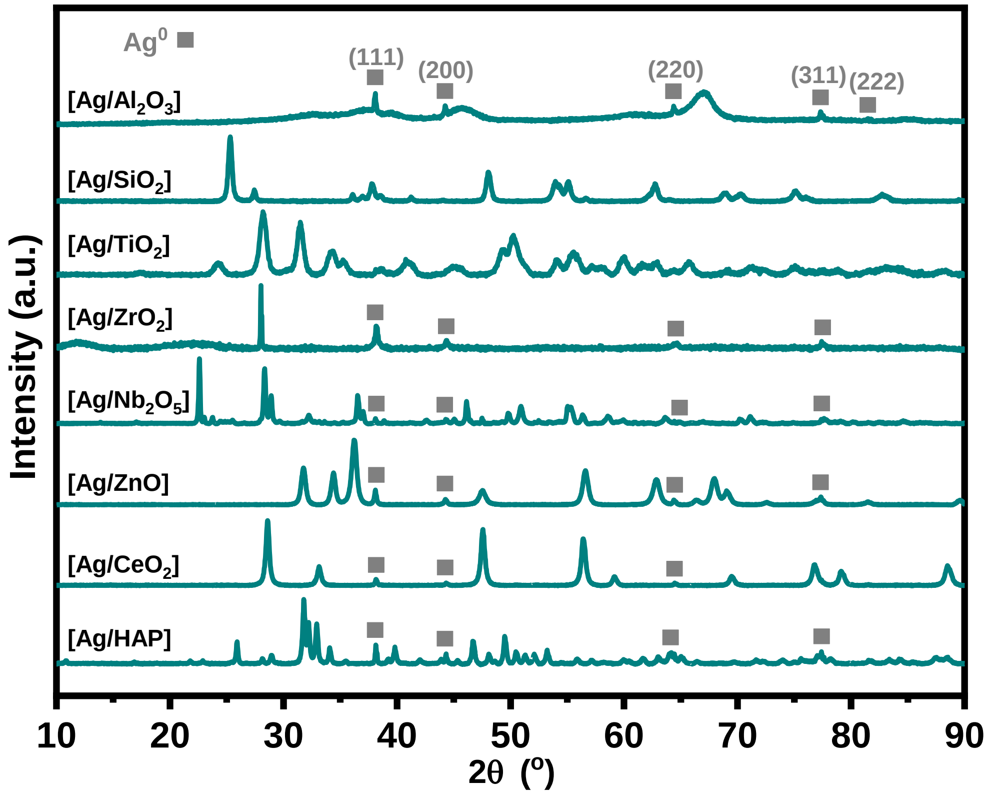


**Figure S5.** XRPD pattern of freshly prepared [Ag/support] (4-5.1 wt% M) materials (support = $Al_2O_3$, $SiO_2$, $TiO_2$, $ZrO_2$, $Nb_2O_5$, ZnO, $CeO_2$ and HAP). It is detected the presence of crystalline $Ag^0$ nanoparticles with face-centered cubic structure and crystallographic diffraction planes with Miller indexes of (111), (200), (220), (311) and (222) (gray squares, JCPDS No. 04-0783).[3-5]

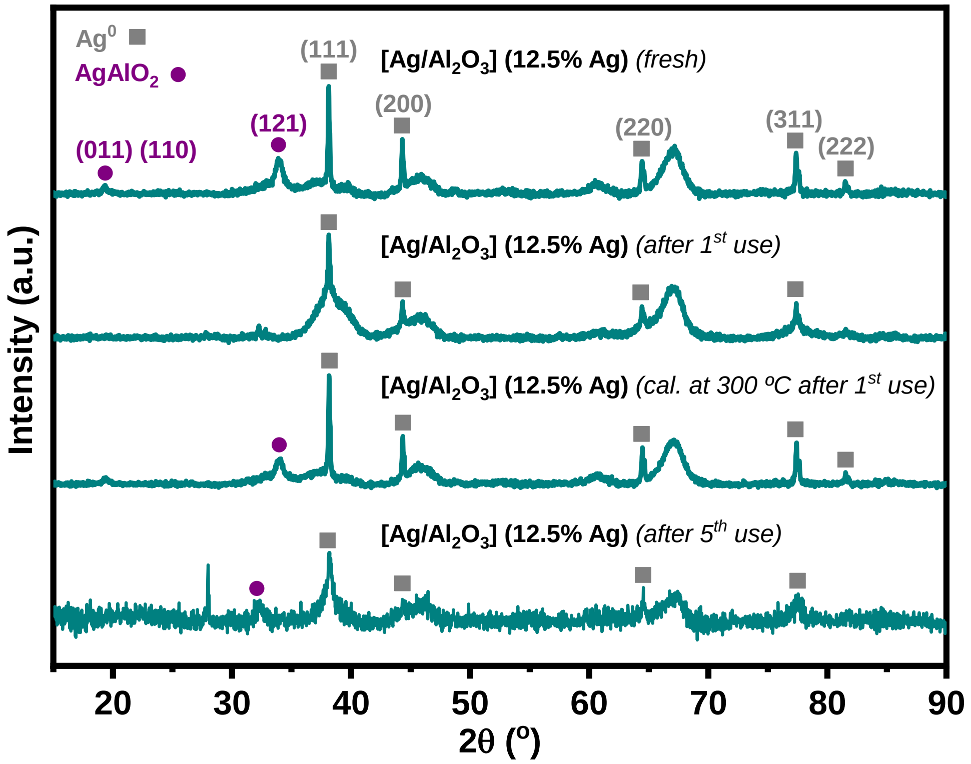


**Figure S6.** XRPD pattern of freshly prepared [$Ag/Al_2O_3$] (12.5% Ag) material, recovered material after 1st use as catalyst for the monohydrogenation of *N*-methylphthalimide **1**, recovered material after 1st use as catalyst and then calcined at 300 ºC during 3 h, recovered material after 5th use as catalyst. It is detected the presence of crystalline $Ag^0$ nanoparticles with face-centered cubic structure and crystallographic diffraction planes with Miller indexes of (111), (200), (220), (311) and (222) (gray squares, JCPDS No. 04-0783) and the presence of $AgAlO_2$ species with delafossite structure and crystallographic diffraction planes with Miller indexes of (011), (110) and (121) (purple circles, JCPDS No. 21-1070).[6]

**5.3. High-Angle Annular Dark-Field High-Resolution Scanning Transmission Electron Microscopy (HAADF-HRSTEM) and Scanning-Transmission Energy-Dispersive X-Ray Spectroscopy (STEM-XEDS)**

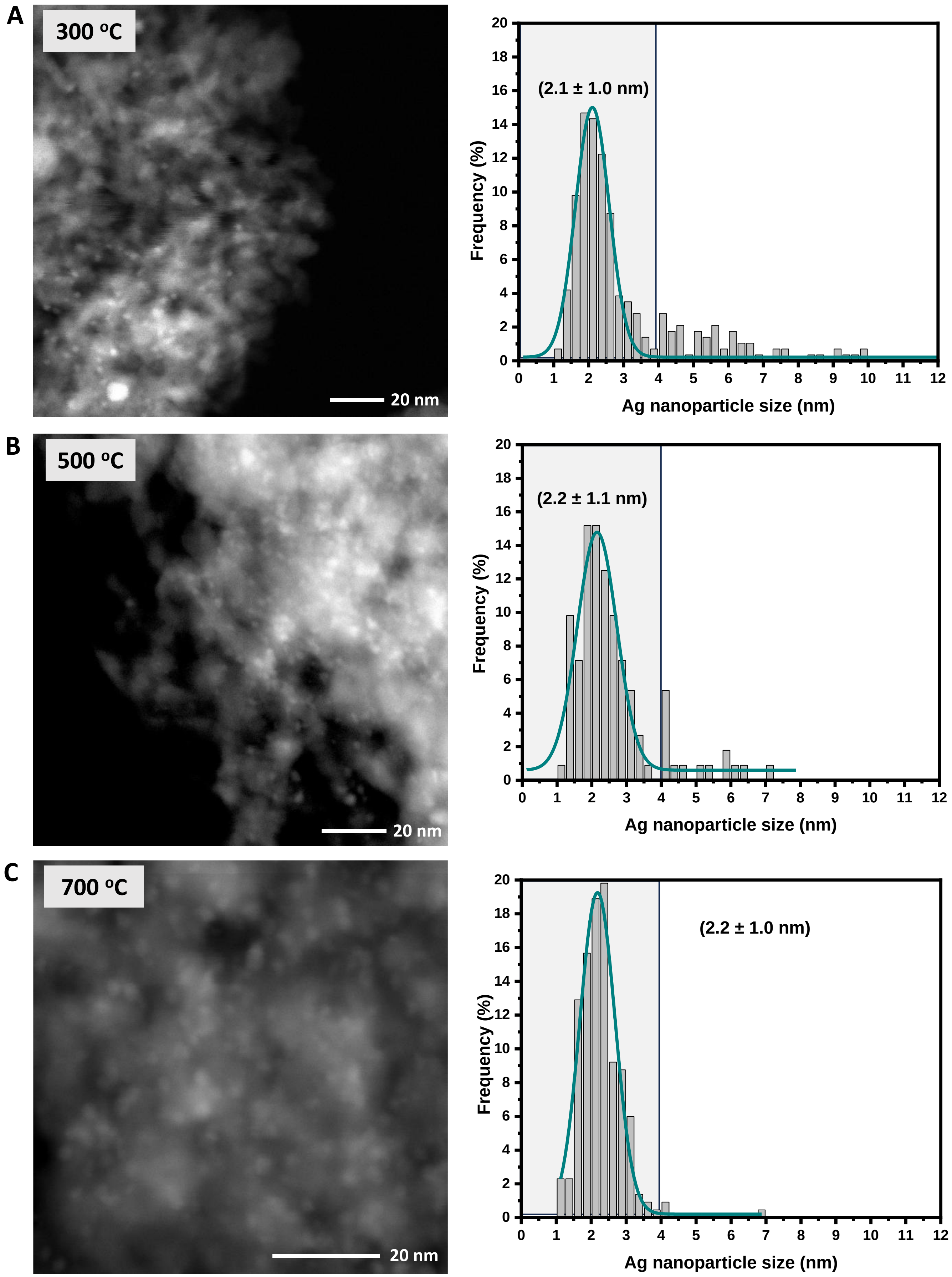


**Figure S7.** HAADF-HRSTEM analysis of [Ag/$Al_2O_3$] (4.2% Ag) materials calcined at 300 ºC (**A**), 500 ºC (**B**) and 700 ºC (**C**). In all cases, the resulting histogram corresponding to the analysis of more than 200 different silver nanoparticles are given. The average nanoparticle size was obtained after adjusting the sizes to a Gaussian distribution.

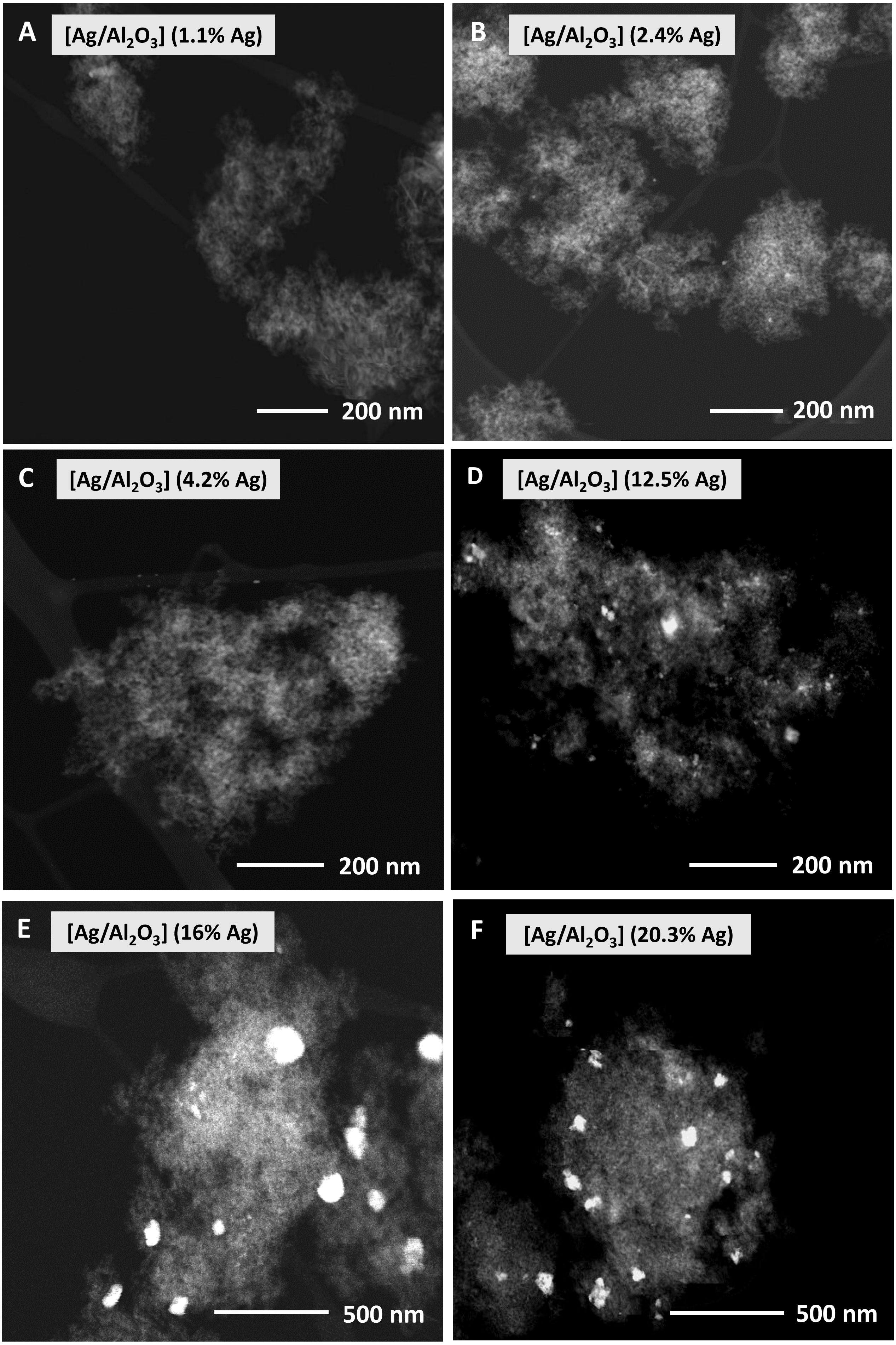


**Figure S8.** HAADF-HRSTEM analysis at low magnifications (200 to 500 nm) of [Ag/$Al_2O_3$] (x%) materials (x = 1.1, 2.4, 4.2,12.5, 16, 20.3) (**A-F**, respectively).

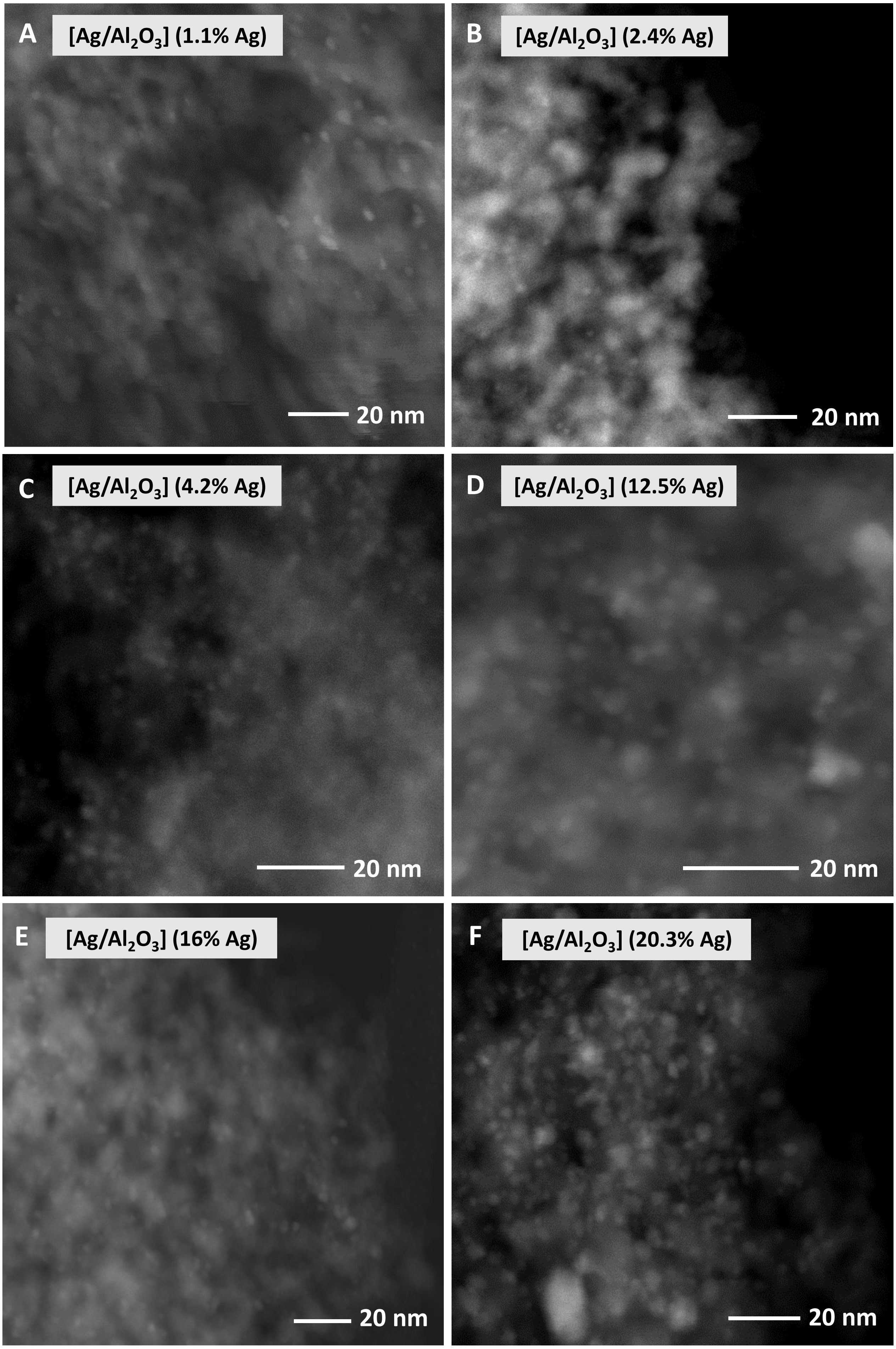


**Figure S9.** HAADF-HRSTEM analysis at high magnifications (20 nm) of [Ag/$Al_2O_3$] (x%) materials (x = 1.1, 2.4, 4.2,12.5, 16, 20.3) (**A-F**, respectively).

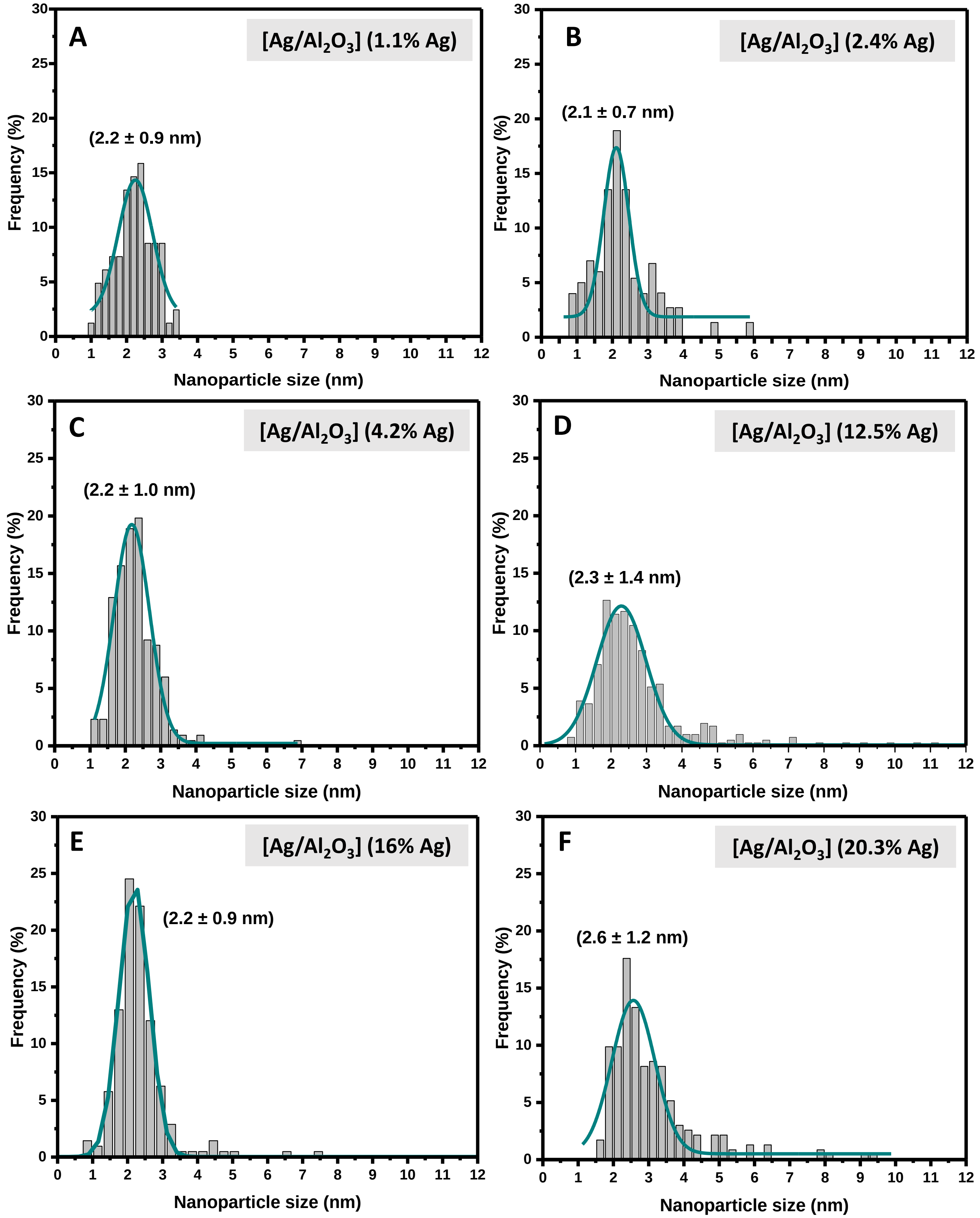


**Figure S10.** Silver nanoparticle size analysis of [Ag/$Al_2O_3$] (x%) materials (x = 1.1, 2.4, 4.2, 12.5, 16 and 20.3) (**A**-**F**, respectively). In all cases, the resulting histogram corresponding to the analysis by HAADF-HRSTEM of more than 200 different silver nanoparticles are given. The average nanoparticle size was obtained after adjusting the sizes to a Gaussian distribution.

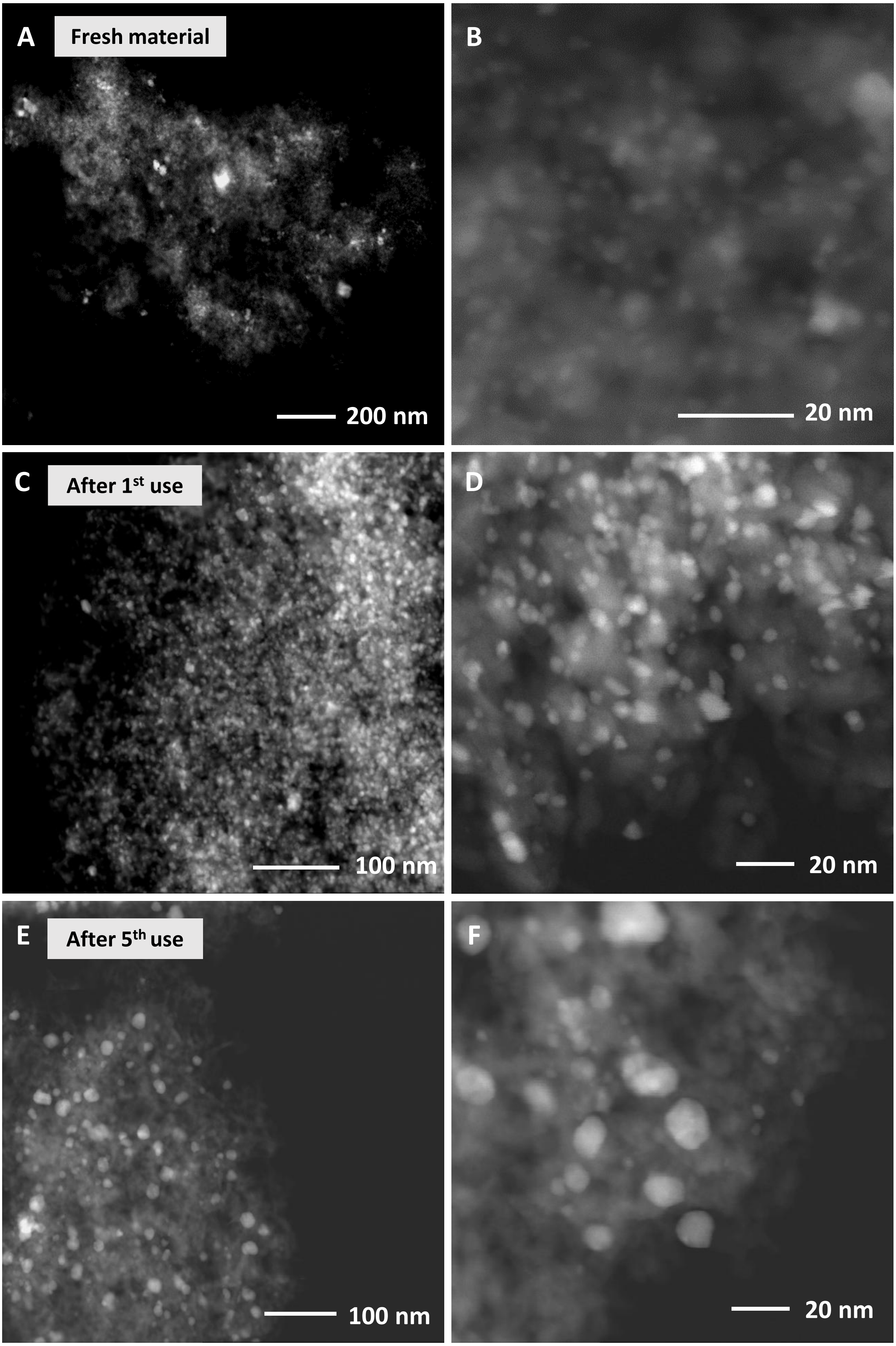


**Figure S11.** HAADF-HRSTEM image of fresh [$Ag/Al_2O_3$] (12.5% Ag) nanomaterial (**A** and **B**), and the recovered material after 1$^{st}$ use (**C** and **D**) and 5$^{th}$ use (**E** and **F**) as catalyst for the monohydrogenation of *N*-methylphthalimide **1**.

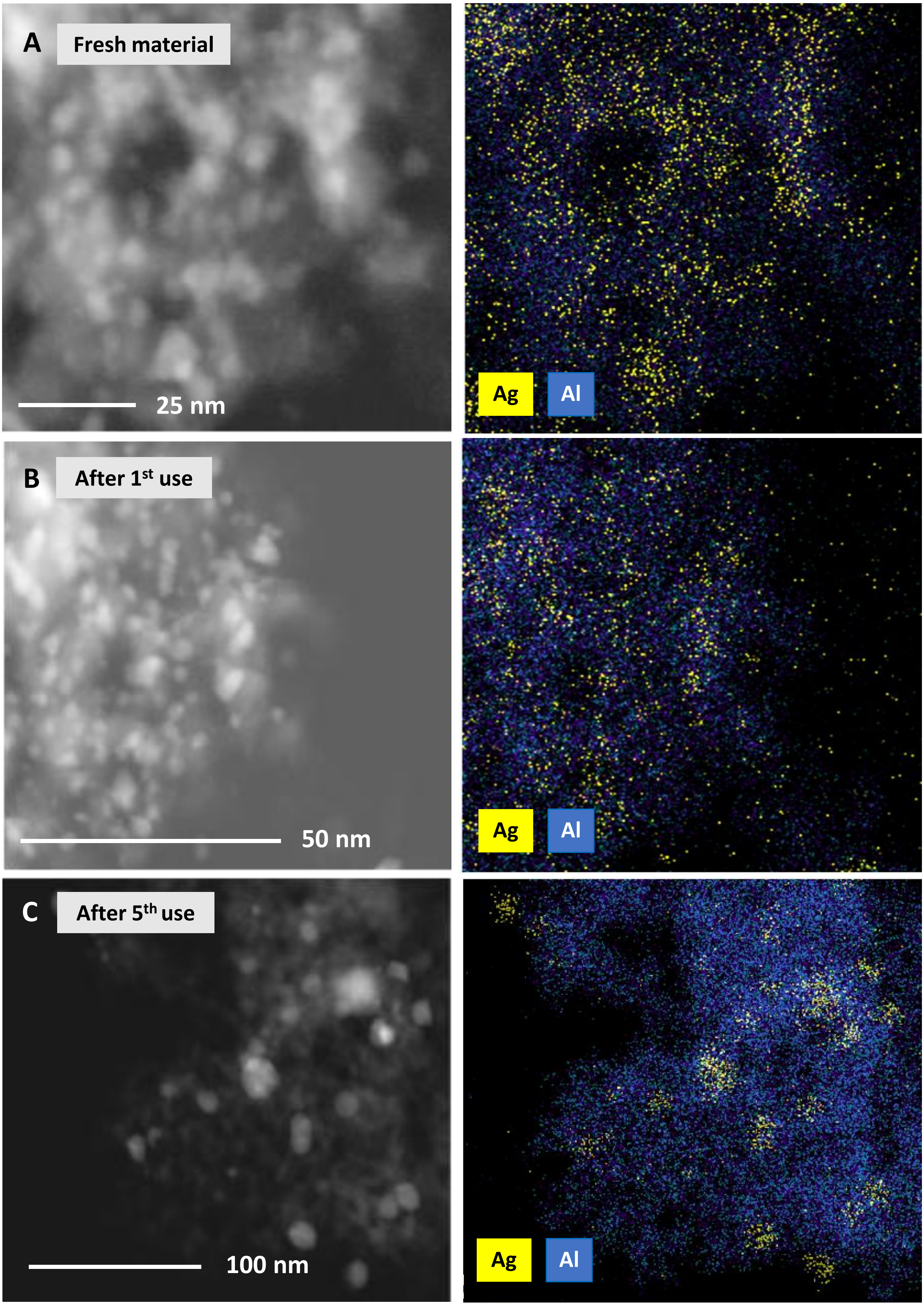


**Figure S12.** STEM-XEDS analysis of fresh [Ag/$Al_2O_3$] (12.5% Ag) nanomaterial (**A**), recovered material after 1st use (**B**) and 5th use (**C**) as catalyst for the monohydrogenation of *N*-methylphthalimide **1**.

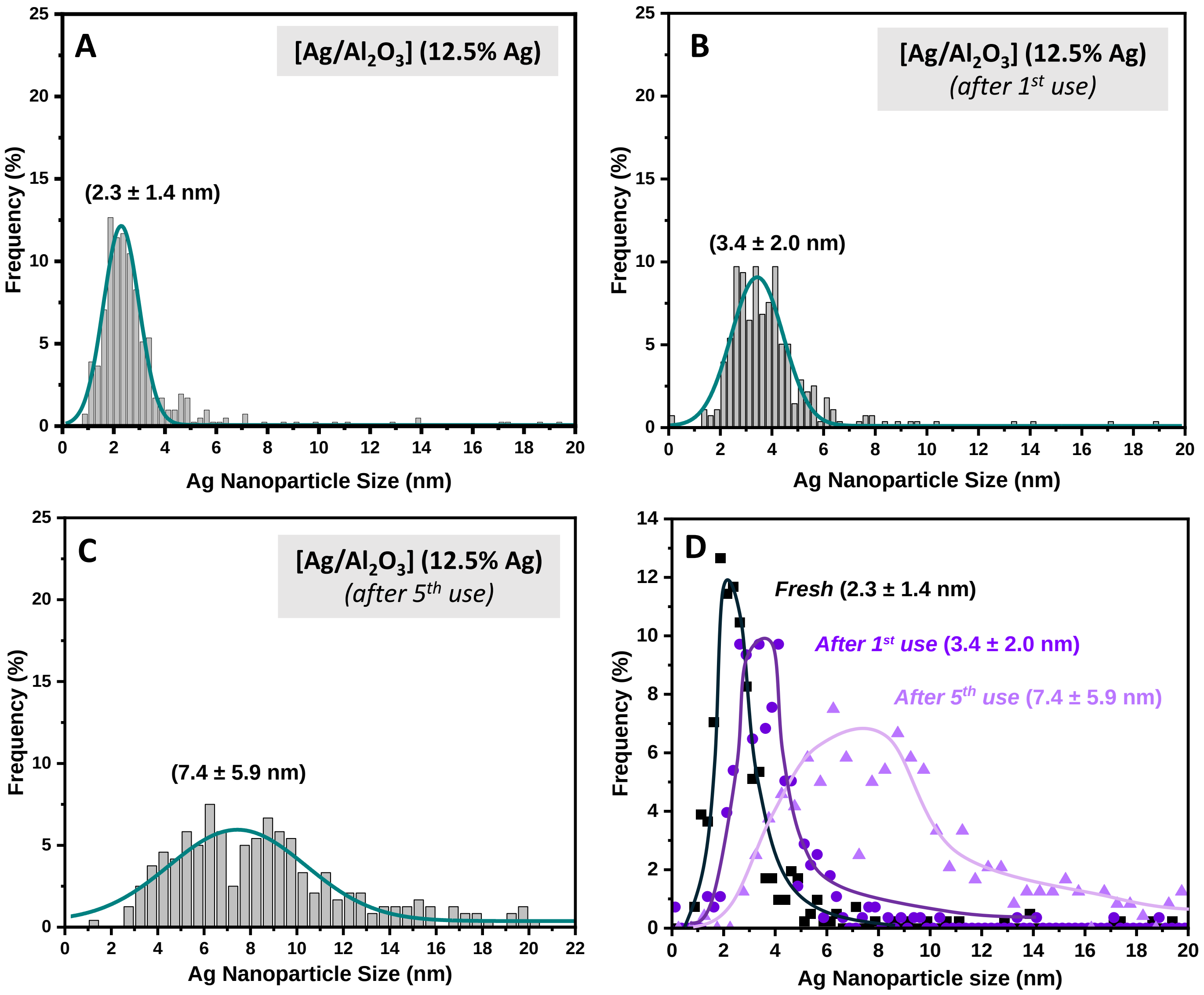


**Figure S13.** Nanoparticle size analysis of fresh [$Ag/Al_2O_3$] (12.5% Ag) nanomaterial (**A**), recovered material after 1$^{st}$ use (**B**) and after 5$^{th}$ use (**C**) as catalyst for the monohydrogenation reaction of *N*-methylphthalimide **1** and comparison of the average of Ag nanoparticle size (**D**) determined by HAADF-HRSTEM analysis. In all cases, the resulting histogram corresponding to the analysis by HAADF-HRSTEM of more than 200 different silver nanoparticles are given.

### 5.4. Specific surface area and pore diameter

**Table S6.** Textural properties of selected [$Ag/Al_2O_3$] (4.2% Ag) materials calcined at different temperatures.

| Entry | T (ºC) | Specific Surface Area ($m^2/g$)[a] | Average Pore Diameter (Å)[b] |
|---|---|---|---|
| 1 | 300 | 170.5 | 105.1 |
| 2 | 500 | 174.7 | 109.7 |
| 3 | 700 | 168.0 | 106.8 |
| 4[c] | 500-[$Al_2O_3$] | 179.4 | 119.8 |

[a]Experimental values calculated from $N_2$ adsorption isotherms (Brunauer-Emmet-Teller, BET method). [b]Experimental values of Barrett-Joiner-Halenda (BJH) desorption average pore diameter are given. [c]$\gamma$-$Al_2O_3$ calcined at 500 ºC during 3 h.

**5.5. UV-Vis Diffuse Reflectance Spectroscopy (UV-Vis DRS)**

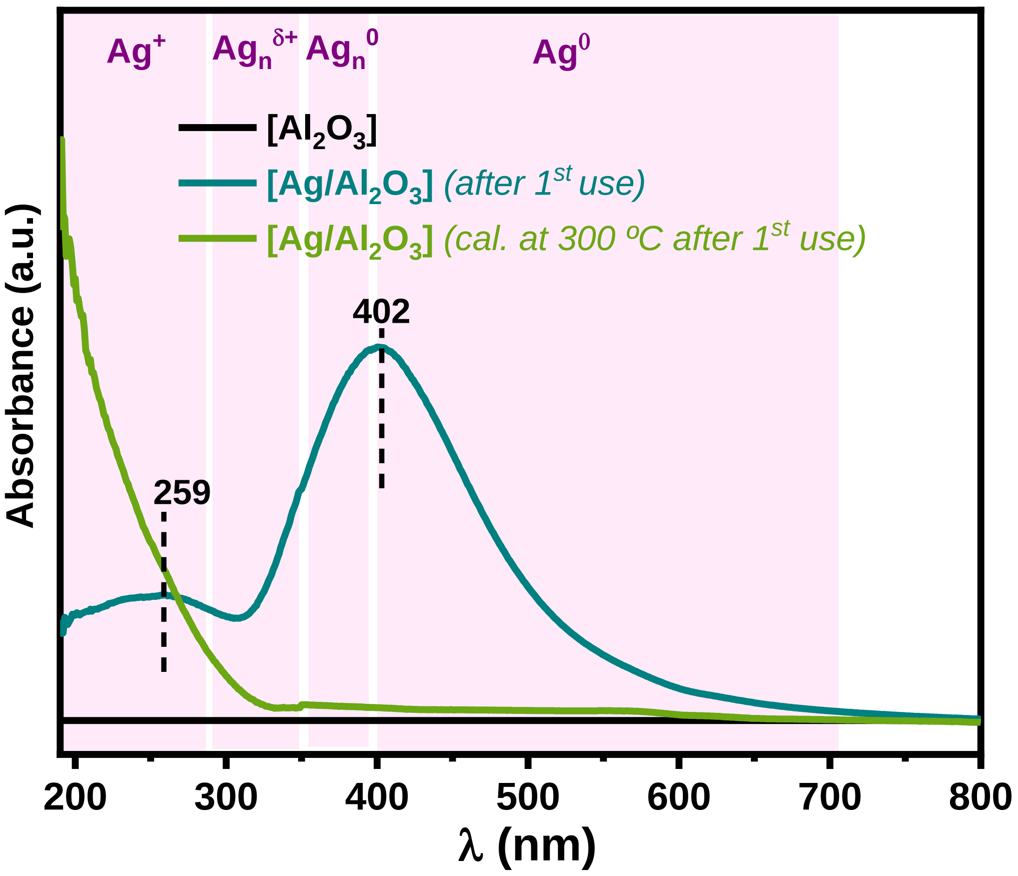


**Figure S14.** UV-Vis DRS spectra of [Ag/$Al_2O_3$] (12.5% Ag) material recovered after 1st use as catalyst in the hydrogenation of *N*-methylphthalimide **1**, the same solid recovered but being calcined under air flow at 300 ºC for 3 h before analysis and $Al_2O_3$ support.

### 5.6. X-Ray Photoelectron Spectroscopy

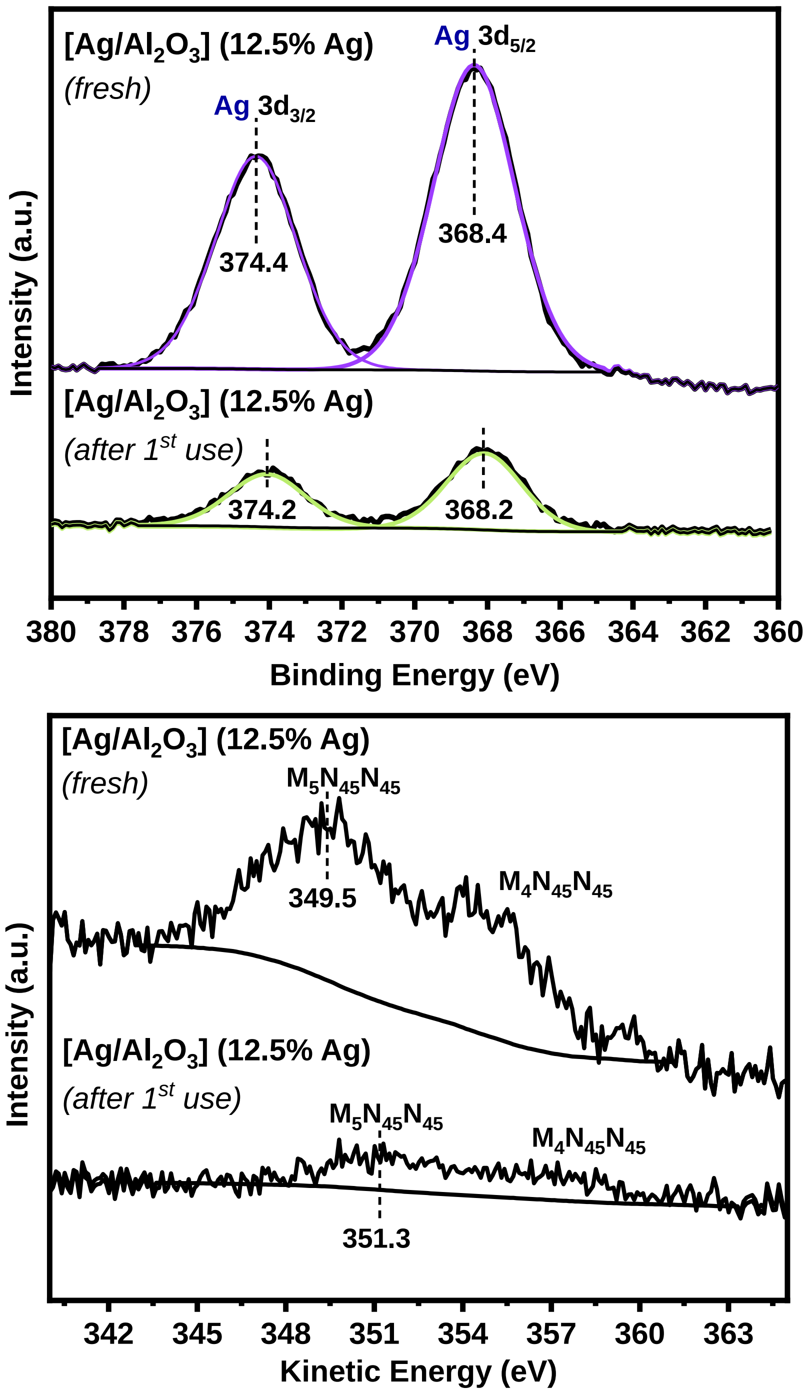


**Figure S15.** XPS spectra of the Ag 3d region (up) and the Ag $M_{4,5}N_{45}N_{45}$ Auger zone (bottom) for fresh [Ag/$Al_2O_3$] (12.5% Ag) nanomaterial and for the material recovered after 1st use as catalyst in the hydrogenation of *N*-methylphthalimide **1**.

**Table S7.** Ag $3d_{5/2}$ Binding energy (BE), kinetic energy (KE ± standard deviation) of Ag $M_5N_{45}N_{45}$ Auger, and the modified Auger parameter ($\alpha'$ = BE + KE) for fresh [Ag/$Al_2O_3$] (12.5% Ag) nanomaterial and for the material recovered after 1st use as catalyst in the hydrogenation of *N*-methylphthalimide **1**. Corrected to the Al 2p component.

| Entry | Material version | BE (eV) Ag $3d_{5/2}$ | KE (eV) Ag $M_5N_{45}N_{45}$ | $\alpha'$ (eV) (BE + KE) | Specie |
|---|---|---|---|---|---|
| 1 | Fresh | 368.4 | 349.5 ± 0.2 | 717.9 ± 0.2 | $Ag^+$ |
| 2 | After reaction | 368.2 | 351.3 ± 0.2 | 719.5 ± 0.2 | $Ag^o$ |

### 5.7. $H_2$-Temperature Programmed Reduction ($H_2$-TPR)

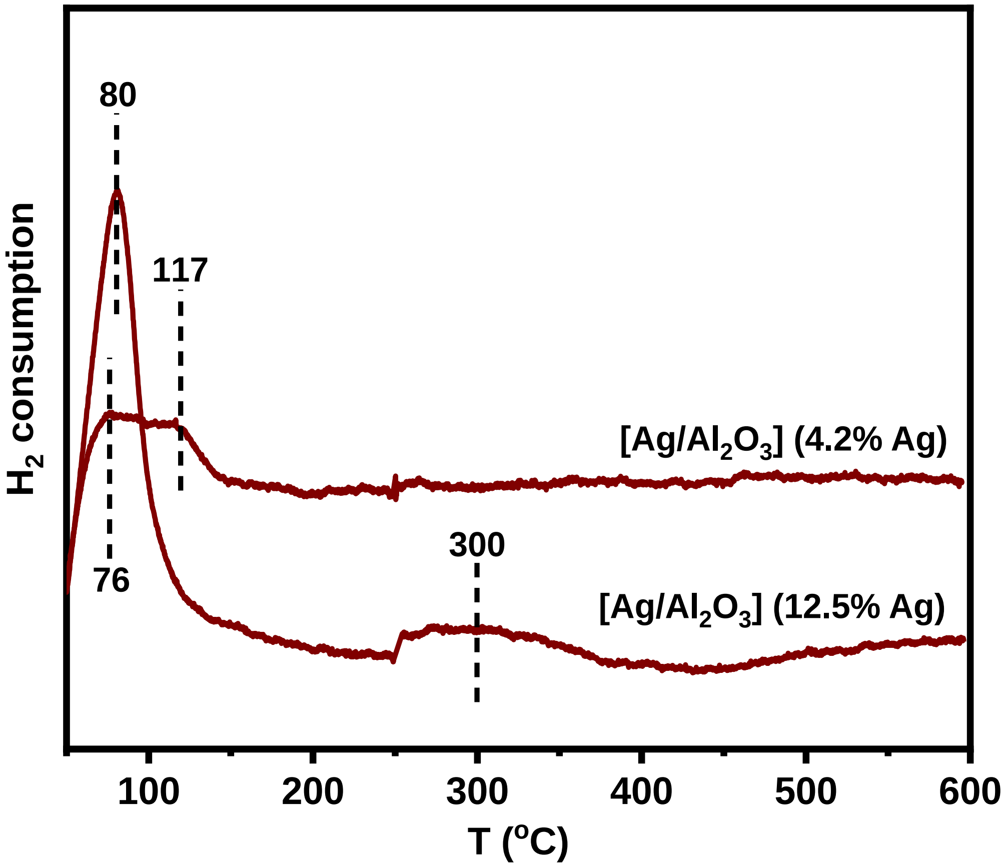


**Figure S16.** $H_2$ TPR-profiles of freshly prepared [Ag/$Al_2O_3$] (4.2% Ag) and [Ag/$Al_2O_3$] (12.5% Ag) nanomaterials.

## 6. CHARACTERIZATION DATA OF THE ISOLATED ORGANIC COMPOUNDS

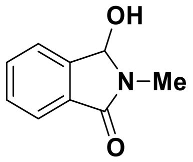


**3-Hydroxy-2-methylisoindolin-1-one (2).**[7] Isolated yield: 91%. GC-MS (m/z, $M^{+\cdot}$ 163), major peaks found: 163 (100%), 146 (32%), 131 (28%), 77 (23%). $^{1}$H NMR (400 MHz, $CDCl_3$) *δ*: 7.65 – 7.45 (m, 3H), 7.40 (t, *J* = 7.4, 1H), 5.59 (s, 1H), 3.82 (bs, 1H), 2.90 (s, 3H). $^{13}$C NMR (100 MHz, MeOD) *δ*: 169.4 (C=O), 145.8 (C), 133.4 (CH), 132.7 (C), 130.7 (CH), 124.5 (CH), 123.6 (CH), 84.3 (CH), 26.3 ($CH_3$). The NMR spectrum is consistent with the reported data.

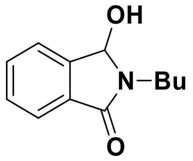


**2-Butyl-3-hydroxyisoindolin-1-one (6).**[7] Isolated yield: 78%. GC-MS (m/z, $M^{+\cdot}$ 205), major peaks found: 205 (39%), 162 (34%), 133 (100%), 105 (26%), 77 (25%), 51 (20%), 28 (18%). $^{1}$H NMR (300 MHz, MeOD) *δ*: 7.72 (dt, *J* = 7.4, 0.9, 1H), 7.66 – 7.60 (m, 2H), 7.57 – 7.49 (m, 1H), 5.86 (s, 1H), 3.78 – 3.63 (m, 1H), 3.44 (ddd, *J* = 13.9, 8.0, 6.0, 1H), 1.80 – 1.60 (m, 2H), 1.48 – 1.32 (m, 2H), 0.99 (t, *J* = 7.3, 3H). $^{13}$C NMR (75 MHz, MeOD) *δ*: 169.3 (C=O), 146.1 (C), 133.4 (CH), 132.9 (C), 130.7 (CH), 124.5 (CH), 123.7 (CH), 82.9 (CH), 40.2 ($CH_2$), 31.5 ($CH_2$), 21.3 ($CH_2$), 14.1 ($CH_3$).

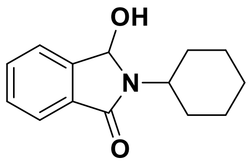


**2-Cyclohexyl-3-hydroxyisoindolin-1-one (7).** Isolated yield: 67%. GC-MS (m/z, $M^{+\cdot}$ 231), major peaks found: 231 (3%), 215 (18%), 193 (3%), 172 (16%), 134 (100%), 105 (54%), 77 (49%), 56 (50%), 28 (16%). $^{1}$H NMR (401 MHz, MeOD) *δ*: 7.70 (d, *J* = 7.5, 1H), 7.66 – 7.57 (m, 2H), 7.52 (td, *J* = 7.3, 1.4, 1H), 5.97 (s, 1H), 3.98 – 3.90 (m, 1H), 2.06 – 1.84 (m, 6H), 1.74 – 1.71 (m, 1H), 1.51 – 1.09 (m, 3H). $^{13}$C NMR (75 MHz, MeOD) *δ*: 169.1 (C=O), 146.3 (C), 133.4 (CH), 133.0 (C), 130.6 (CH), 124.3 (CH), 123.6 (CH), 82.7 (CH), 53.7 (CH), 33.0 ($CH_2$), 31.5 ($CH_2$), 27.2 ($CH_2$), 27.2 ($CH_2$), 26.7 ($CH_2$). HRMS (ESI+) [M+H$^+$; calculated for $C_{14}H_{18}NO_2$: 232.1332] found m/z 232.1331.

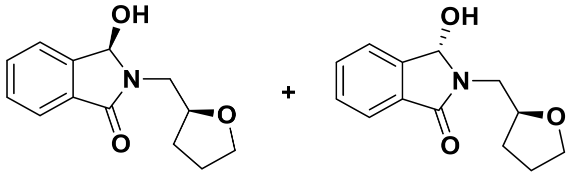


**3-Hydroxy-2-((tetrahydrofuran-2-yl)methyl)isoindolin-1-one (8).** Isolated yield: 92%. Both compounds were isolated as a racemic diastereomers mixture. Two different peaks with the same fragmentations were detected by GC-MS (m/z, $M^{+\cdot}$ 233), major peaks found: 233 (7%), 215 (7%), 189 (15%), 163 (52%), 105 (100%), 89 (33%), 83 (10%), 77 (21%), 71 (73%), 43 (32%), 27 (15%). $^{1}$H NMR (300 MHz, MeOD, major/minor diastereomer ratio 1/0.8). For major diastereomer *δ*: 7.75 (d, *J* = 7.4, 1H), 7.70 – 7.61 (m, 2H), 7.59 – 7.52 (m, 1H), 6.06 (s, 1H), 4.22 (dddd, *J* = 11.0, 8.5, 6.7, 3.0, 1H), 4.00 – 3.72 (m, 3H), 3.41 (dd, *J* = 14.2, 8.6, 1H), 2.24 – 1.86 (m, 3H), 1.83 – 1.63 (m, 1H). For minor diastereomer *δ*: 7.75 (d, *J* = 7.4, 1H), 7.70 – 7.62 (m, 2H), 7.59 – 7.52 (m, 1H), 5.97 (s, 1H), 4.22 (dddd, *J* = 11.0, 8.5, 6.7, 3.0, 1H), 4.03

– 3.69 (m, 3H), 3.57 (dd, $J$ = 14.3, 4.5, 1H), 2.17 – 1.87 (m, 3H), 1.79 – 1.63 (m, 1H). $^{13}$C NMR (75 MHz, MeOD) $\delta$: 169.8 (C=O), 169.4 (C=O), 146.4 (C), 146.2 (C), 133.6 (CH), 133.5 (CH), 132.6 (C), 132.6 (C), 130.7 (CH), 130.7 (CH), 124.6 (2xCH), 123.9 (CH), 123.8 (CH), 83.8 (major diastereomer, CH), 83.4 (CH), 79.3 (major diastereomer, CH), 78.5 (minor diastereomer, CH), 69.0 (major diastereomer, $CH_2$), 69.0 (minor diastereomer, $CH_2$), 44.5 (major diastereomer, $CH_2$), 43.7 (minor diastereomer, $CH_2$), 30.3 (major diastereomer, $CH_2$), 29.9 (minor diastereomer, $CH_2$), 26.6 (minor diastereomer, $CH_2$), 26.4 (major diastereomer, $CH_2$). HRMS (ESI+) [M+H$^+$; calculated for $C_{13}H_{16}NO_3$: 234.1125] found m/z 234.1122.

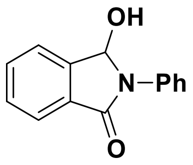


**3-Hydroxy-2-phenylisoindolin-1-one (9).**[7] Isolated yield: 92%. GC-MS (m/z, M$^{+\cdot}$ 225), major peaks found: 225 (71%), 209 (21%), 180 (31%), 154 (10%), 133 (53%), 105 (100%), 77 (70%), 51 (27%), 28 (4%). $^{1}$H NMR (300 MHz, MeOD) $\delta$: 7.80 (dd, $J$ = 7.5, 0.8 Hz, 1H), 7.75 – 7.65 (m, 4H), 7.63 – 7.54 (m, 1H), 7.47 – 7.41 (m, 2H), 7.30 – 7.22 (m, 1H), 6.43 (s, 1H). $^{13}$C NMR (75 MHz, MeOD) $\delta$: 168.5 (C=O), 145.7 (C), 138.3 (C), 134.1 (CH), 132.7 (C), 131.0 (CH), 130.0 (2xCH), 127.9 (CH), 124.9 (2xCH), 124.7 (CH), 124.2 (CH), 84.3 (CH). The NMR spectrum is consistent with the reported data.

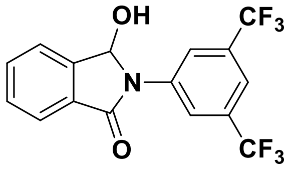


**2-(3,5-Bis(trifluoromethyl)phenyl)-3-hydroxyisoindolin-1-one (10).** Isolated yield: 83%. GC-MS (m/z, M$^{+\cdot}$ 361), major peaks found: 361 (41%), 342 (19%), 316 (19%), 294 (3%), 269 (3%), 246 (3%), 213 (8%), 194 (3%), 163 (7%), 133 (64%), 105 (100%), 77 (36%), 51 (12%). $^{1}$H NMR (300 MHz, MeOD) $\delta$: 8.58 (s, 2H), 7.88 (dd, $J$ = 7.6, 0.9, 1H), 7.81 – 7.74 (m, 3H), 7.68 – 7.61 (m, 1H), 6.63 (s, 1H). $^{13}$C NMR (75 MHz, MeOD) $\delta$: 168.6 (C=O), 145.5 (C), 141.1 (C), 134.9 (CH), 133.2 (q, $J^2_{C\text{-}F}$ = 33.3 Hz, 2xC), 132.0 (C), 131.3 (CH), 124.8 (CH), 124.8 (q, $J^1_{C\text{-}F}$= 272.2, 2xC), 124. (CH), 122.3 – 122.2 (m, 2xCH), 119.4 – 118.4 (m, CH), 84.0 (CH).$^{19}$F NMR (282 MHz, MeOD) $\delta$: - 64.4 (s, 6F). HRMS (ESI+) [M+H$^+$; calculated for $C_{16}H_{10}F_6NO_2$: 362.0610] found m/z 362.0613.

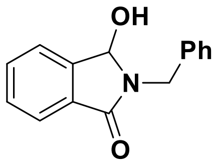


**2-Benzyl-3-hydroxyisoindolin-1-one (11).**[7] Isolated yield: 95%. GC-MS (m/z, M$^{+\cdot}$ 239), major peaks found: 239 (14%), 192 (2%), 160 (5%), 133 (14%), 106 (100%), 92 (30%), 77 (21%), 51 (10%), 28 (2%). $^{1}$H NMR (300 MHz, MeOD) $\delta$: 7.77 (d, $J$ = 7.4, 1H), 7.69 – 7.49 (m, 3H), 7.41 – 7.17 (m, 5H), 5.67 (s, 1H), 5.09 (d, $J$ = 15.1, 1H), 4.43 (d, $J$ = 15.1, 1H). $^{13}$C NMR (75 MHz, MeOD) $\delta$: 169.3 (C=O), 146.1 (C), 138.5 (C), 133.7 (CH), 132.6 (C), 130.8 (CH), 129.7 (2xCH), 129.1 (2xCH), 128.6 (CH), 124.7 (CH), 124.0 (CH), 82.1 (CH), 43.7 ($CH_2$).

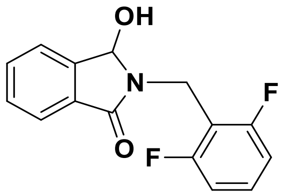

**2-(2,6-Difluorobenzyl)-3-hydroxyisoindolin-1-one (12).** Isolated yield: 79%. GC-MS (m/z, $M^{+\cdot}$ 275), major peaks found: 275 (21%), 258 (17%), 142 (100%), 127 (81%), 105 (35%), 77 (35%), 51 (17%). $^1$H NMR (300 MHz, $CD_3CN$) *δ*: 7.68 – 7.60 (m, 1H), 7.58 – 7.43 (m, 3H), 7.29 (tt, *J* = 8.3, 6.5, 1H), 6.92 (t, *J* = 8.2, 2H), 5.63 (d, *J* = 9.9, 1H), 4.93 (d, *J* = 14.8, 1H), 4.56 (d, *J* = 14.9, 1H), 4.19 (d, *J* = 9.9, 1H). $^{19}$F NMR (282 MHz, $CD_3CN$) *δ*: -115.51 (t, *J* = 6.9, 2F). HRMS (ESI+) [M+H$^+$; calculated for $C_{15}H_{12}F_2NO_2$: 276.0831] found m/z 276.0832.

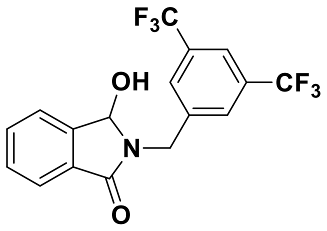

**2-(3,5-Bis(trifluoromethyl)benzyl)-3-hydroxyisoindolin-1-one (13).** Isolated yield: 86%. GC-MS (m/z, $M^{+\cdot}$ 375), major peaks found: 375 (12%), 358 (10%), 329 (4%), 242 (100%), 227 (55%), 207 (5%), 177 (16%), 160 (12%), 134 (71%), 105 (75%), 77 (37%), 51 (14%), 28 (3%). $^1$H NMR (300 MHz, MeOD) *δ*: 8.00 (s, 2H), 7.88 (s, 1H), 7.79 (d, *J* = 7.5, 1H), 7.71 – 7.62 (m, 2H), 7.61 – 7.52 (m, 1H), 5.84 (s, 1H), 4.91 (dd, *J* = 53.6, 15.7, 2H). $^{13}$C NMR (75 MHz, MeOD) *δ*: 169.7 (C=O), 146.2 (C), 142.5 (C), 133.9 (CH), 132.9 (q, $J^2_{C\text{-}F}$ = 33.3, 2xC), 132.2 (C), 130.9 (CH), 129.8 – 129.8 (m, 2xCH), 124.8 (q, $J^1_{C\text{-}F}$ = 271.9, 2xC), 124.8 (CH), 122.4 – 122.1 (m, CH), 124.1 (CH), 83.4 (CH), 44.0 ($CH_2$). $^{19}$F NMR (282 MHz, MeOD) *δ*: -64.33 (s, 6F). HRMS (ESI+) [M+H$^+$; calculated for $C_{17}H_{12}F_6NO_2$: 376.0767] found m/z 376.0769.

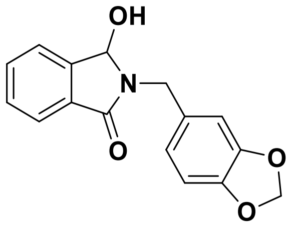

**2-(Benzo[*d*][1,3]dioxol-5-ylmethyl)-3-hydroxyisoindolin-1-one (14).**[8] Isolated yield: 71%. GC-MS (m/z, $M^{+\cdot}$ 283), major peaks found: 283 (100%), 267 (49%), 207 (77%), 151 (42%), 135 (100%), 118 (32%), 91 (32%), 77 (27%), 51 (36%), 28 (55%). $^1$H NMR (300 MHz, MeOD) *δ*: 7.77 (d, *J* = 7.5, 1H), 7.67 – 7.62 (m, 1H), 7.61 (dd, *J* = 6.8, 0.2, 1H), 7.56 (td, *J* = 7.3, 1.4, 1H), 6.85 – 6.83 (m, 2H), 6.77 (d, *J* = 8.3, 1H), 5.92 (dd, *J* = 3.6, 1.1, 2H), 5.68 (s, 1H), 4.99 (d, *J* = 14.9, 1H), 4.32 (d, *J* = 14.9, 1H). $^{13}$C NMR (75 MHz, MeOD) *δ*: 169.2 (C=O), 149.5 (C), 148.6 (C), 146.1 (C), 133.7 (CH), 132.6 (C), 132.3 (C), 130.8 (CH), 124.7 (CH), 123.9 (CH), 122.7 (CH), 109.5 (CH), 109.2 (CH), 102.4 ($CH_2$), 82.0 (CH), 43.5 ($CH_2$). The NMR spectrum is consistent with the reported data.

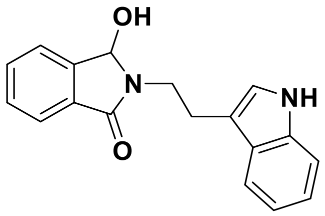

**2-(2-(1*H*-Indol-3-yl)ethyl)-3-hydroxyisoindolin-1-one (15).** Isolated yield: 68%. GC-MS (m/z, $M^{+\cdot}$ 292), major peaks found: 292 (3%), 270 (100%), 241 (17%), 214 (8%), 188 (3%), 164 (2%), 143 (2%), 121 (5%), 94 (4%), 77 (3%), 63 (2%), 39 (2%). $^{1}$H NMR (401 MHz, MeOD) *δ*: 7.73 (d, *J* = 7.6, 1H), 7.67 – 7.43 (m, 4H), 7.32 (d, *J* = 8.1, 1H), 7.13 – 7.04 (m, 2H), 7.01 – 6.84 (m, 1H), 5.66 (s, 1H), 4.08 – 3.95 (m, 1H), 3.72 (ddd, *J* = 13.7, 8.9, 6.7, 1H), 3.26 – 3.01 (m, 2H). $^{13}$C NMR (101 MHz, MeOD) *δ*: 169.4 (C=O), 146.2 (C), 138.1 (C), 133.37 (CH), 132.9 (C), 130.6 (CH), 128.7 (C), 124.5 (CH), 123.7 (CH), 123.4 (CH), 122.4 (CH), 119.7 (CH), 119.3 (CH), 113.1 (C), 112.2 (CH), 83.2 (CH), 41.5 ($CH_2$), 25.2 ($CH_2$). HRMS (ESI+) [M+H$^+$; calculated for $C_{18}H_{17}N_2O_2$: 293.1285] found m/z 293.1282.

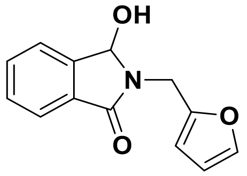

**2-(Furan-2-ylmethyl)-3-hydroxyisoindolin-1-one (16).**[9] Isolated yield: 89%. GC-MS (m/z, $M^{+\cdot}$ 229), major peaks found: 229 (100%), 182 (2%), 154 (8%), 133 (16%), 104 (40%), 96 (100%), 81 (21%), 77 (21%), 53 (15%), 27 (4%). $^{1}$H NMR (300 MHz, MeOD) *δ*: 7.73 (dt, *J* = 7.4, 0.9, 1H), 7.67 – 7.57 (m, 2H), 7.55 – 7.50 (m, 1H), 7.45 – 7.34 (m, 1H), 6.36 – 6.35 (m, 2H), 5.75 (s, 1H), 5.00 (d, *J* = 15.7, 1H), 4.45 (d, *J* = 15.7, 1H). $^{13}$C NMR (75 MHz, MeOD) *δ*: 168.9 (C=O), 151.8 (C), 146.1 (C), 143.7 (CH), 133.7 (CH), 132.4 (C), 130.8 (CH), 124.7 (CH), 124.0 (CH), 111.4 (CH), 109.2 (CH), 82.4 (CH), 36.5 ($CH_2$). The NMR spectrum is consistent with the reported data.

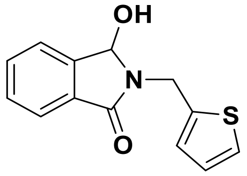

**3-Hydroxy-2-(thiophen-2-ylmethyl)isoindolin-1-one (17).**[10] Isolated yield: 84%. GC-MS (m/z, $M^{+\cdot}$ 245), major peaks found: 245 (16%), 228 (3%), 198 (5%), 171 (4%), 134 (15%), 112 (100%), 109 (37%), 97 (37%), 77 (22%), 45 (11%), 28 (8%). $^{1}$H NMR (300 MHz, MeOD) *δ*: 7.76 (dt, *J* = 7.4, 0.8, 1H), 7.68 – 7.59 (m, 2H), 7.59 – 7.51 (m, 1H), 7.31 (dd, *J* = 5.1, 1.2, 1H), 7.10 (dd, *J* = 3.4, 0.6, 1H), 6.97 (dd, *J* = 5.1, 3.5, 1H), 5.75 (s, 1H), 5.18 (d, *J* = 15.5, 1H), 4.66 (d, *J* = 15.5, 1H). $^{13}$C NMR (75 MHz, MeOD) *δ*: 168.8 (C=O), 146.1 (C), 140.7 (C), 133.8 (CH), 132.4 (C), 130.8 (CH), 127.9 (CH), 127.9 (CH), 126.7 (CH), 124.7 (CH), 124.0 (CH), 81.9 (CH), 38.1 ($CH_2$). The NMR spectrum is consistent with the reported data.

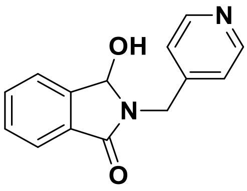

**3-Hydroxy-2-(pyridin-4-ylmethyl)isoindolin-1-one (18).** Isolated yield: 87%. GC-MS (m/z, $M^{+\cdot}$ 240), major peaks found: 240 (42%), 222 (11%), 193 (4%), 160 (4%), 133 (4%), 107 (100%), 77 (3%), 51 (2%). $^{1}$H NMR (300 MHz, MeOD) *δ*: 8.48 (dd, *J* = 4.6, 1.5, 2H), 7.83 – 7.77 (m, 1H), 7.71 – 7.62 (m, 2H), 7.61 – 7.53 (m, 1H), 7.40 (dd, *J* = 4.6, 1.6, 2H), 5.82 (s, 1H), 4.95 (d, *J* = 16.3, 1H), 4.67 (d, *J* = 16.3, 1H). $^{13}$C NMR (75 MHz, MeOD) *δ*: 169.6 (C=O), 150.3 (2xCH), 149.5 (C), 146.3 (C), 133.9 (C), 132.2 (CH), 130.9 (CH), 124.8 (CH), 124.4 (2xCH), 124.1 (CH), 83.2 (CH), 43.3 ($CH_2$). HRMS (ESI+) [M+H$^+$; calculated for $C_{14}H_{13}N_2O_2$: 241.0972] found m/z 241.0975.

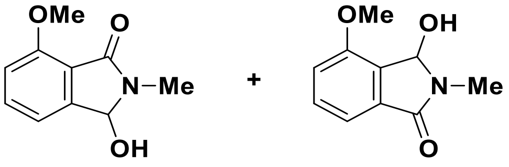

**(74%)** **(26%)**

**3-Hydroxy-7-methoxy-2-methylisoindolin-1-one and 3-hydroxy-4-methoxy-2-methylisoindolin-1-one (19).** Isolated yield: 73%. Both compounds were obtained as a mixture of regioisomers, and the molar relationship between them was determined by $^{1}$H NMR.

*For major regioisomer:* GC-MS, only one peak was detected by GC-MS (m/z, $M^{+\cdot}$ 192), major peaks found: 192 (71%), 176 (61%), 160 (16%), 133 (19%), 121 (13%), 105 (10%), 76 (14%), 51 (6%), 28 (6%), 11 (2%). $^{1}$H NMR (300 MHz, MeOD) *δ*: 7.54 – 7.44 (m, 1H), 7.30 (dd, *J* = 7.5, 0.6, 1H), 7.21 (d, *J* = 8.1, 1H), 5.81 (s, 1H), 3.94 (s, 3H), 3.07 (s, 3H). $^{13}$C NMR (75 MHz, MeOD) *δ*: 169.2 (C=O), 157.0 (C), 135.3 (C), 132.7 (C), 132.4 (CH), 116.0 (CH), 115.7 (CH), 82.9 (CH), 56.2 ($CH_3$), 26.2 ($CH_3$). HRMS (ESI+) [M+H$^+$; calculated for $C_{10}H_{12}NO_3$: 194.0812] found m/z 194.0809.

*For minor regioisomer:* GC-MS, only one peak was detected by GC-MS (m/z, $M^{+\cdot}$ 192), major peaks found: 192 (71%), 176 (61%), 160 (16%), 133 (19%), 121 (13%), 105 (10%), 76 (14%), 51 (6%), 28 (6%), 11 (2%). $^{1}$H NMR (300 MHz, MeOD) *δ*: 7.58 (dd, *J* = 8.3, 7.5, 1H), 7.17 (d, *J* = 7.4, 1H), 7.09 (d, *J* = 8.4, 1H), 5.65 (s, 1H), 3.92 (s, 3H), 3.04 (s, 3H). $^{13}$C NMR (75 MHz, $CDCl_3$) *δ*: 168.3 (C=O), 158.1 (C), 148.6 (C), 134.7 (CH), 119.3 (C), 116.5 (CH), 113.3 (CH), 83.6 (CH), 56.2 ($CH_3$), 26.1 ($CH_3$). HRMS (ESI+) [M+H$^+$; calculated for $C_{10}H_{12}NO_3$: 194.0812] found m/z 194.0809.

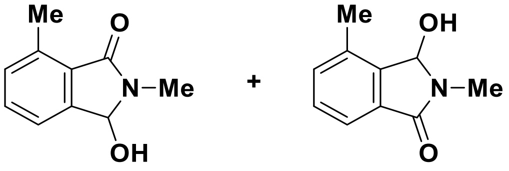

**(65%)** **(35%)**

**3-Hydroxy-2,7-dimethylisoindolin-1-one and 3-hydroxy-2,4-dimethylisoindolin-1-one (20).** Isolated yield: 87%. Both compounds were obtained as a mixture of regioisomers, and the molar relationship between them was determined by $^{1}$H NMR.

*For major regioisomer:* GC-MS, only one peak was detected by GC-MS (m/z, $M^{+\cdot}$ 177), major peaks found: 177 (73%), 160 (100%), 119 (39%), 91 (38%), 77 (8%), 63 (27%), 32 (15%). $^{1}$H NMR (300 MHz, MeOD) *δ:* 7.56 – 7.39 (m, 3H), 5.78 (s, 1H), 3.09 (s, 3H), 2.49 (s, 3H).$^{13}$C NMR (75 MHz, MeOD) *δ*: 169.6 (C=O), 143.6 (C), 135.9 (C), 134.9 (CH),

132.8 (C), 130.5 (CH), 120.8 (CH), 83.8 (CH), 25.9 ($CH_3$), 17.1 ($CH_3$). HRMS (ESI+) [$M+H^+$; calculated for $C_{10}H_{12}NO_2$: 178.0863] found m/z 178.0863.

*For minor regioisomer:* GC-MS, only one peak was detected by GC-MS (m/z, $M^{+\cdot}$ 177), major peaks found: 177 (73%), 160 (100%), 119 (39%), 91 (38%), 77 (8%), 63 (27%), 32 (15%). $^{1}H$ NMR (300 MHz, MeOD) $\delta$: 7.57 – 7.35 (m, 2H), 7.27 (d, $J$ = 7.4, 1H), 5.7 (s, 1H), 3.1 (s, 3H), 2.6 (s, 3H). $^{13}C$ NMR (75 MHz, MeOD) $\delta$: 170.0 (C=O), 146.5 (C), 137.9 (C), 132.8 (CH), 132.3 (CH), 129.7 (C), 121.6 (CH), 83.3 (CH), 25.8 ($CH_3$), 17.1 ($CH_3$). HRMS (ESI+) [$M+H^+$; calculated for $C_{10}H_{12}NO_2$: 178.0863] found m/z 178.0863.

## 7. NMR SPECTRA OF THE ISOLATED ORGANIC COMPOUNDS

### $^1$H NMR (Compound **2**)

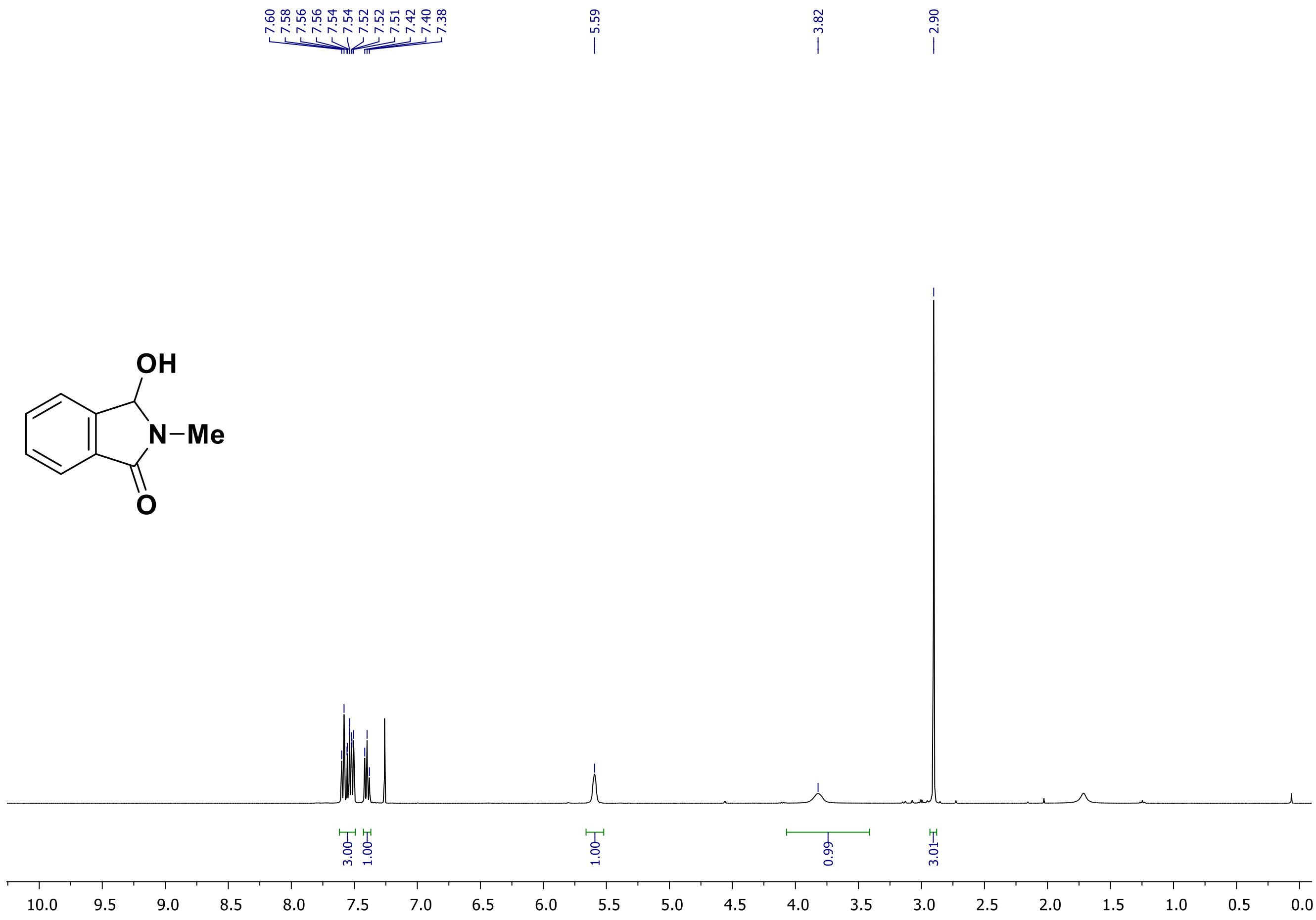


### $^{13}$C NMR (Compound **2**)

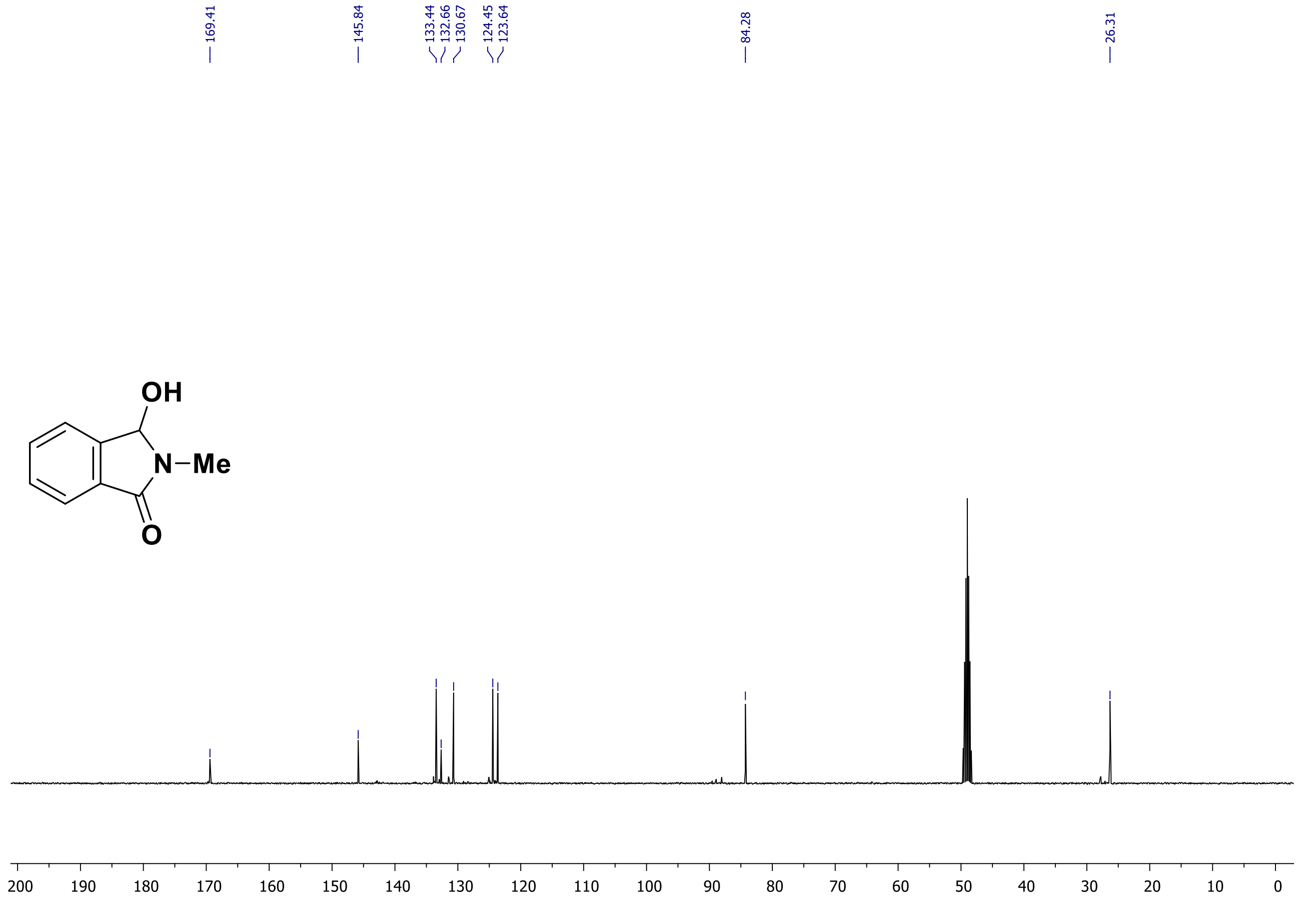

**$^{1}$H NMR** (Compound **6**)

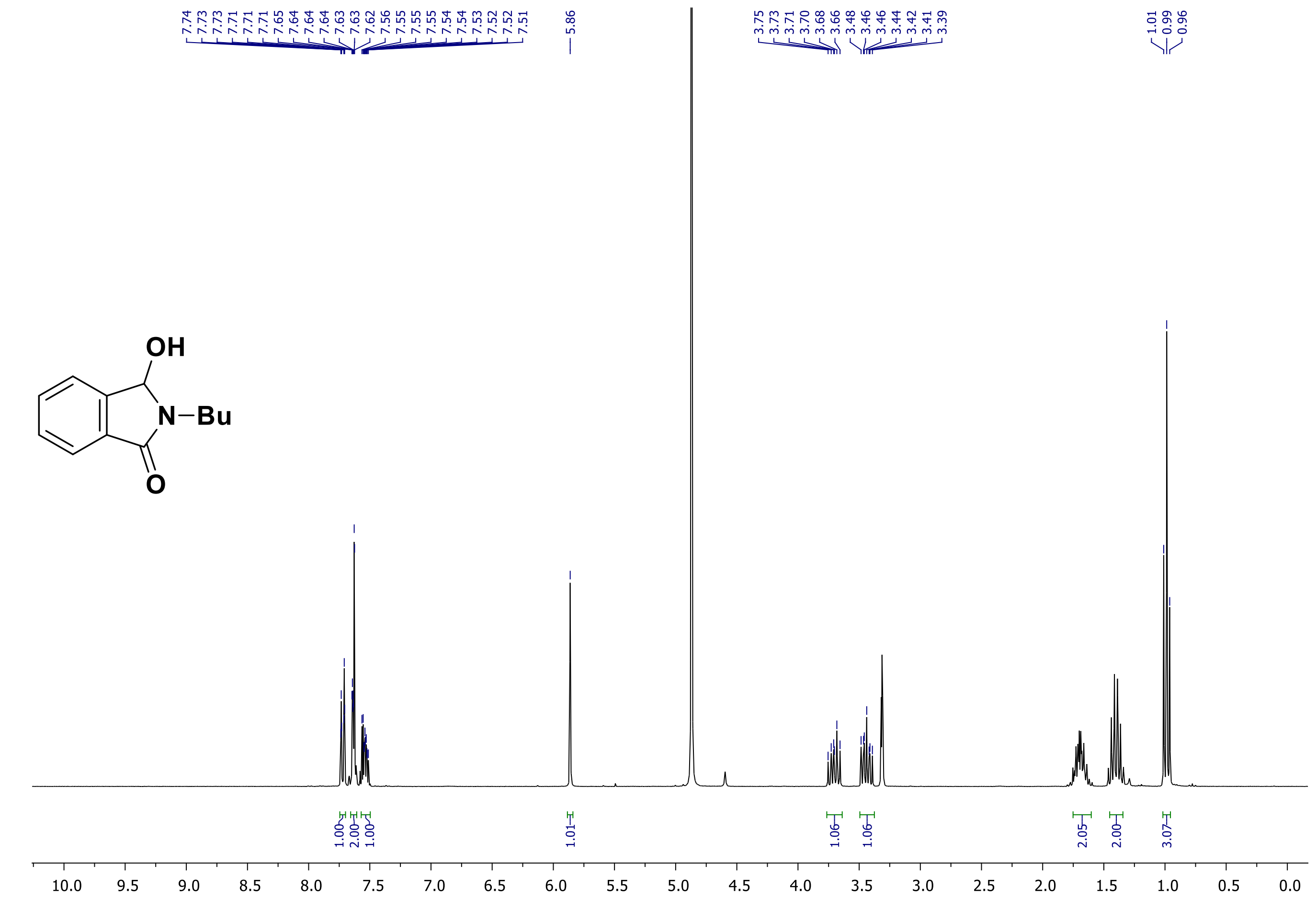


**$^{13}$C NMR** (Compound **6**)

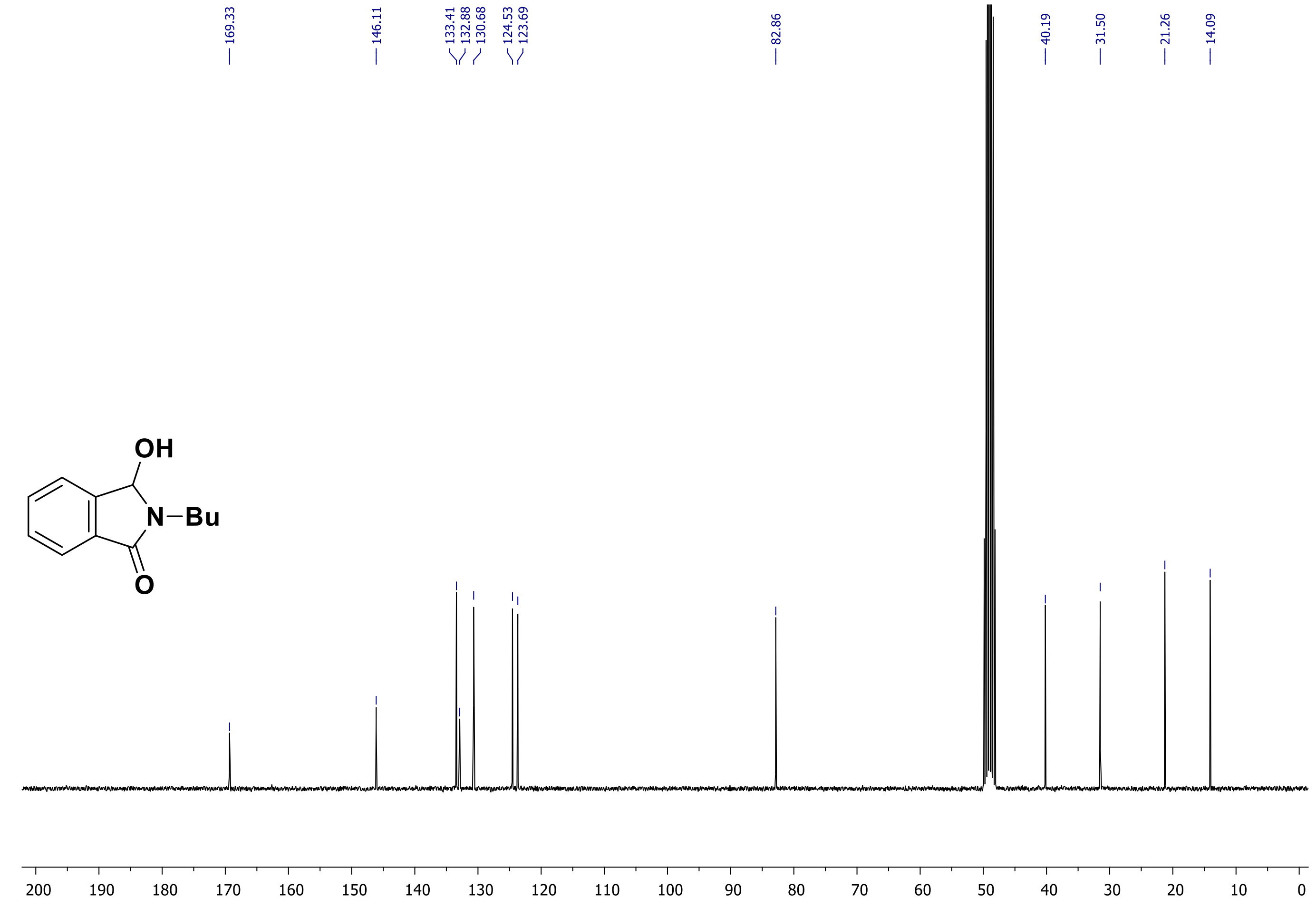

$^{1}$H NMR (Compound **7**)

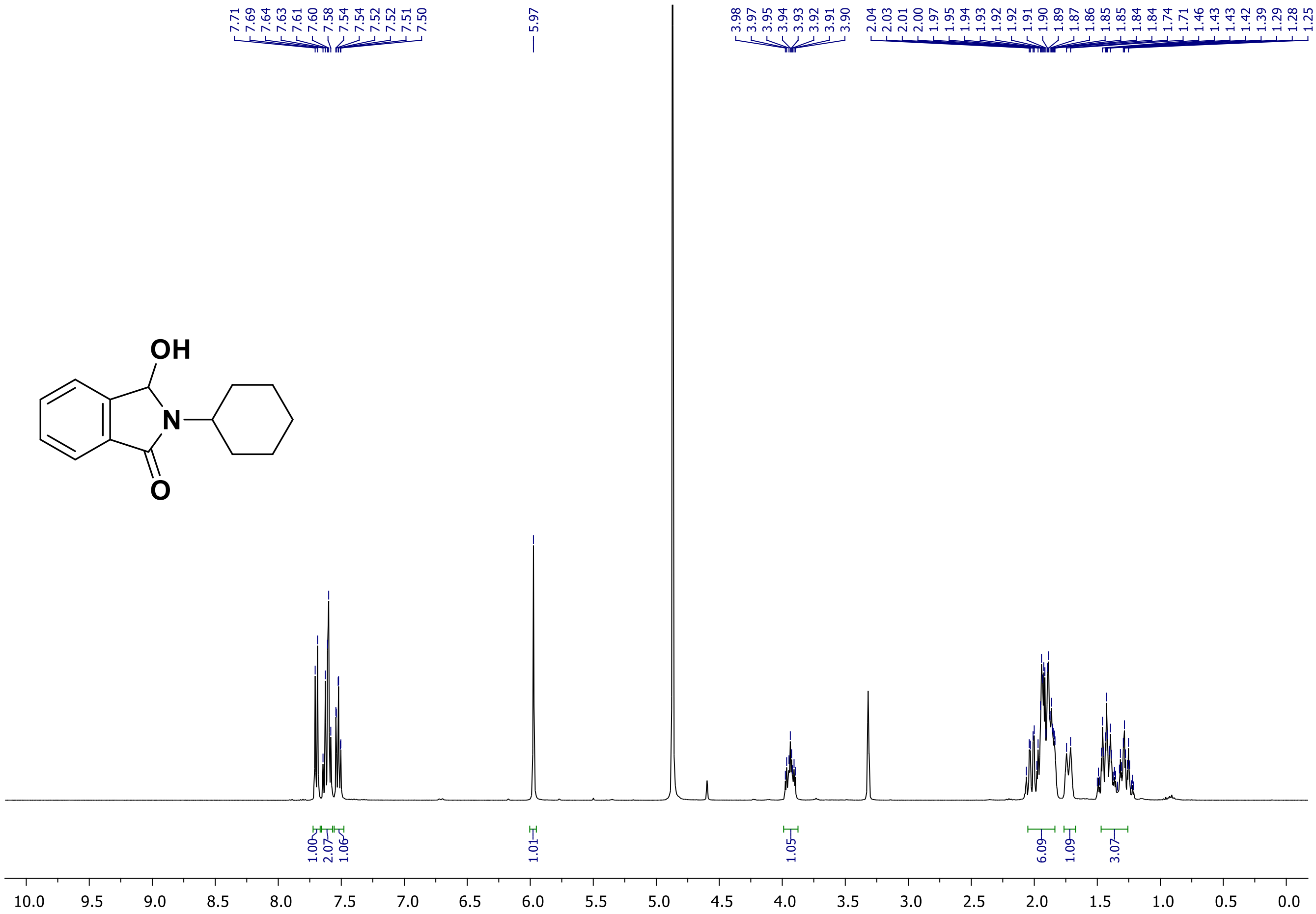


$^{13}$C NMR (Compound **7**)

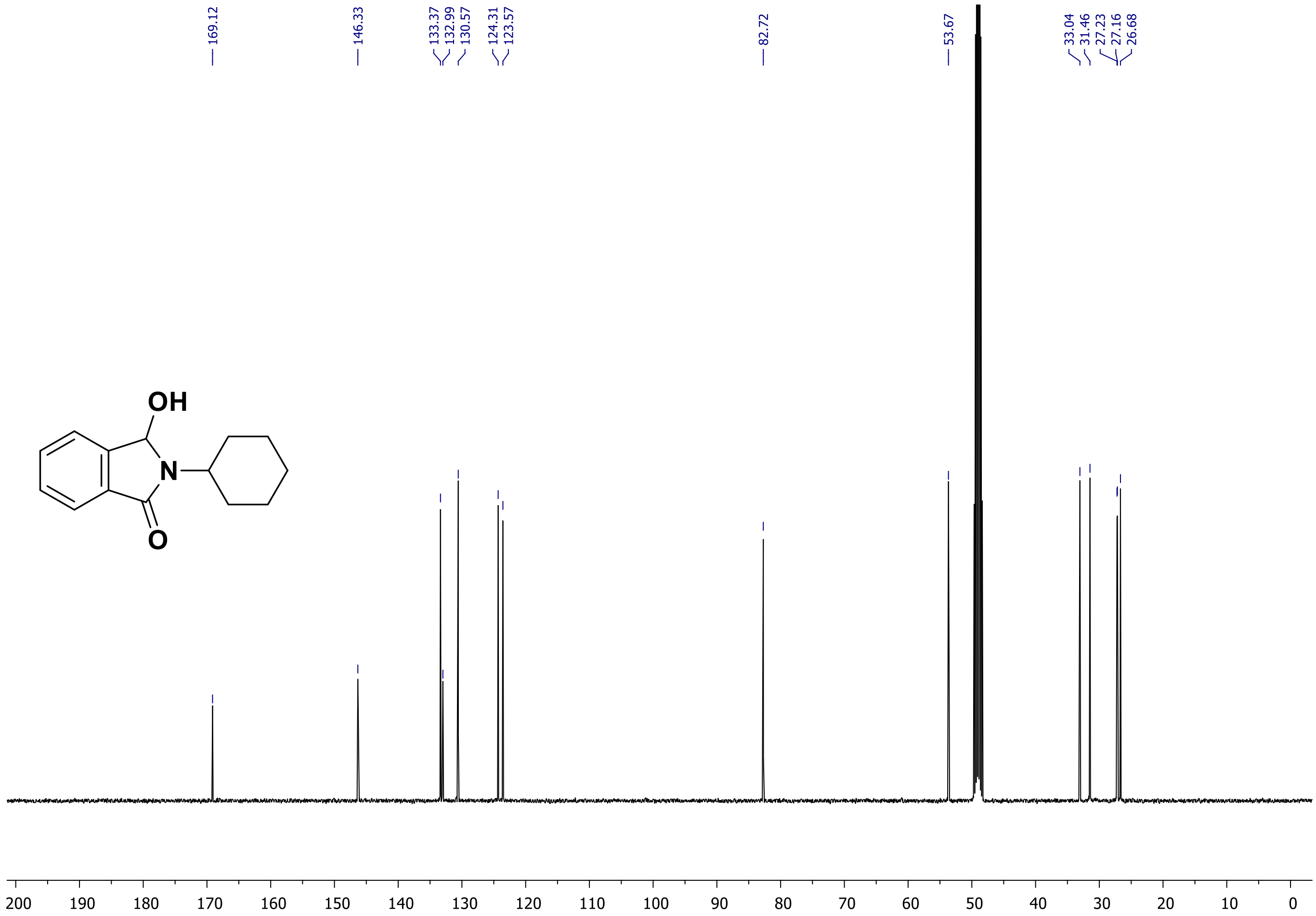

**$^{1}$H NMR** (Compound **8**)

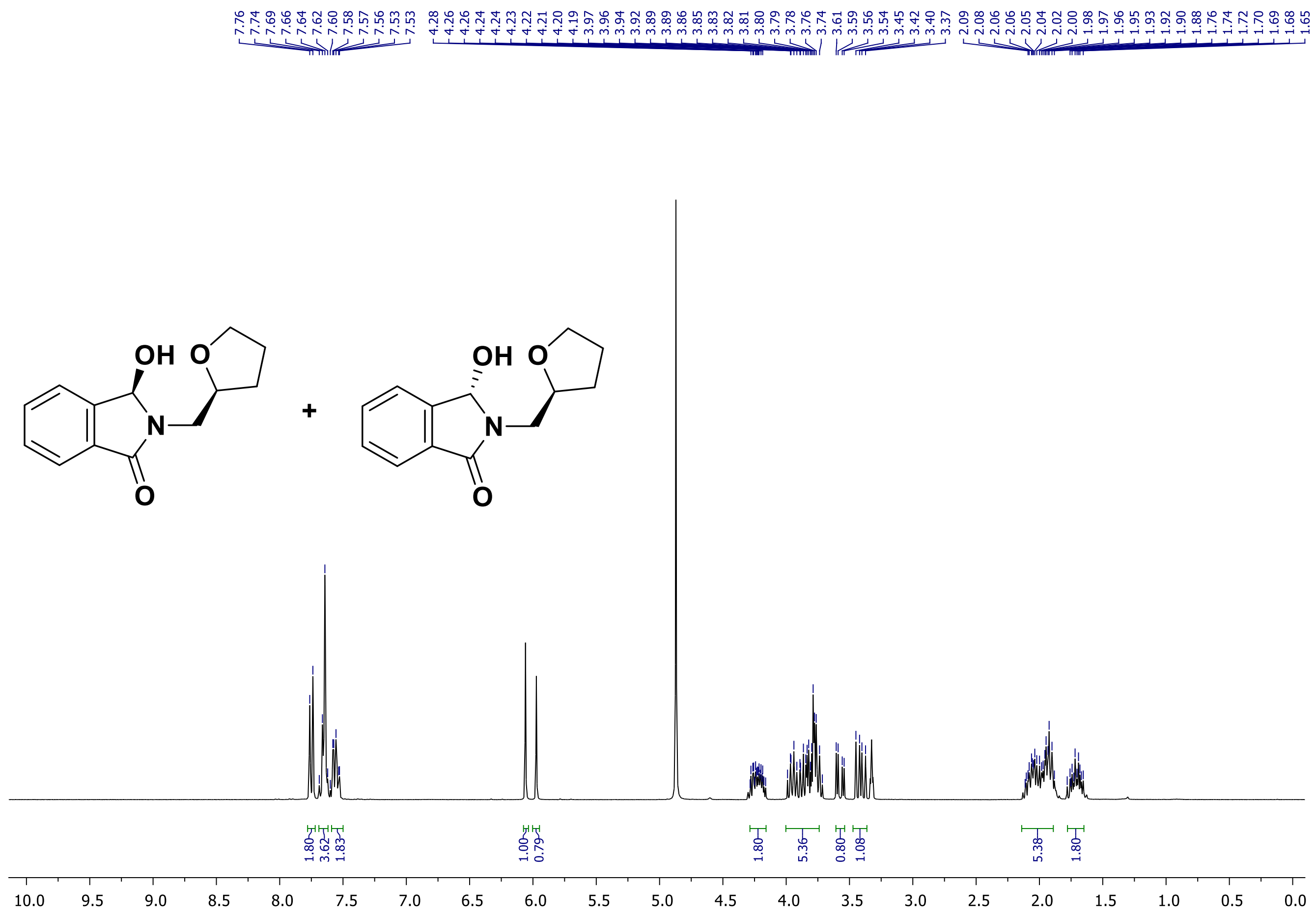


**$^{13}$C NMR** (Compound **8**)

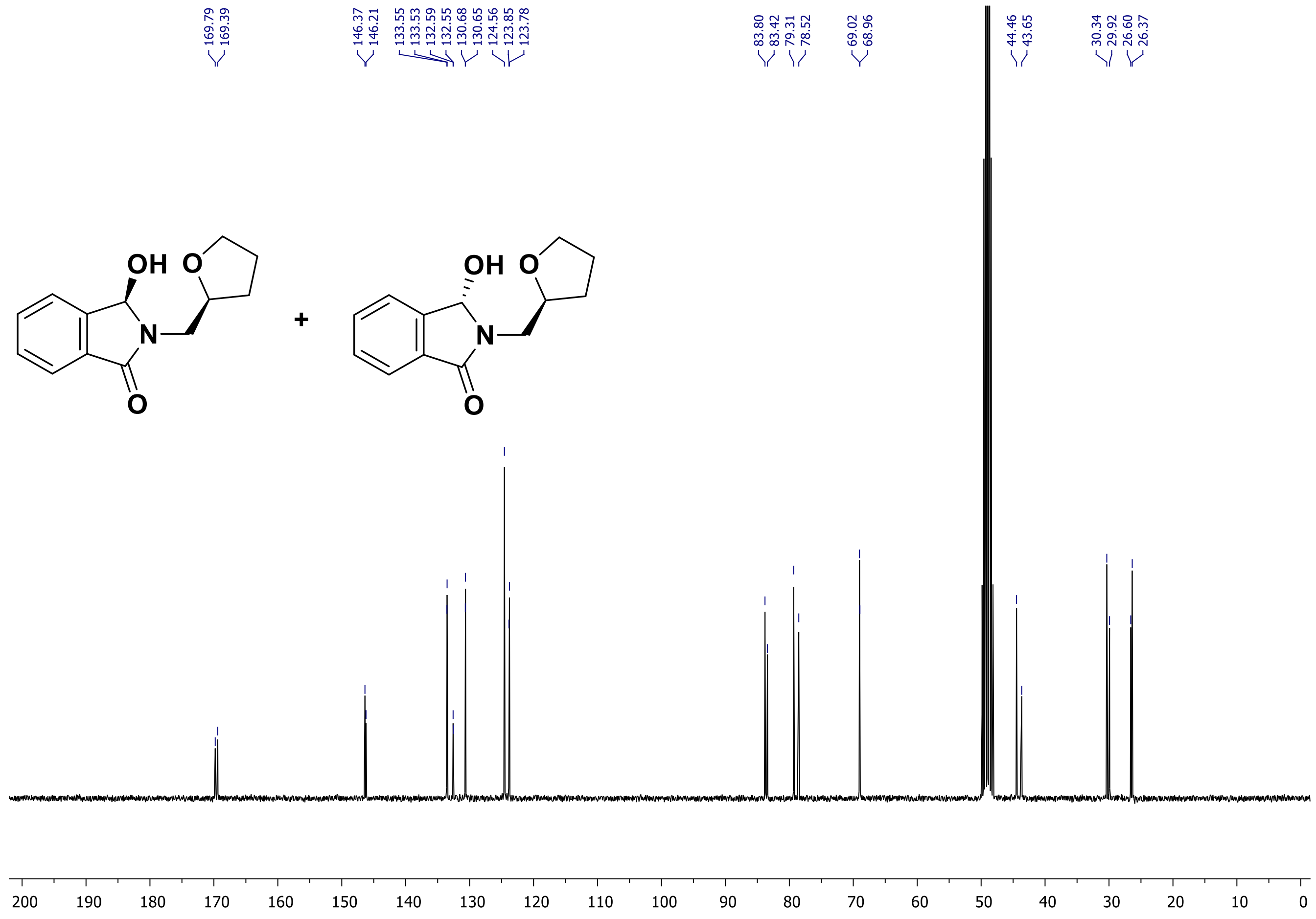

**$^{1}$H NMR** (Compound **9**)

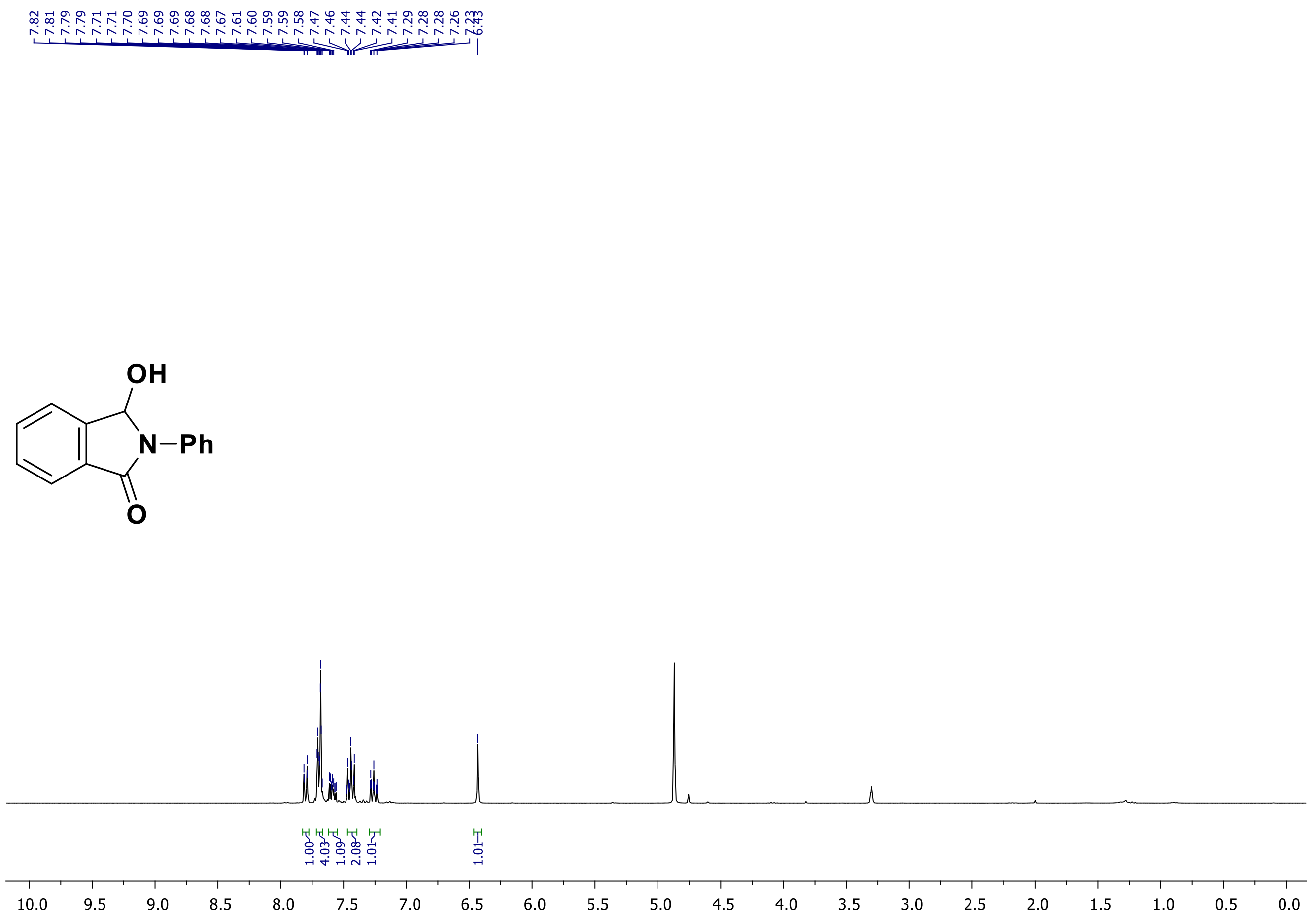


**$^{13}$C NMR** (Compound **9**)

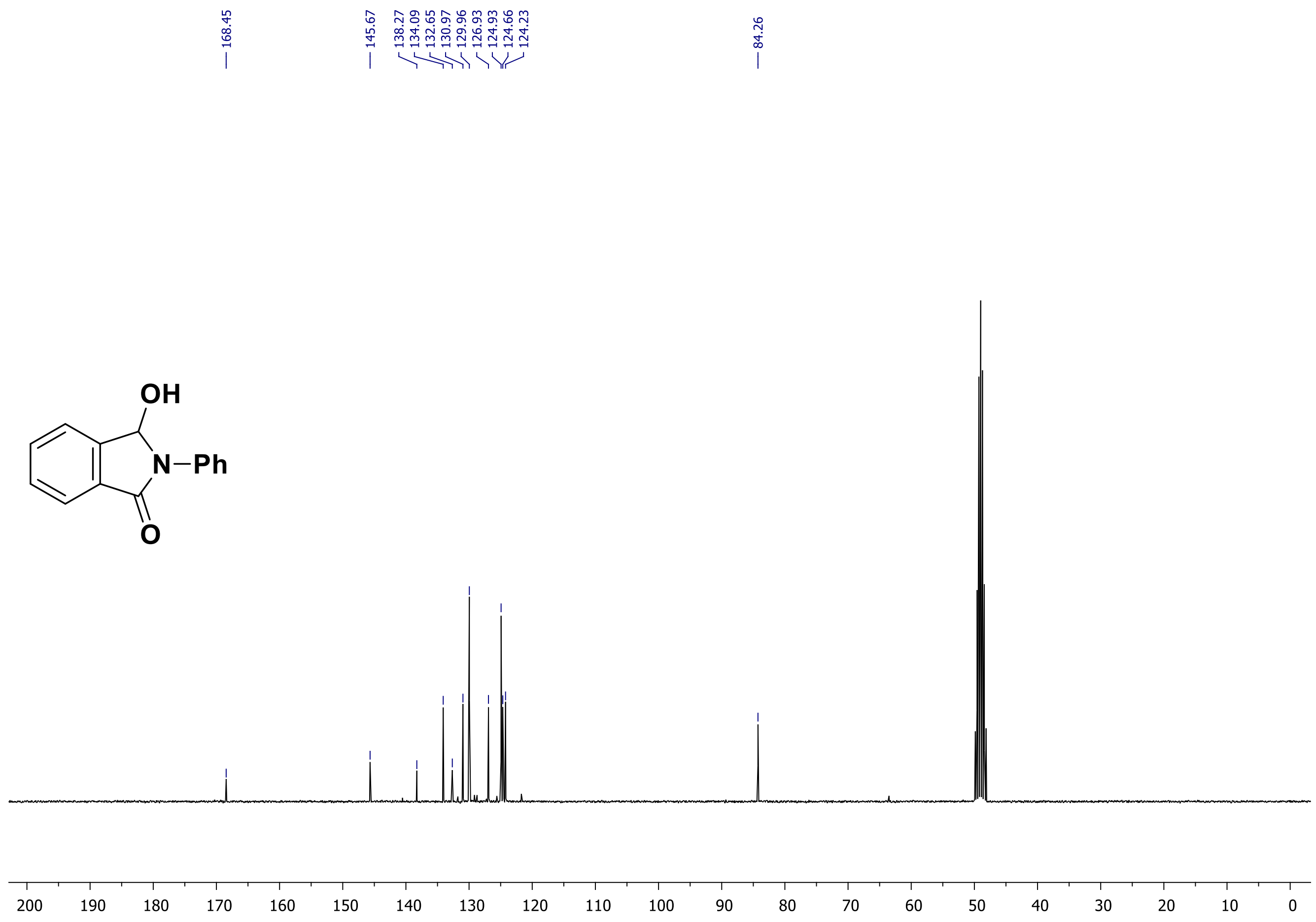

**$^{1}$H NMR** (Compound **10**)

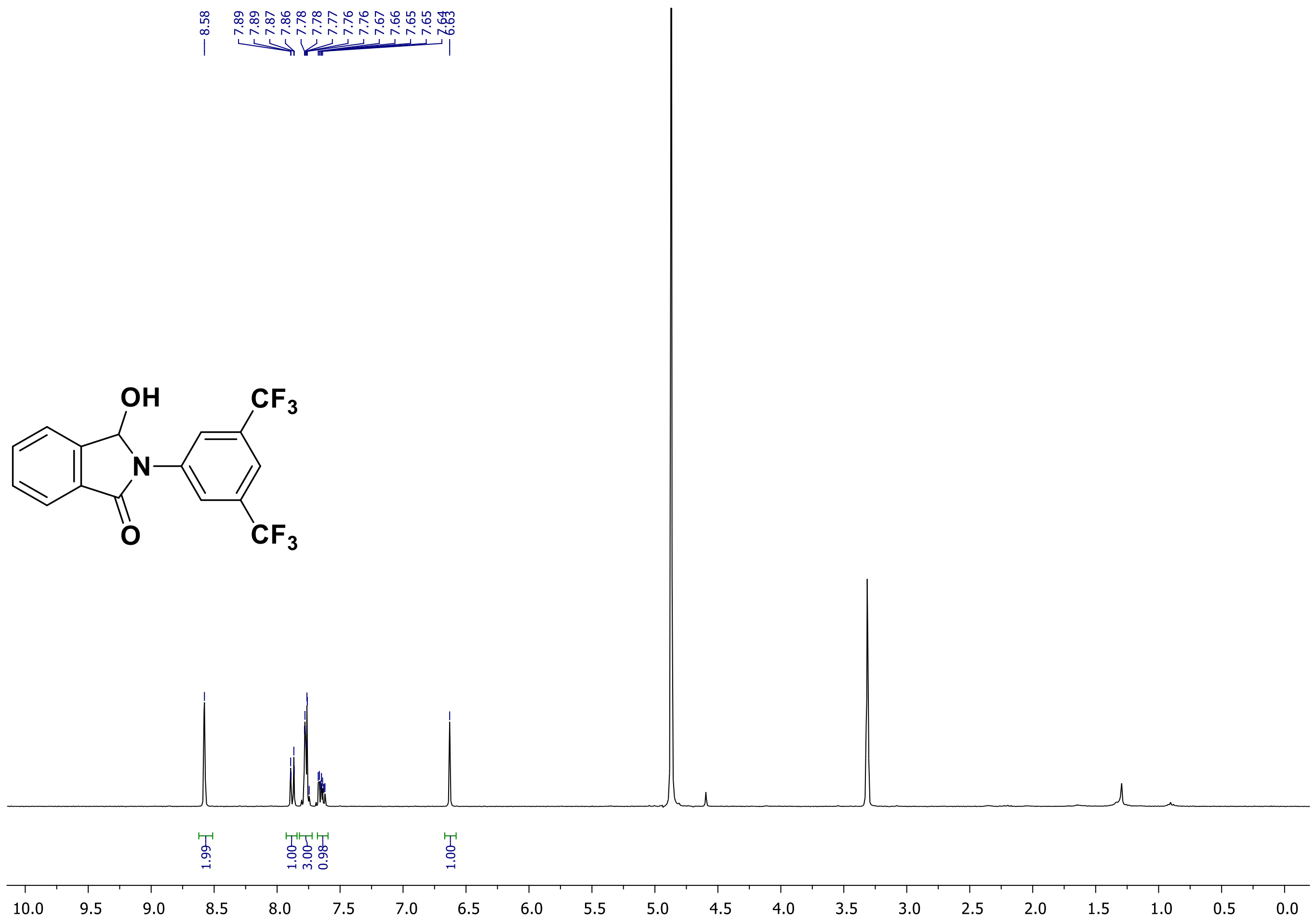


**$^{13}$C NMR** (Compound **10**)

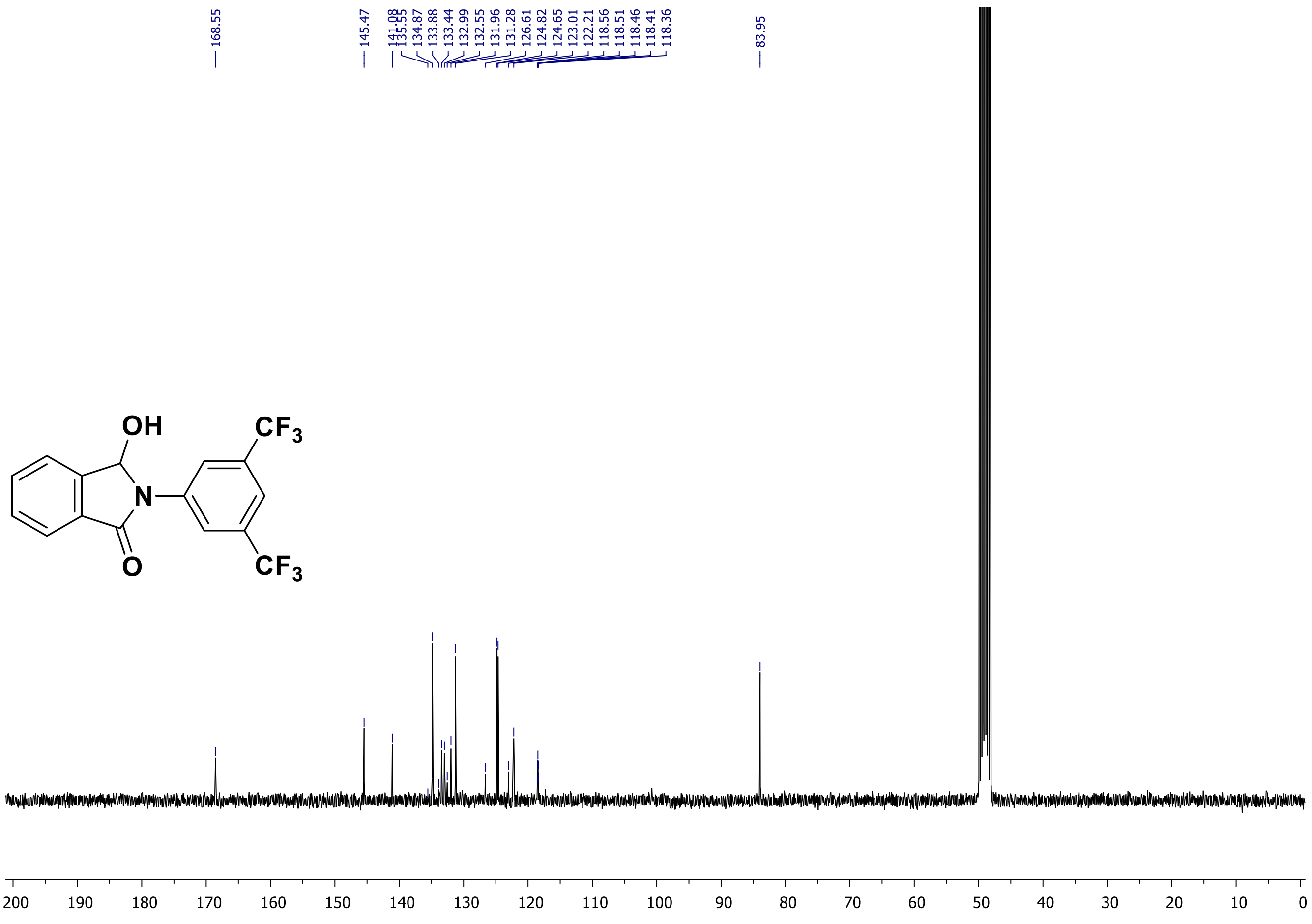

**$^{19}$F NMR** (Compound **10**)

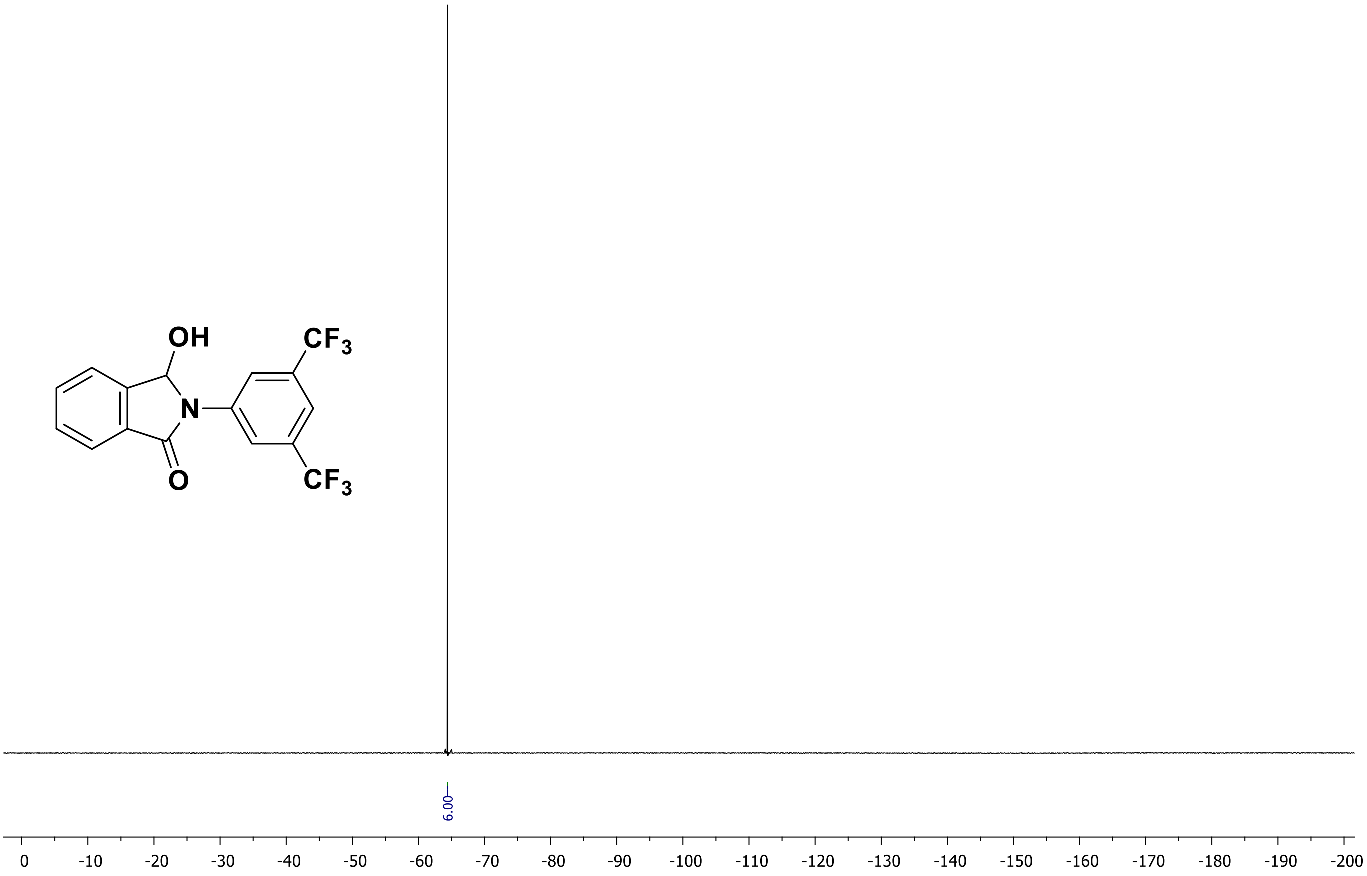

$^{1}$**H NMR** (Compound **11**)

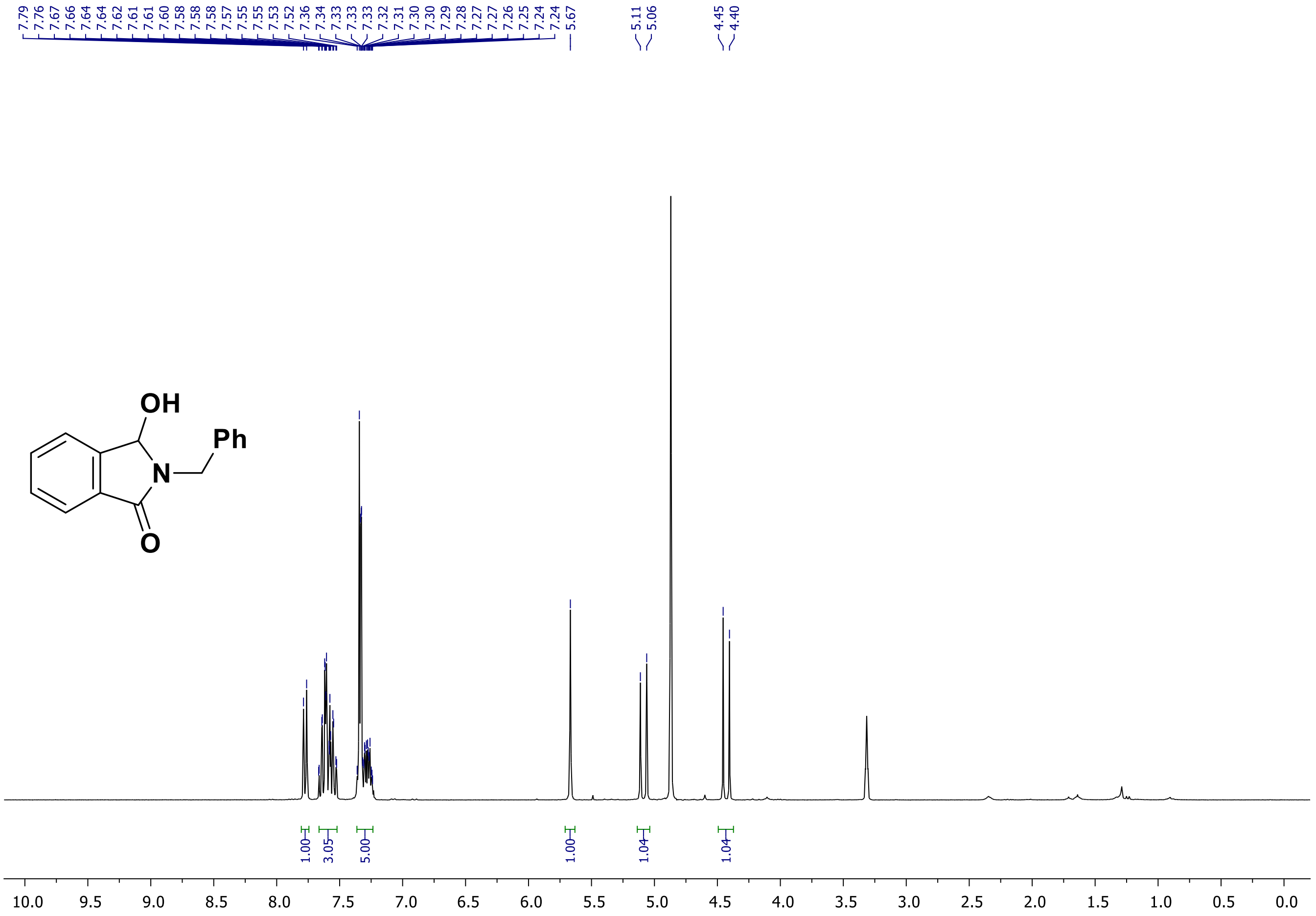


$^{13}$**C NMR** (Compound **11**)

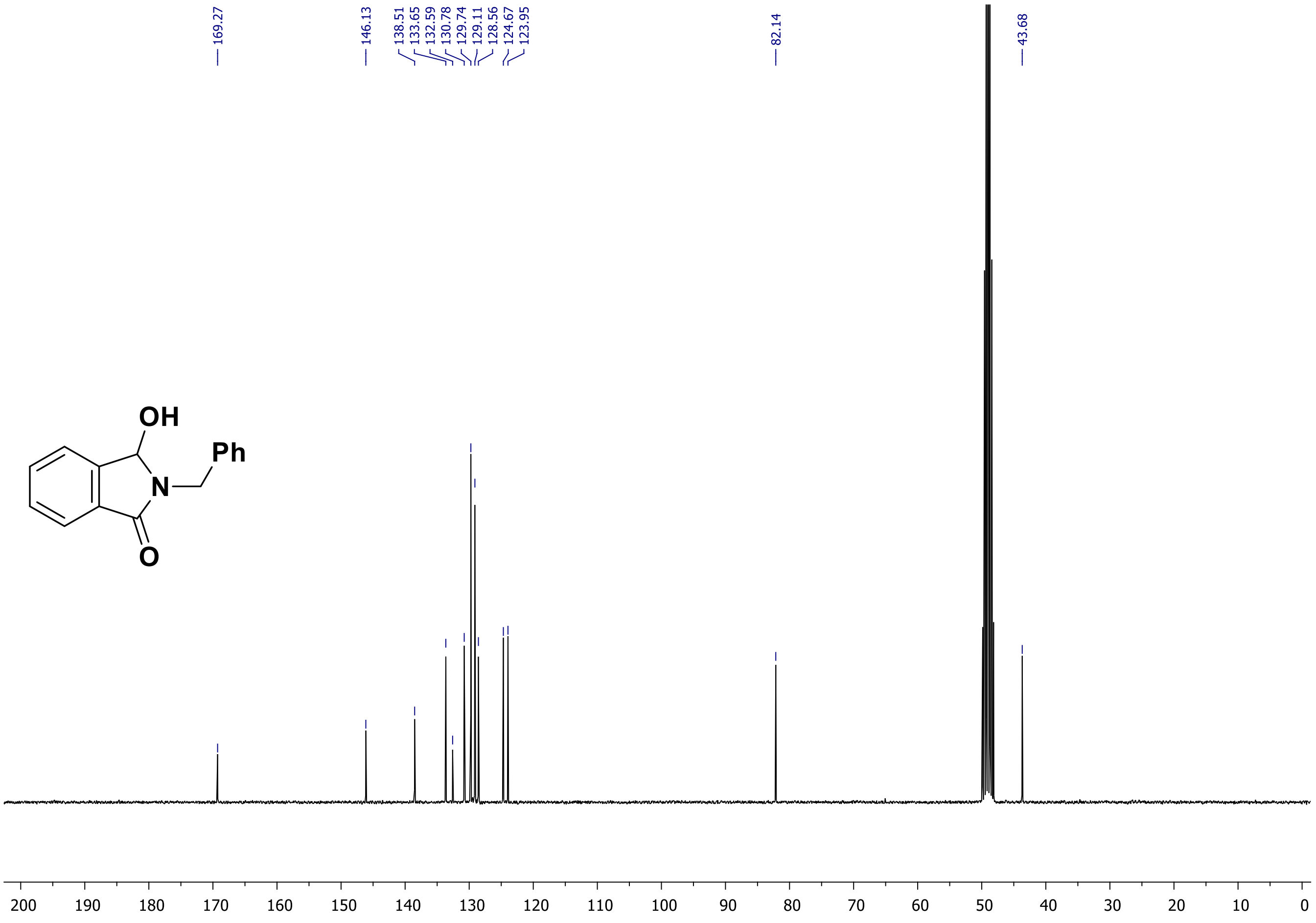

**$^{1}$H NMR** (Compound **12**)

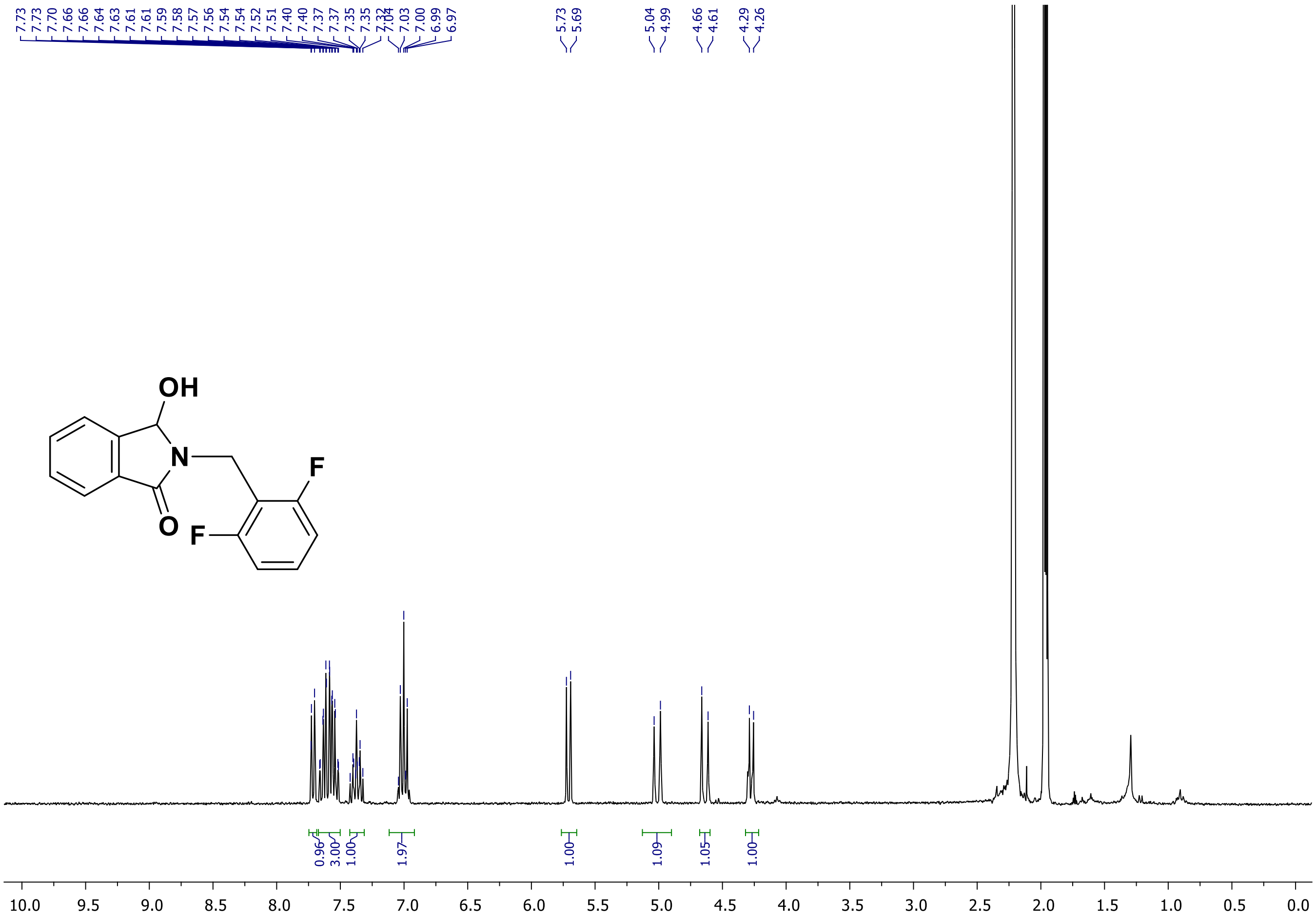


**$^{19}$F NMR** (Compound **12**)

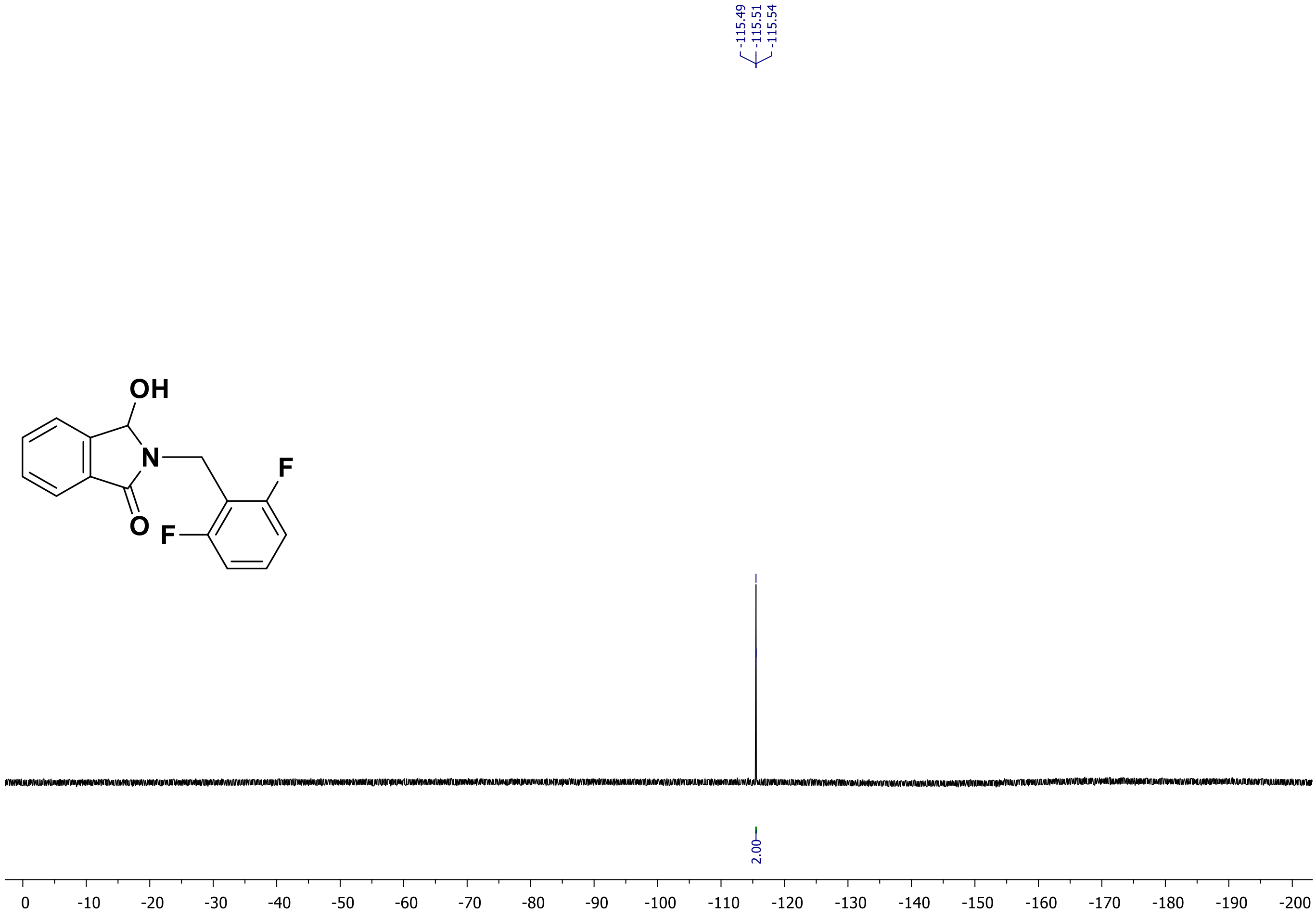

**$^{1}$H NMR** (Compound **13**)

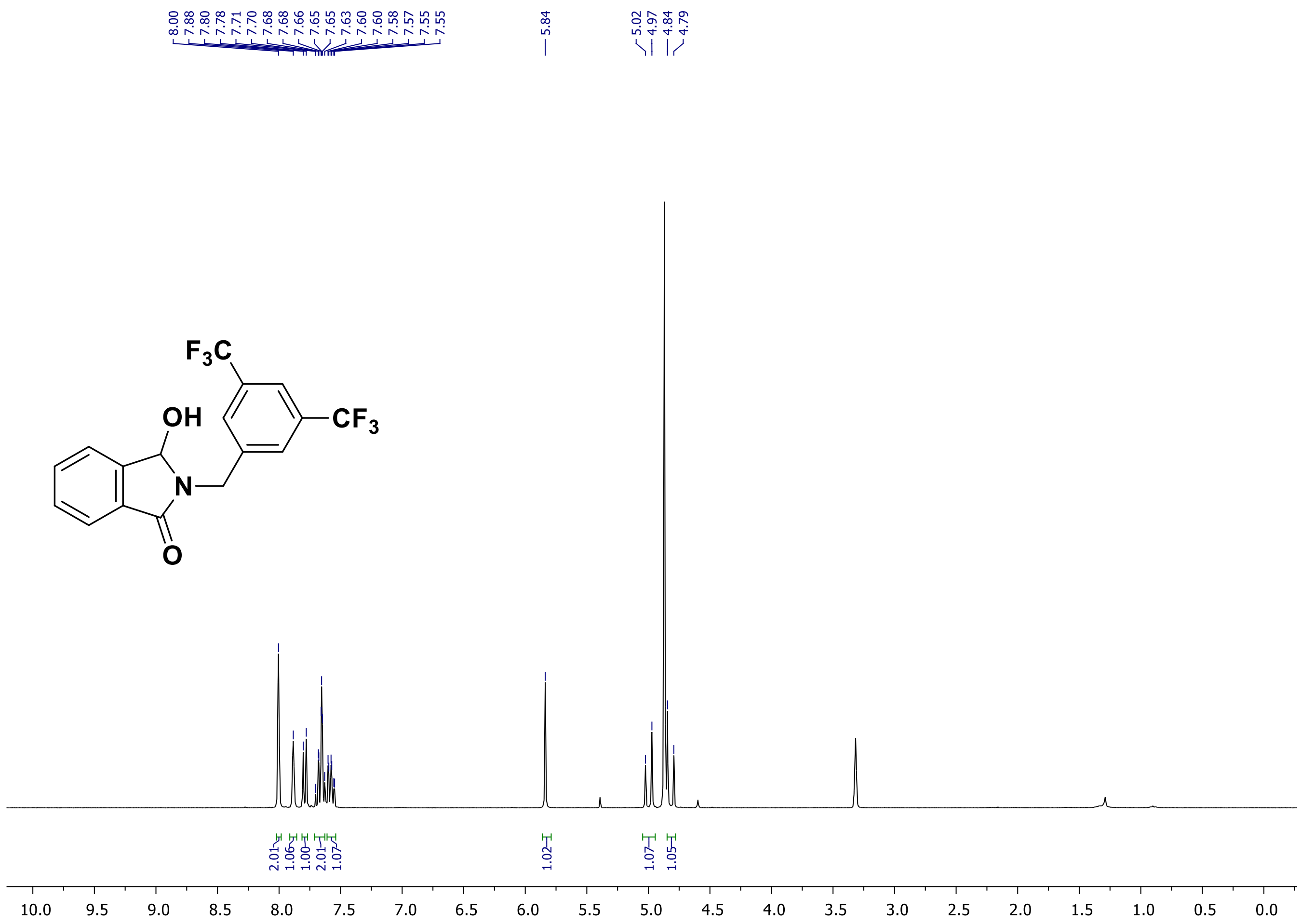


**$^{13}$C NMR** (Compound **13**)

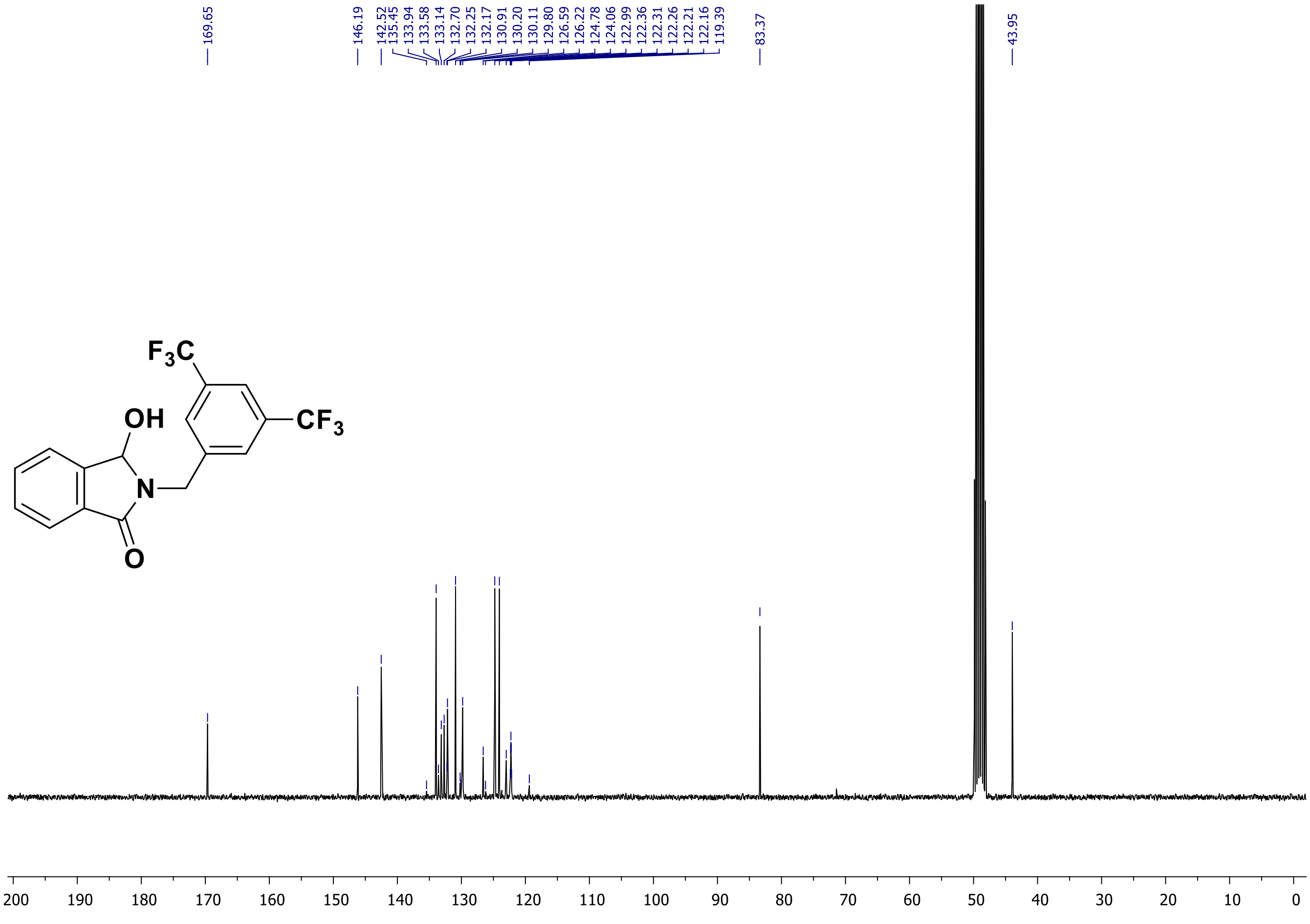

**$^{19}$F NMR** (Compound **13**)

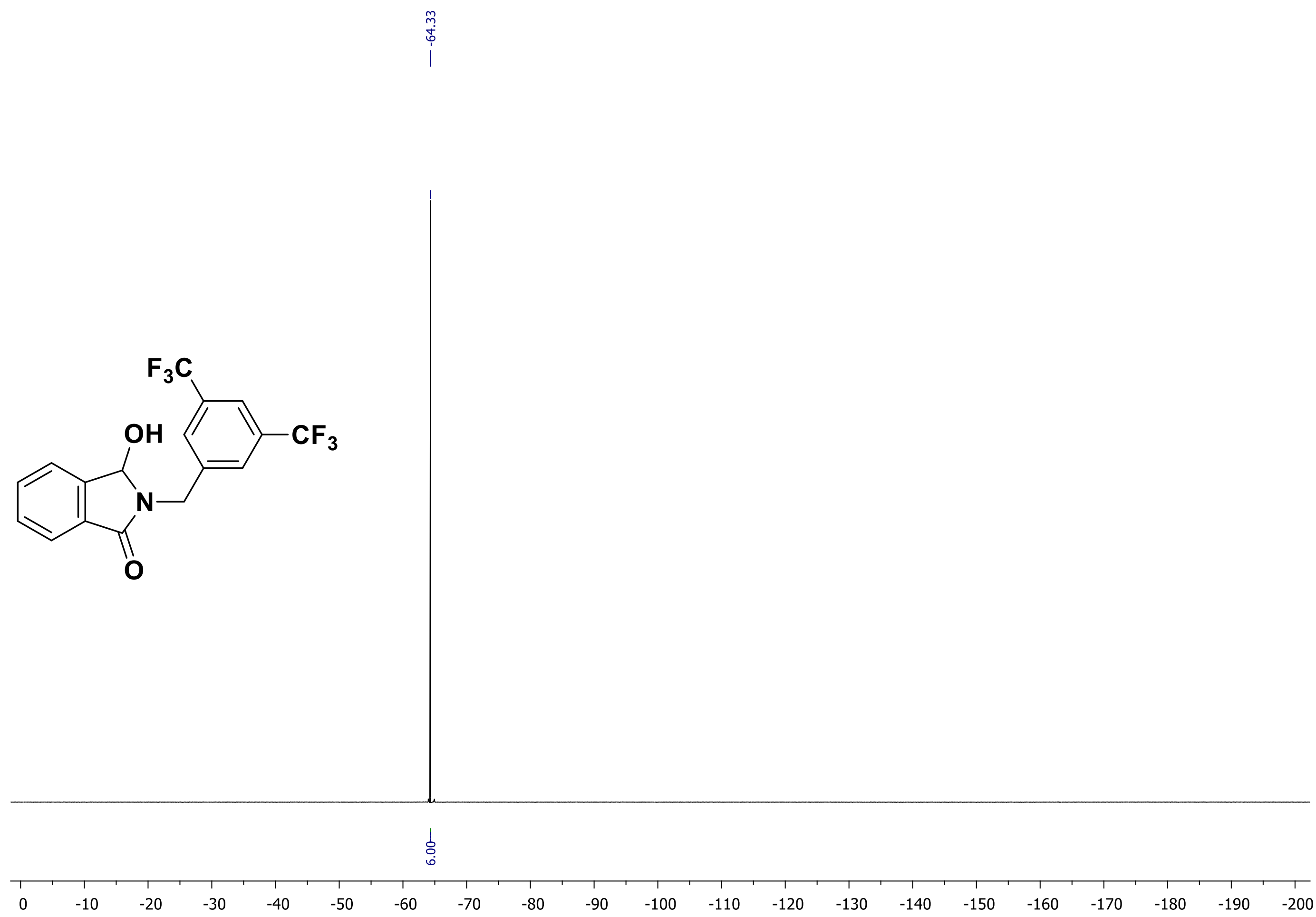

**$^{1}$H NMR** (Compound **14**)

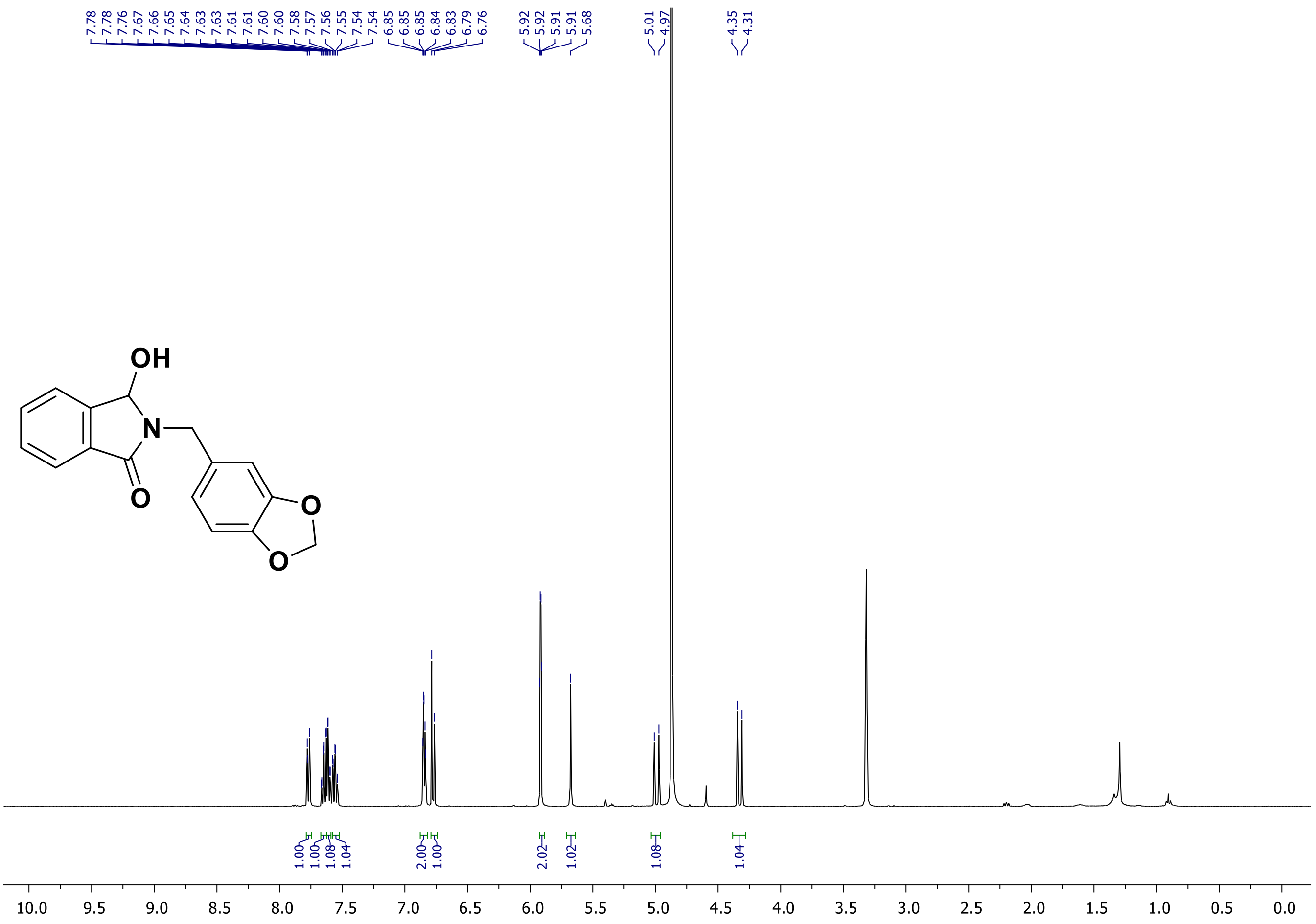

**$^{13}$C NMR** (Compound **14**)

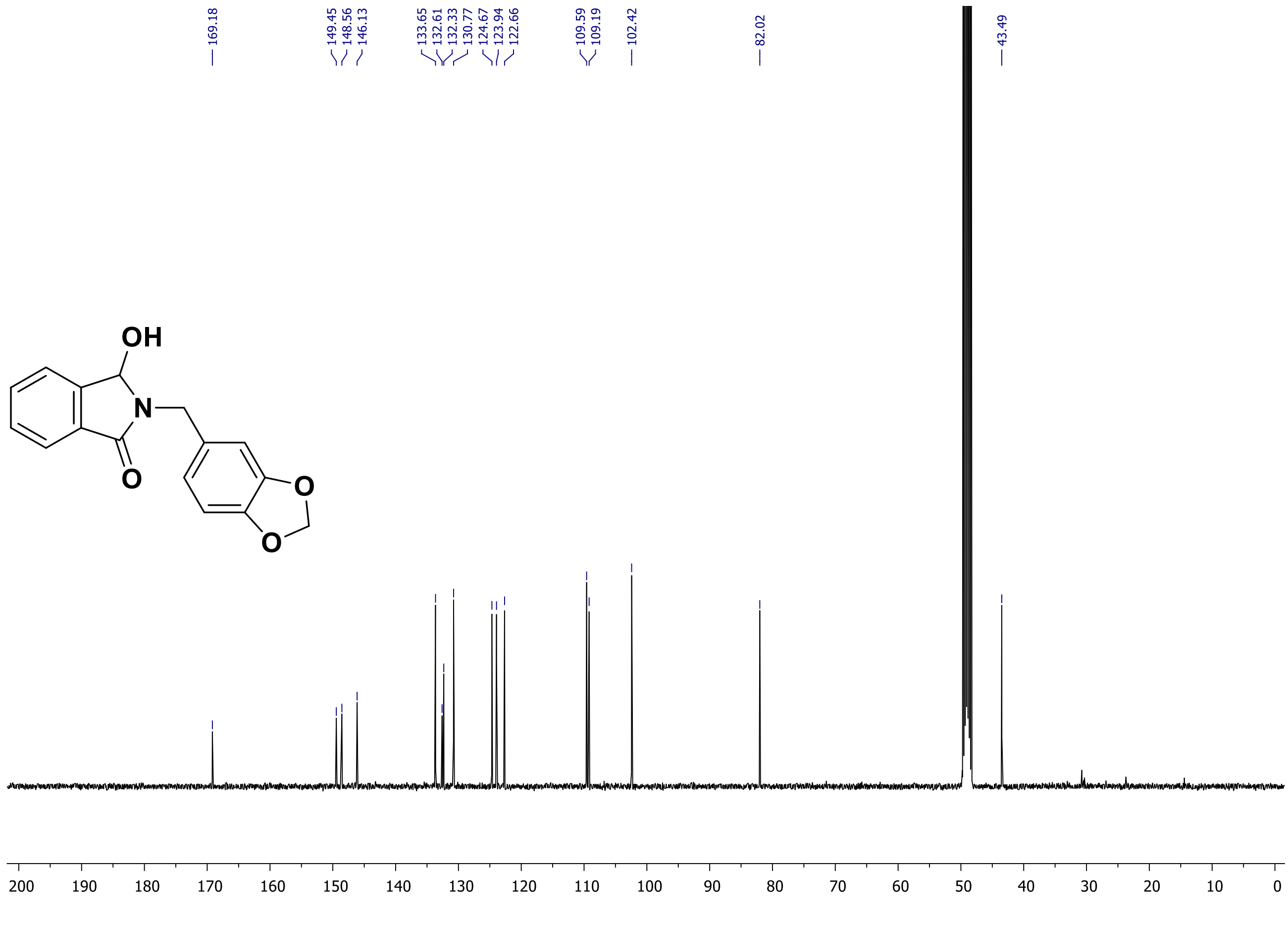

$^{1}$H NMR (Compound **15**)

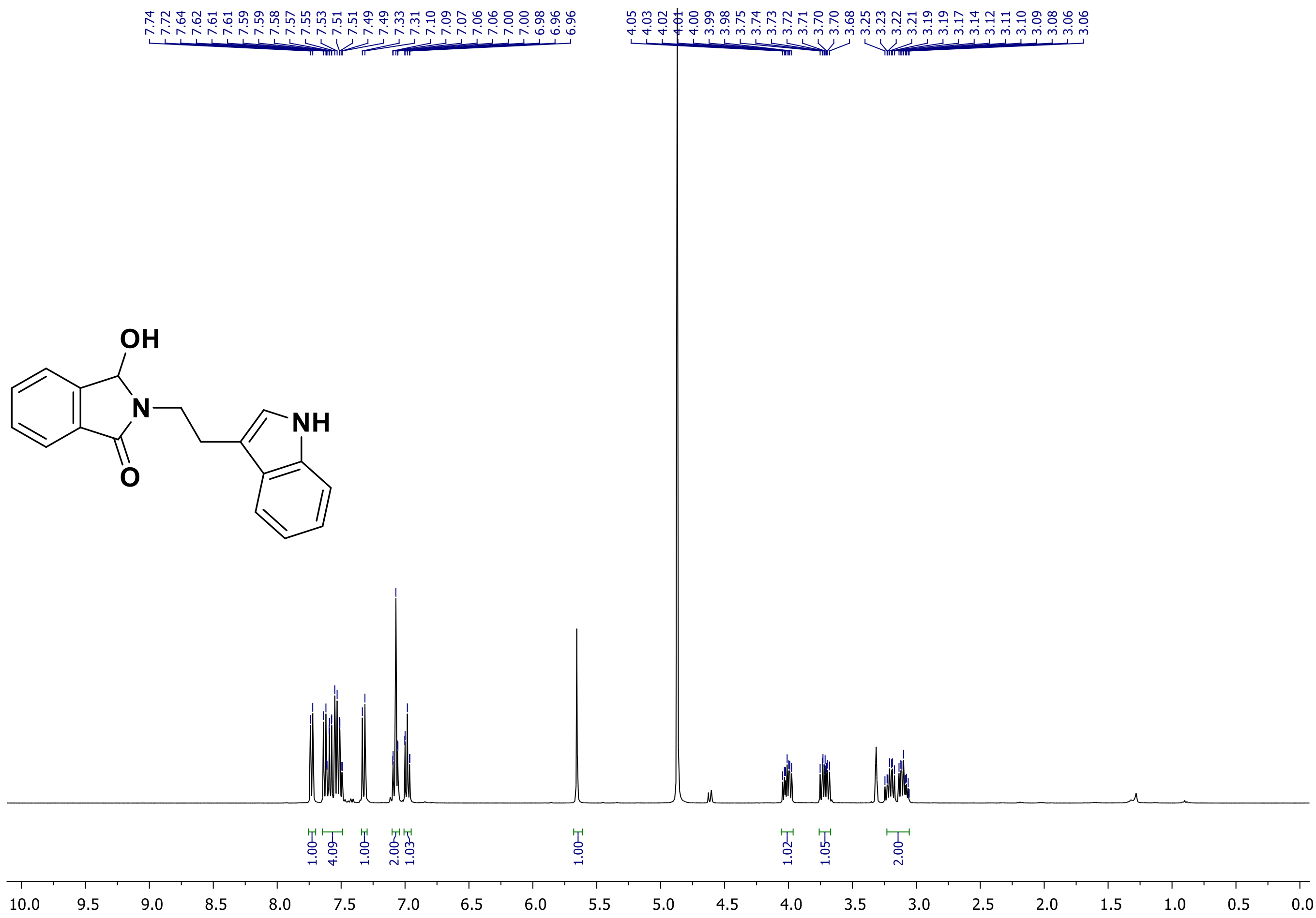

$^{13}$C NMR (Compound **15**)

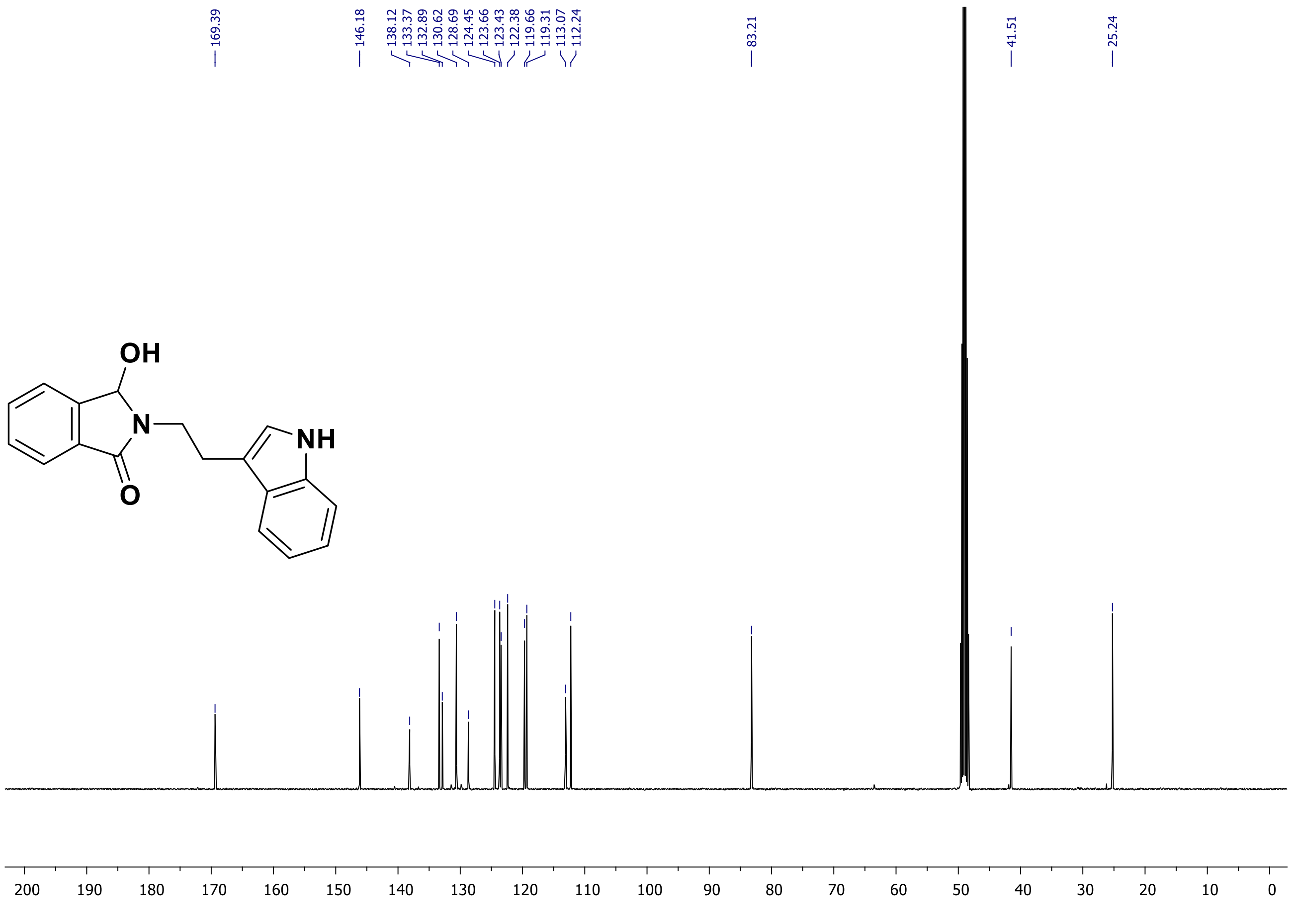

**$^1$H NMR** (Compound **16**)

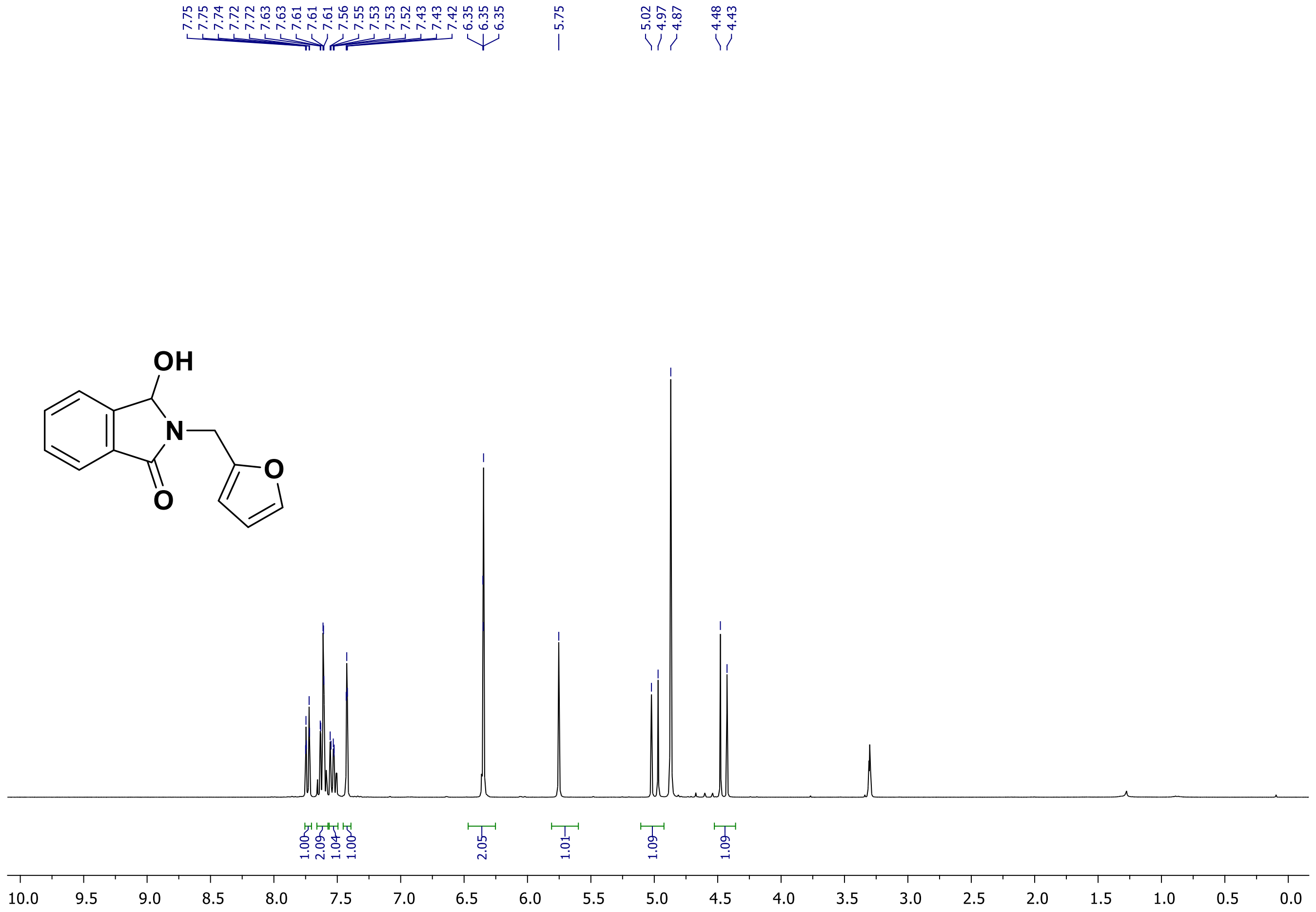


**$^{13}$C NMR** (Compound **16**)

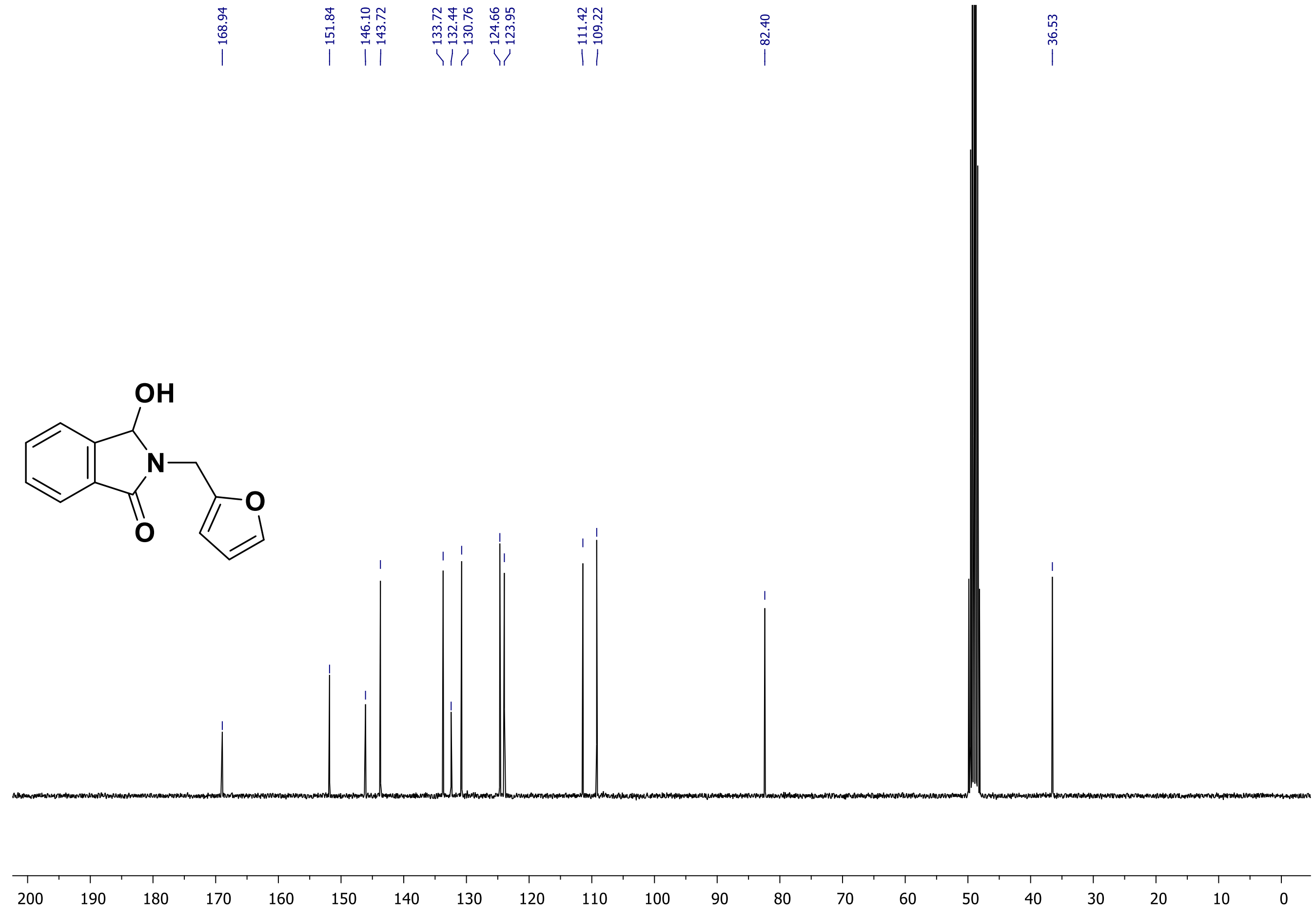

**$^1$H NMR** (Compound **17**)

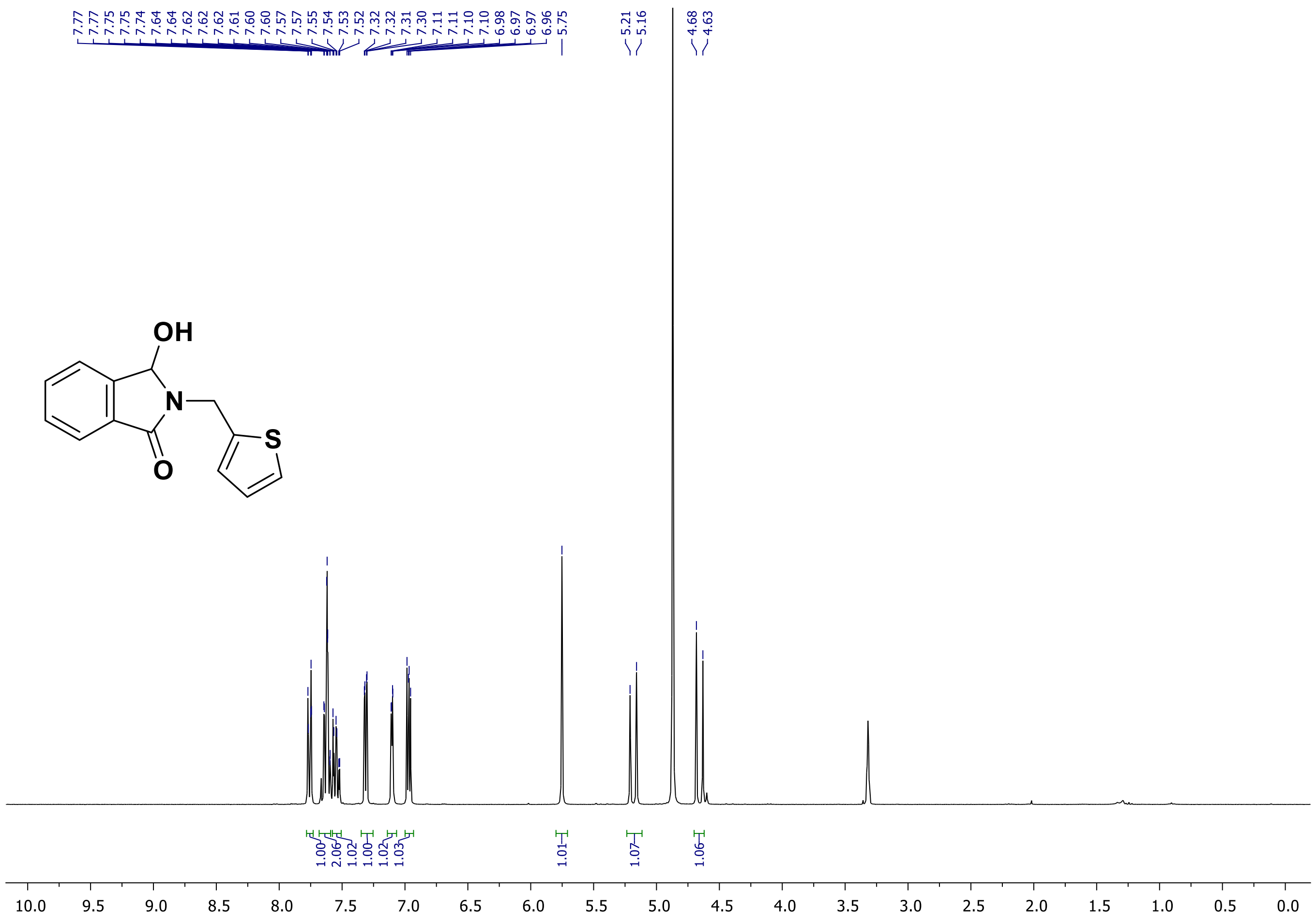


**$^{13}$C NMR** (Compound **17**)

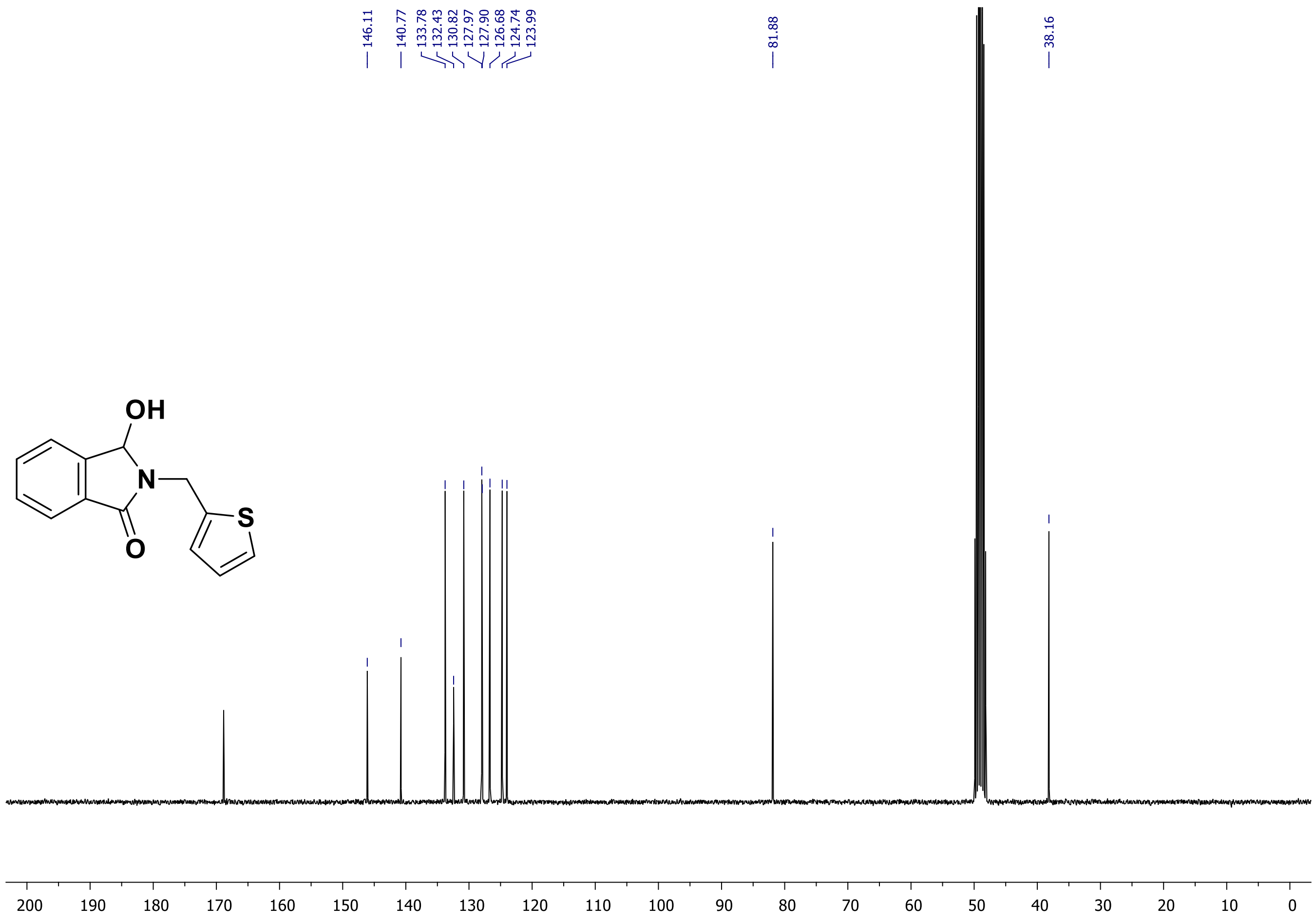

**$^1$H NMR** (Compound **18**)

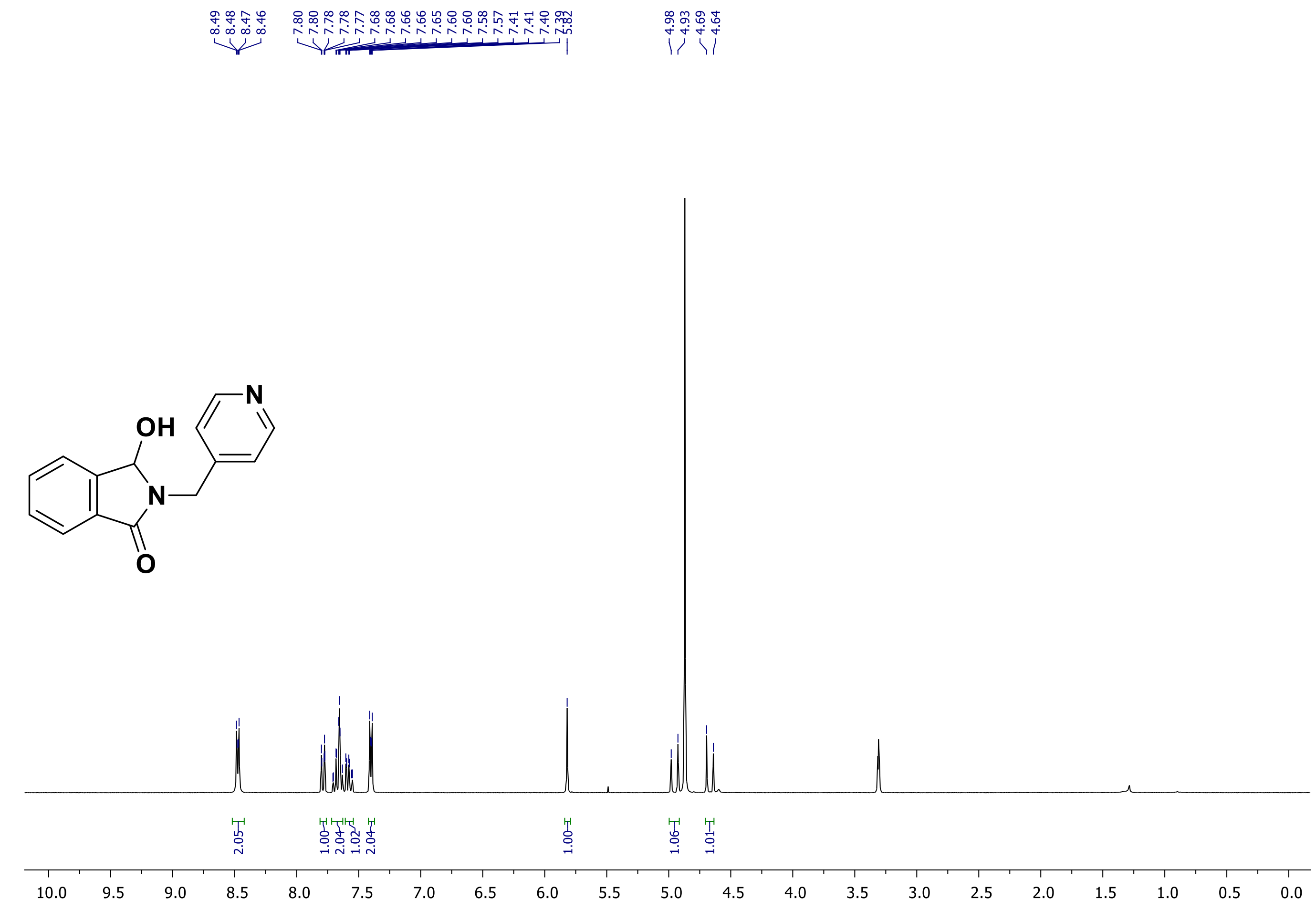


**$^{13}$C NMR** (Compound **18**)

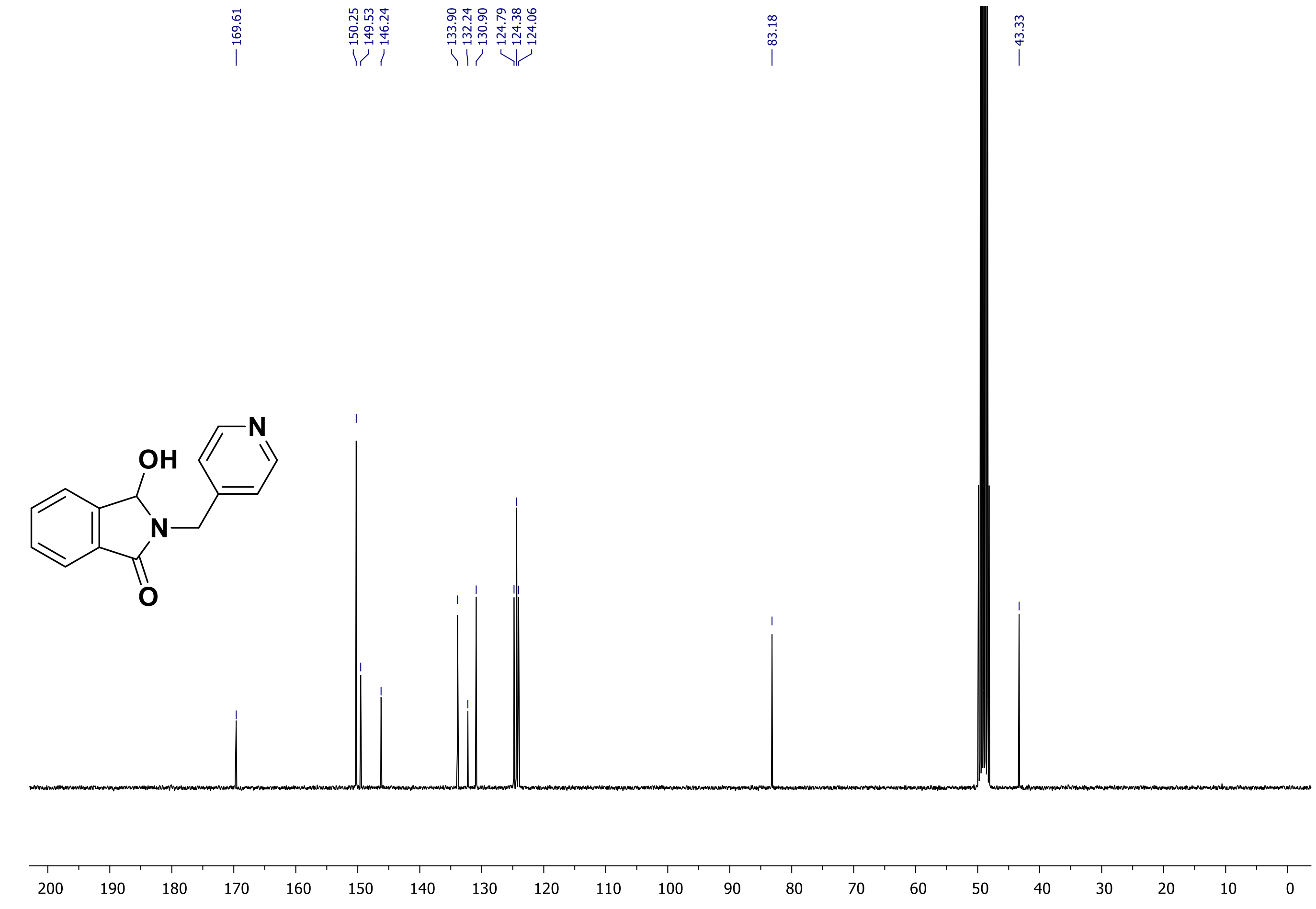

$^{1}$H NMR (Compound **19**)

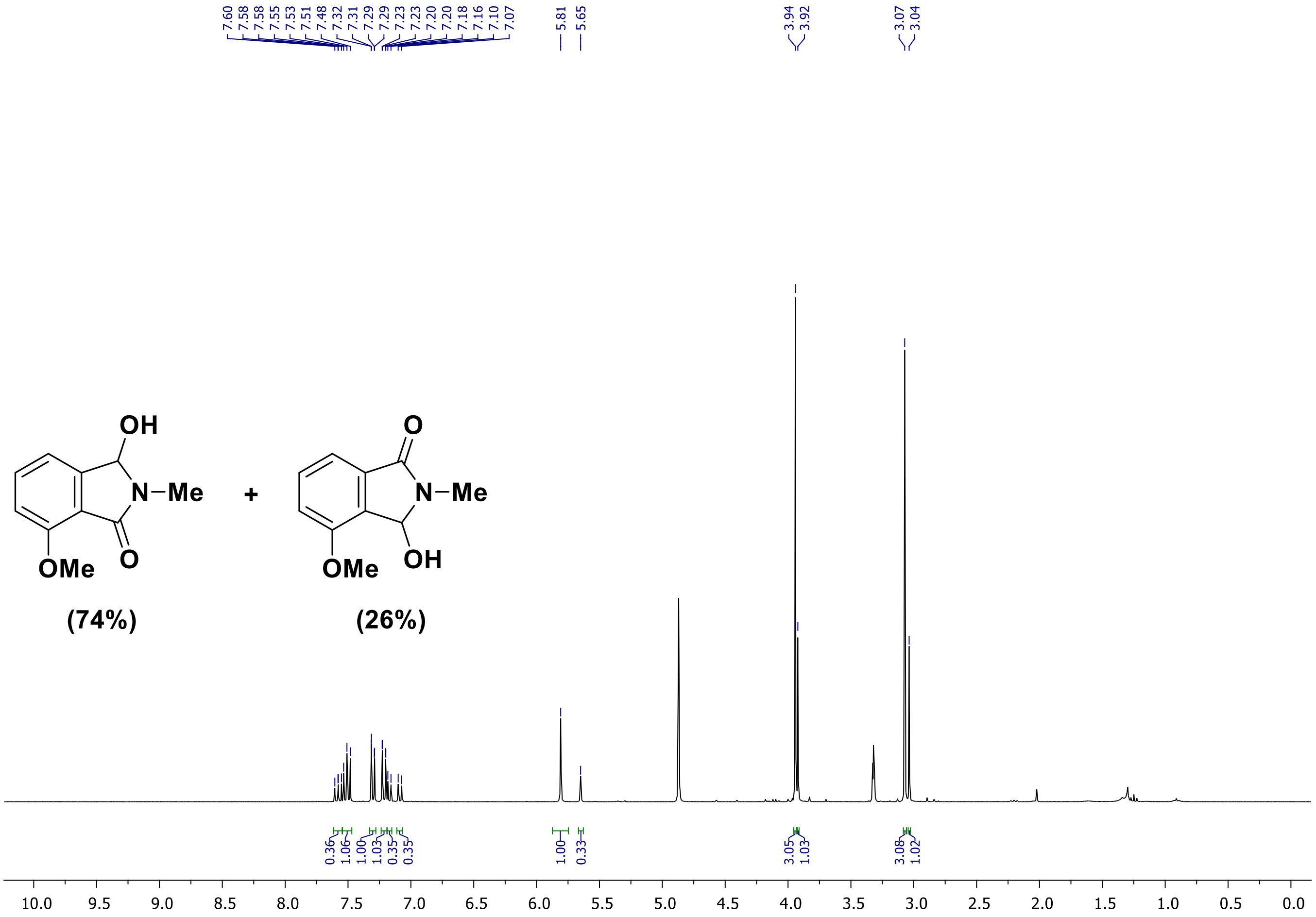


$^{13}$C NMR (Compound **19**)

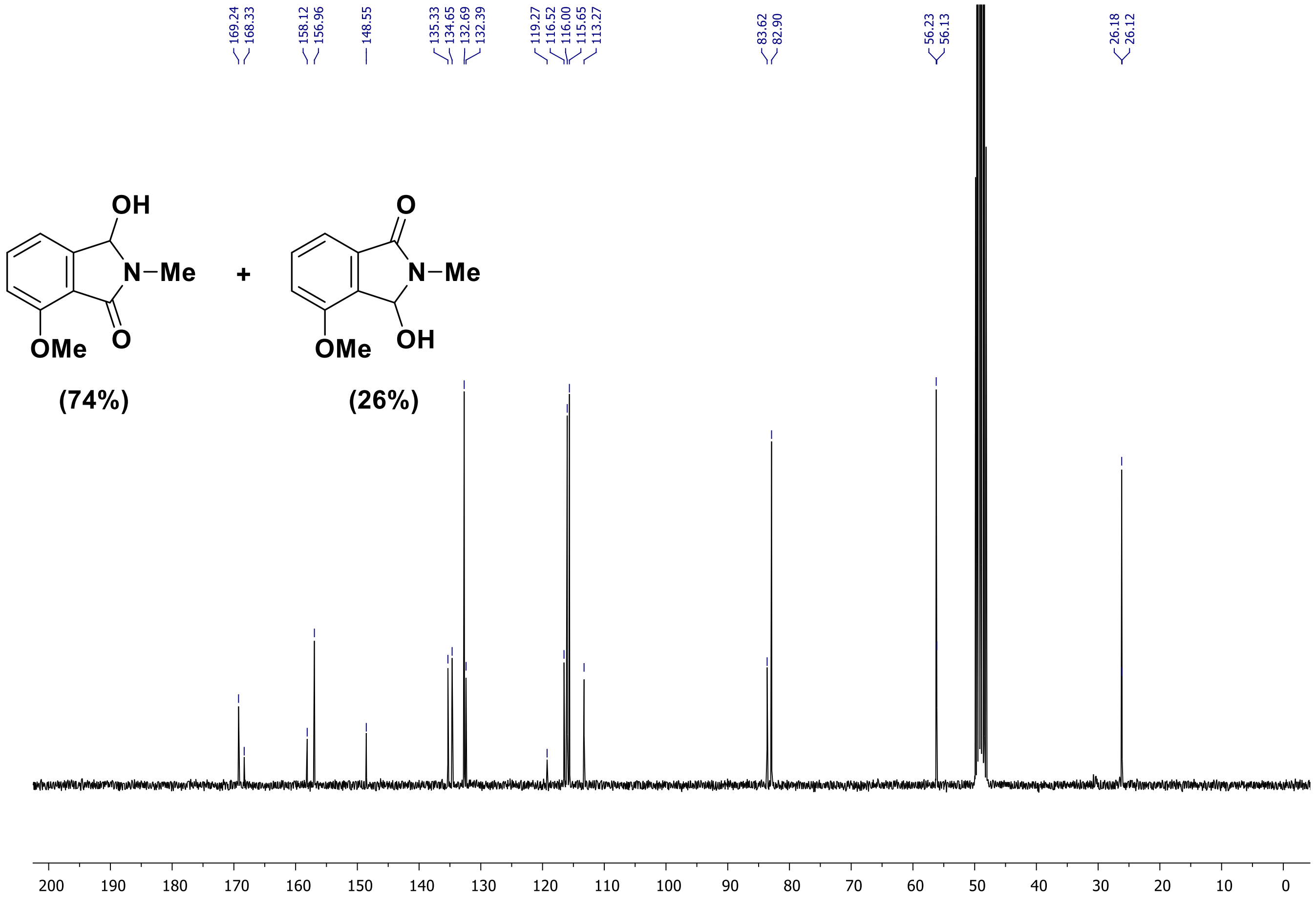

**HSQC** (Compound **19**)

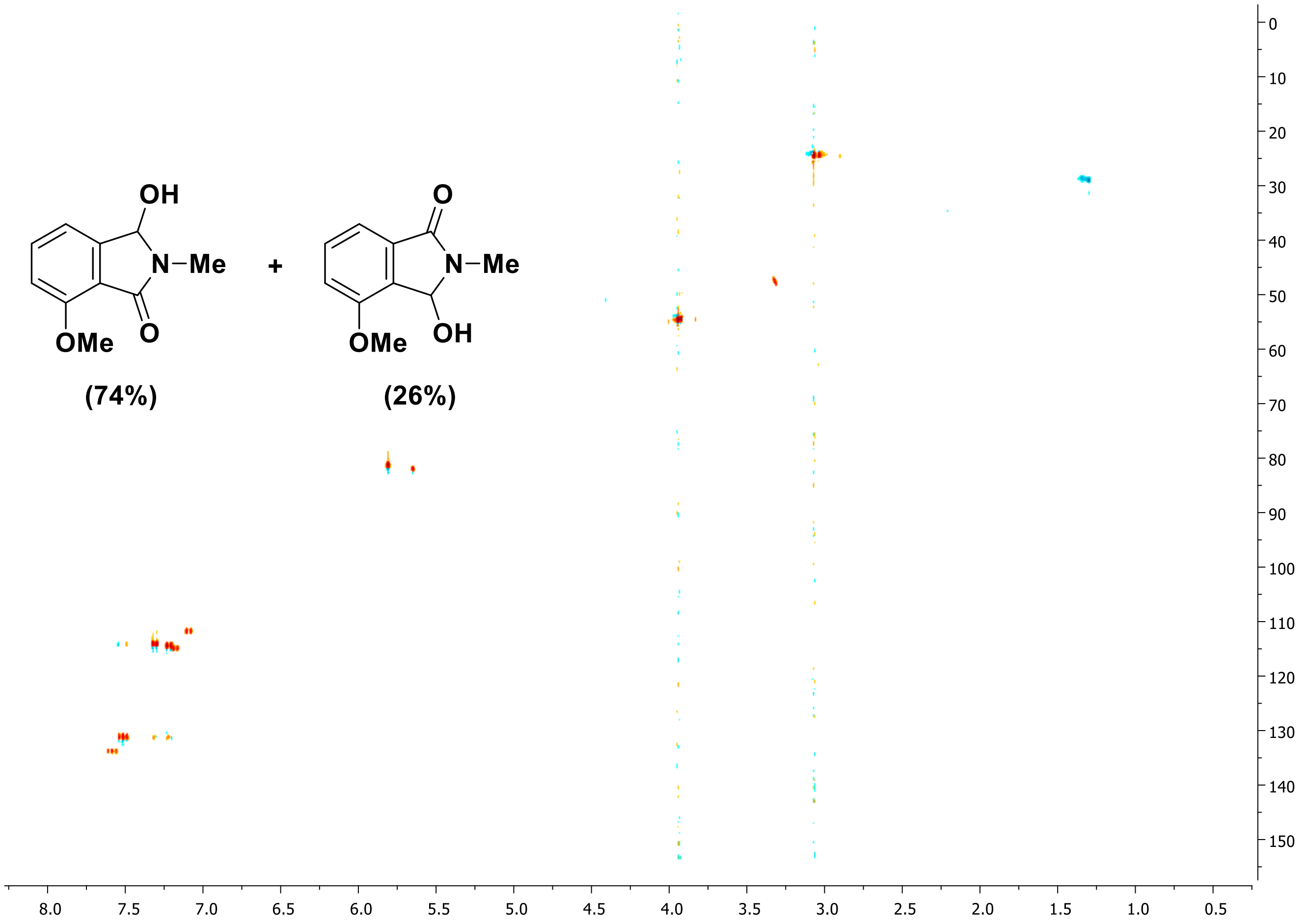


**HMBC** (Compound **19**)

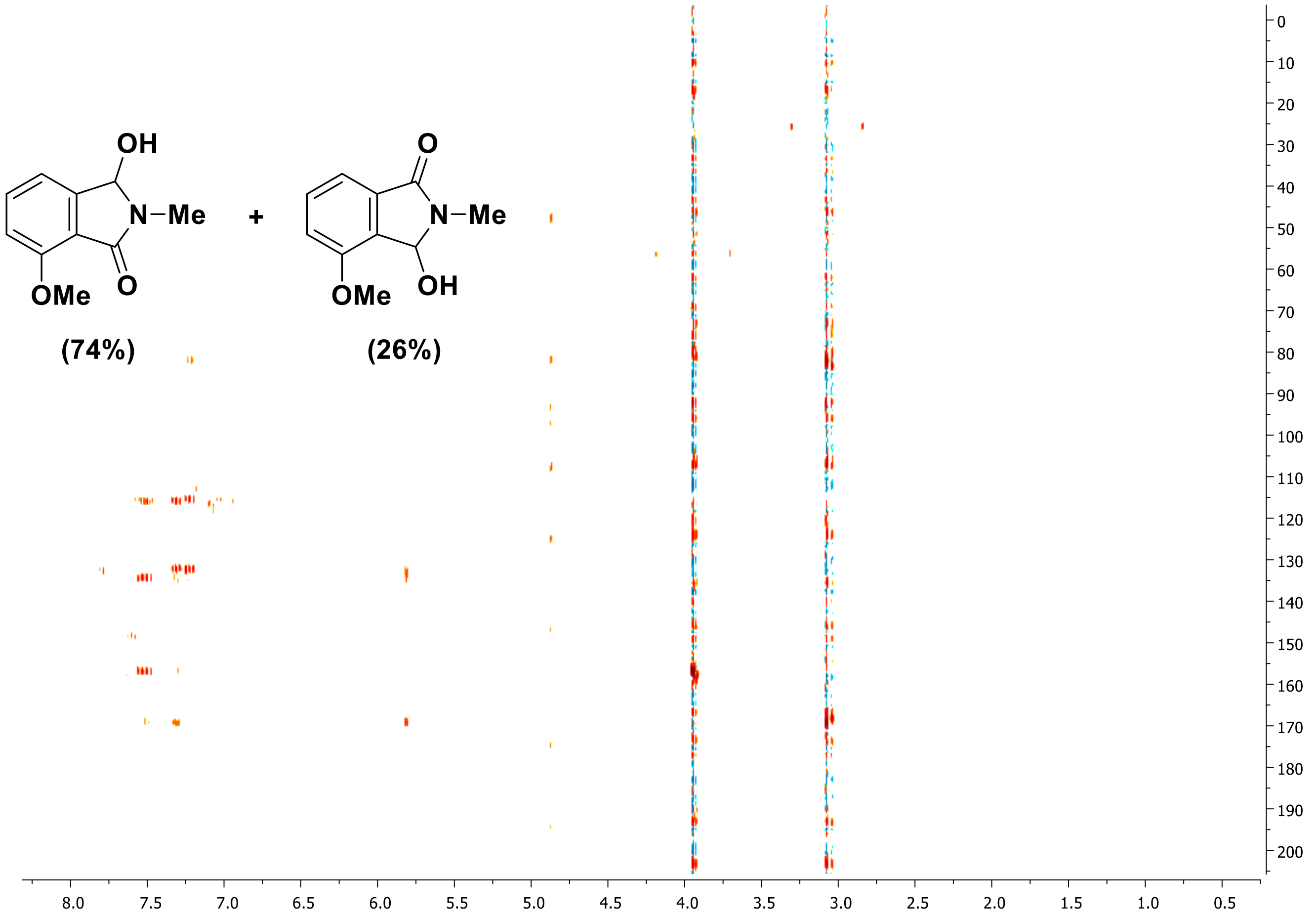

**$^{1}$H NMR** (Compound **20**)

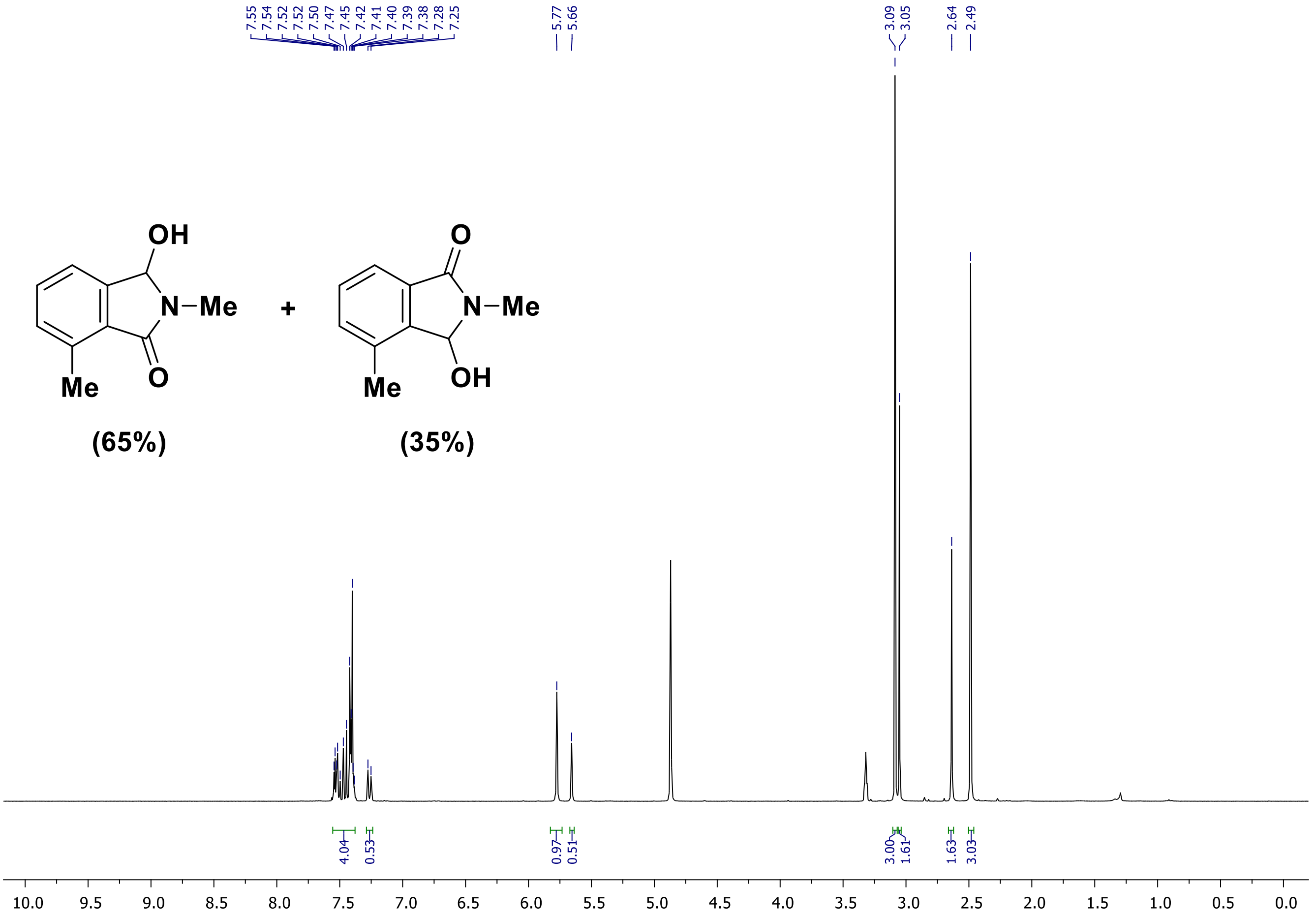


**$^{13}$C NMR** (Compound **20**)

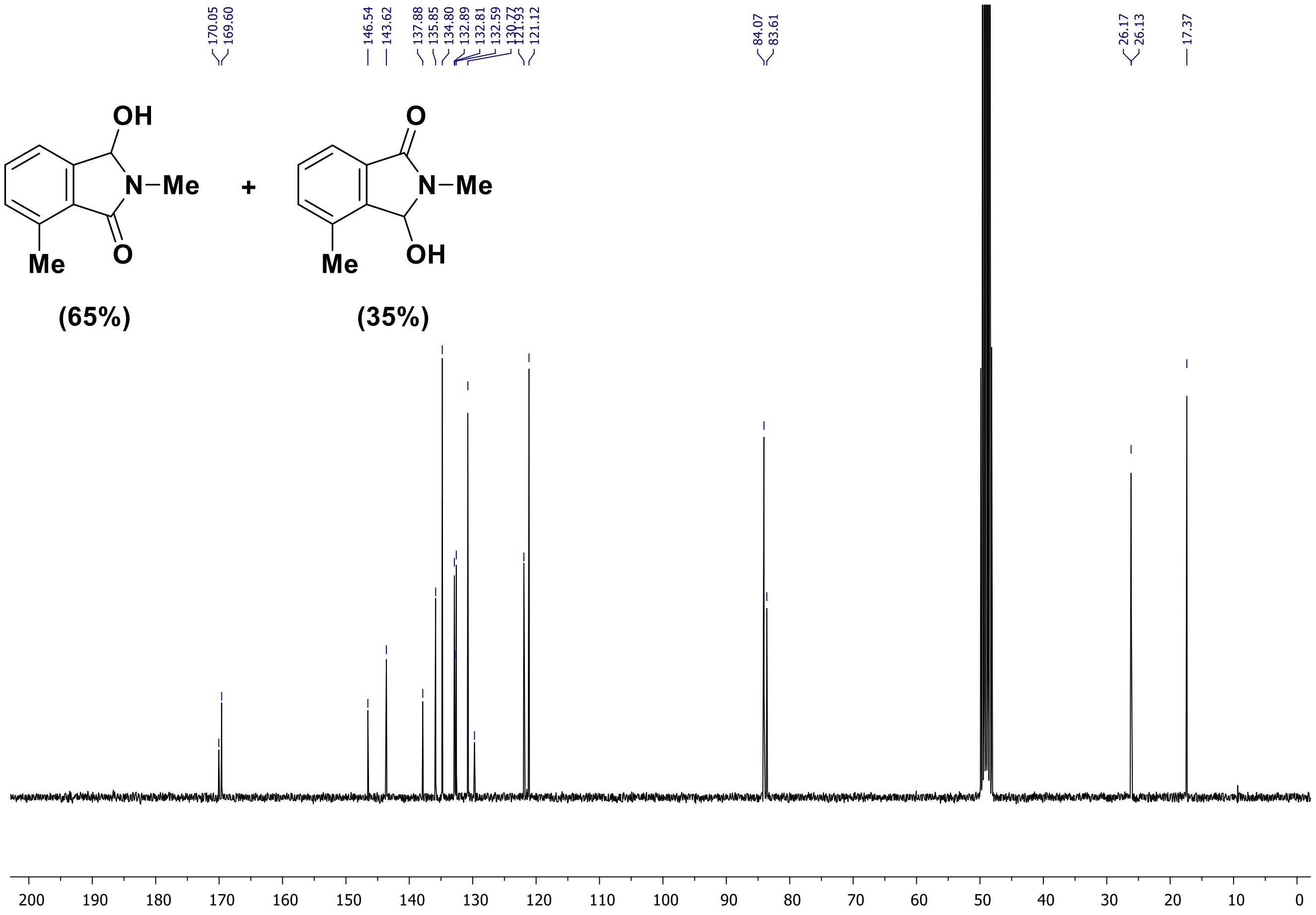

**HSQC** (Compound **20**)

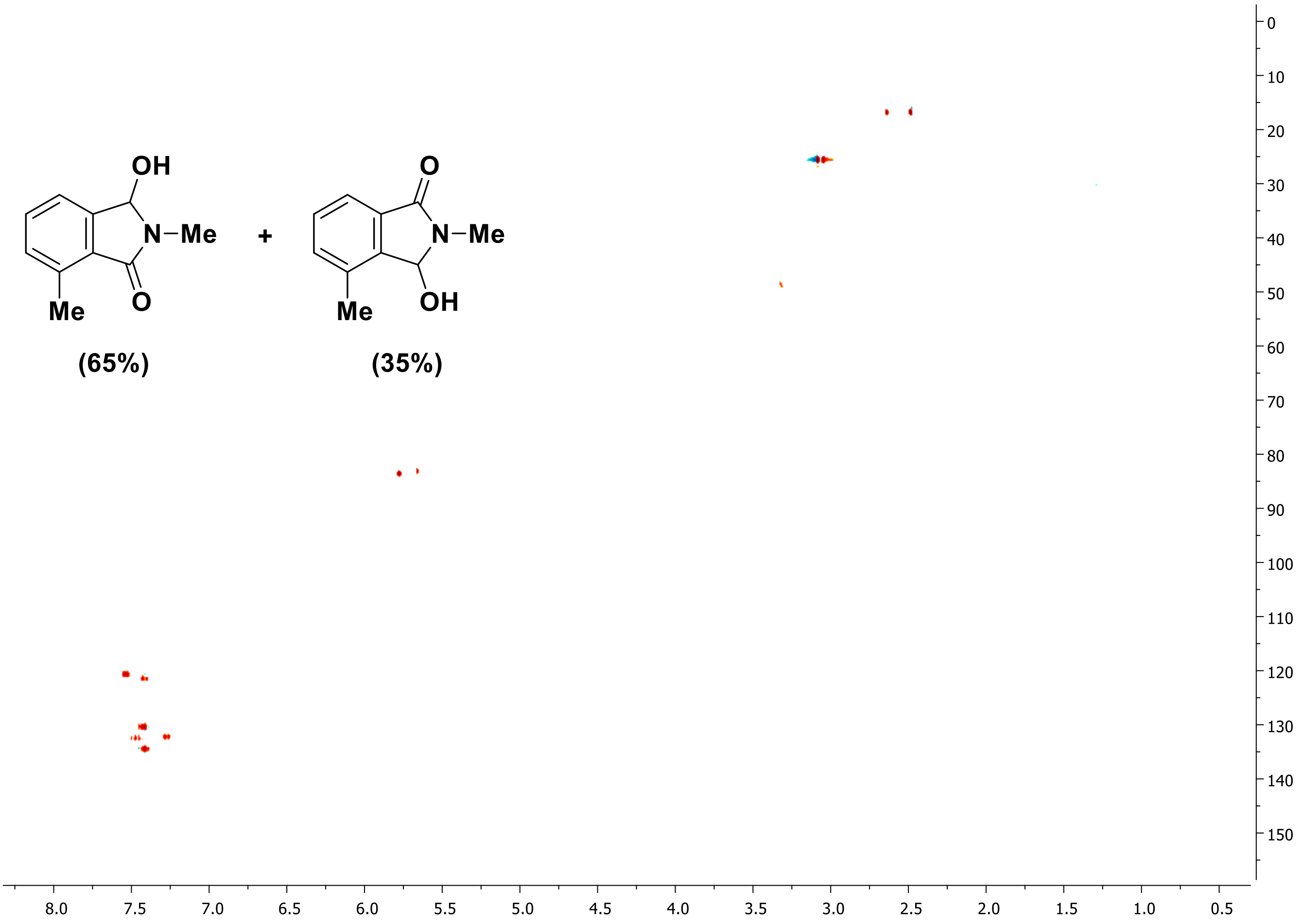


**HMBC** (Compound **20**)

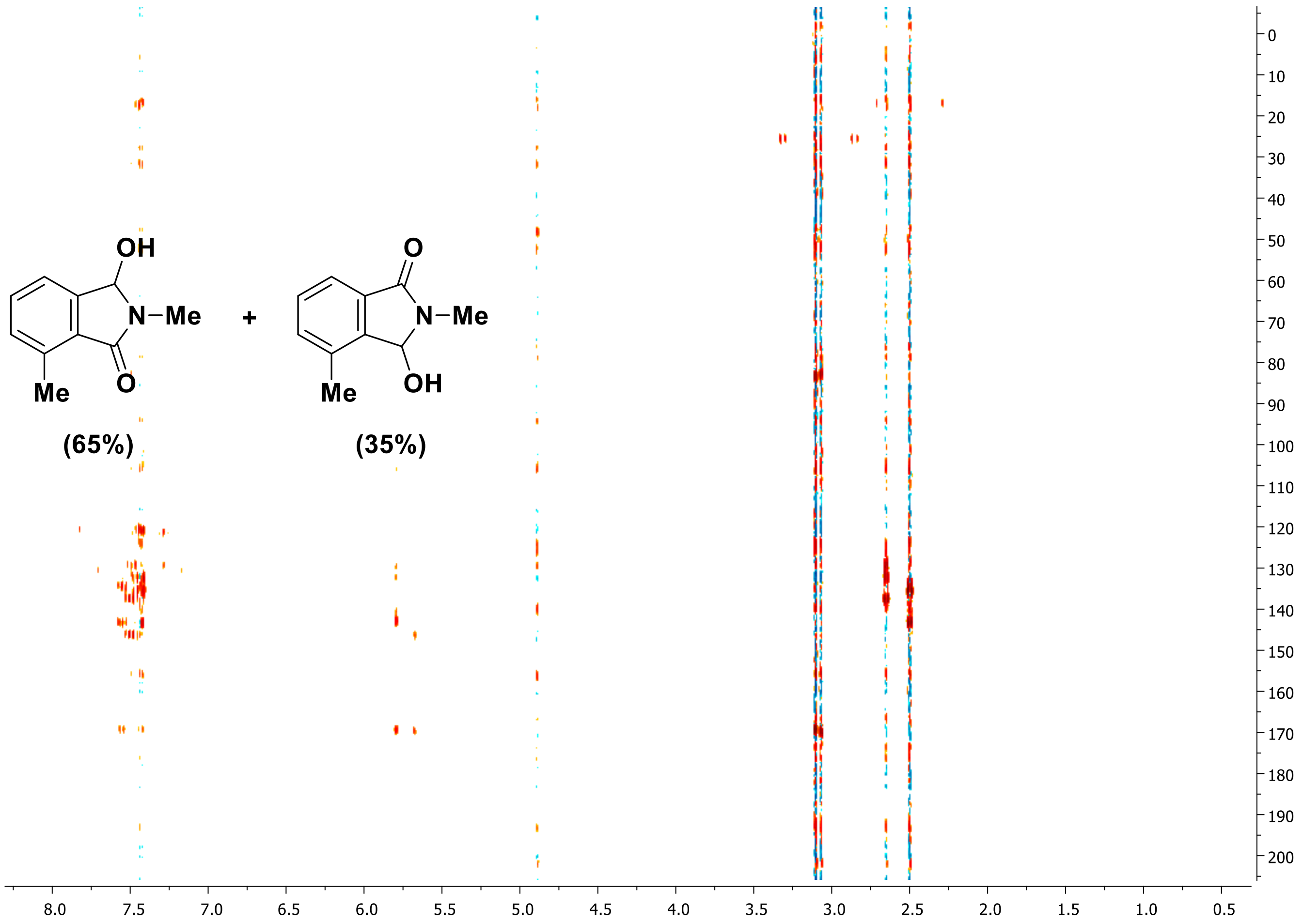

## 8. SUPPLEMENTARY REFERENCES


[1] C. A. Emeis, "Determination of Integrated Molar Extinction Coefficients for Infrared Absorption Bands of Pyridine Adsorbed on Solid Acid Catalysts" *J. Catal.* **1993**, *141*, 347-354.

[2] J. C. Groen, L. A. A. Peffer, J. Pérez-Ramírez, "Pore Size Determination in Modified Micro- and Mesoporous Materials. Pitfalls and Limitations in Gas Adsorption Data Analysis" *Microporous Mesoporous Mater.* **2003**, *60*, 1-17.

[3] N. Bogdanchikova, F. C. Meunier, M. Avalos-Borja, J. P. Breen, A. Pestryakov, "On the Nature of the Silver Phases of Ag/$Al_2O_3$ Catalysts for Reactions Involving Nitric Oxide" *Appl. Catal., B* **2002**, *36*, 287-297.

[4] A. S. Lanje, S. J. Sharma, Ramch, R. Pode, "Synthesis of Silver Nanoparticles: A Safer Alternative to Conventional Antimicrobial and Antibacterial Agents" *J. Chem. Pharm. Res.* **2010**, *2*, 478-483.

[5] M. H. Ali, M. A. K. Azad, K. A. Khan, M. O. Rahman, U. Chakma, A. Kumer, "Analysis of Crystallographic Structures and Properties of Silver Nanoparticles Synthesized using PKL Extract and Nanoscale Characterization Techniques" *ACS Omega* **2023**, *8*, 28133-28142.

[6] S. Ouyang, Z. Li, Z. Ouyang, T. Yu, J. Ye, Z. Zou, "Correlation of Crystal Structures, Electronic Structures, and Photocatalytic Properties in a Series of Ag-Based Oxides: $AgAlO_2$, $AgCrO_2$, and $Ag_2CrO_4$" *J. Phys. Chem. C* **2008**, *112*, 3134-3141.

[7] Y. Bai, L. Shi, L. Zheng, S. Ning, X. Che, Z. Zhang, J. Xiang, "Electroselective and Controlled Reduction of Cyclic Imides to Hydroxylactams and Lactams" *Org. Lett.* **2021**, *23*, 2298-2302.

[8] S. M. Abdallahi, E. F. Ewies, M. El-Shazly, B. Ould Elemine, A. Hadou, J. Moncol, A. M. Lawson, A. Daich, M. Othman, "Autotandem Catalysis: Inexpensive and Green Access to Functionalized Ketones by Intermolecular Iron-Catalyzed Amidoalkynylation/Hydration Cascade Reaction via *N*-Acyliminium Ion Chemistry" *Chem. Eur. J.* **2021**, *27*, 15440-15449.

[9] F. Pin, S. Comesse, B. Garrigues, Š. Marchalín, A. Daïch, "Intermolecular and Intramolecular α-Amidoalkylation Reactions using Bismuth Triflate as the Catalyst" *J. Org. Chem.* **2007**, *72*, 1181-1191.

[10] P. Pigeon, B. Decroix, "Synthesis of Thieno[2′,3′(3′,4′ or 3′,2′):5,6]azepino[2,1-*a*]isoindolediones from *N*-Thienyl-2(3)-ylmethylphthalimides" *J. Heterocycl. Chem.* **1996**, *33*, 129-135.
